# DNA sequence-level analyses reveal potential phenotypic modifiers in a large family with psychiatric disorders

**DOI:** 10.1038/s41380-018-0087-4

**Published:** 2018-06-07

**Authors:** Niamh M. Ryan, Jayon Lihm, Melissa Kramer, Shane McCarthy, Stewart W. Morris, Aleix Arnau-Soler, Gail Davies, Barbara Duff, Elena Ghiban, Caroline Hayward, Ian J. Deary, Douglas H. R. Blackwood, Stephen M. Lawrie, Andrew M. McIntosh, Kathryn L. Evans, David J. Porteous, W. Richard McCombie, Pippa A. Thomson

**Affiliations:** 1 Centre for Genomic and Experimental Medicine, MRC Institute of Genetic and Molecular Medicine, University of Edinburgh, Edinburgh, UK; 2 Stanley Institute for Cognitive Genomics, Cold Spring Harbor Laboratory, New York, USA; 3 Centre for Cognitive Ageing and Cognitive Epidemiology, Department of Psychology, University of Edinburgh, Edinburgh, UK; 4 Division of Psychiatry, University of Edinburgh, Royal Edinburgh Hospital, Edinburgh, UK; 5 MRC Human Genetics Unit, MRC Institute of Genetic and Molecular Medicine, University of Edinburgh, Edinburgh, UK; 6 Generation Scotland, Centre for Genomic and Experimental Medicine, Institute of Genetics and Molecular Medicine, University of Edinburgh, Edinburgh, UK

## Abstract

Psychiatric disorders are a group of genetically related diseases with highly polygenic architectures. Genome-wide association analyses have made substantial progress towards understanding the genetic architecture of these disorders. More recently, exome- and whole-genome sequencing of cases and families have identified rare, high penetrant variants that provide direct functional insight. There remains, however, a gap in the heritability explained by these complementary approaches. To understand how multiple genetic variants combine to modify both severity and penetrance of a highly penetrant variant, we sequenced 48 whole genomes from a family with a high loading of psychiatric disorder linked to a balanced chromosomal translocation. The (1;11)(q42;q14.3) translocation directly disrupts three genes: *DISC1*, *DISC2*, *DISC1FP* and has been linked to multiple brain imaging and neurocognitive outcomes in the family. Using DNA sequence-level linkage analysis, functional annotation and population-based association, we identified common and rare variants in *GRM5* (minor allele frequency (MAF) > 0.05), *PDE4D* (MAF > 0.2) and *CNTN5* (MAF < 0.01) that may help explain the individual differences in phenotypic expression in the family. We suggest that whole-genome sequencing in large families will improve the understanding of the combined effects of the rare and common sequence variation underlying psychiatric phenotypes.

## Introduction

Schizophrenia (SCZ), bipolar disorder (BD) and major depressive disorder (MDD) are debilitating psychiatric disorders, with MDD ranked by the World Health Organisation as the single largest contributor to global disability [1]. Family, twin and adoption studies have shown a strong heritable component to these disorders [2, 3], with heritability estimates of 0.37 for MDD, 0.75 for BD and 0.81 for SCZ [4]. Genome-wide association studies (GWAS) have identified over a hundred common single nucleotide polymorphisms (SNPs) robustly associated with these disorders, most for SCZ, but all with small effect sizes [5–7]. Individually rare copy number variants from case–control studies and exome sequencing-derived missense variants with large effects have also been reported [8–12]. However, even within families, individuals with these variants show variability in clinical phenotype [13–16]. Thus, the evidence to date suggests that psychiatric disorders have a complex and heterogeneous genetic architecture, with many potential genetic routes leading to the same clinical outcome, some variants associated with a broad range of conditions, and with both common and rare variants playing a role [4, 17].

Family-based studies can be used to study the effects of common and rare variants in the context of a less heterogeneous genetic background, and could allow us to better understand the intra-family variation and reduced penetrance commonly seen in complex traits. We have analysed the whole-genome sequences of 48 out of 107 members of a large Scottish family with a high loading of broad spectrum major mental illness. Members of the family carry a balanced translocation between chromosomes 1 and 11 (1q42; 11q14.3, hereafter t(1;11)) that is significantly linked to major mental illness, with a maximum logarithm of the odds (LOD) score of 7.9 for a broad model including individuals with SCZ, BD, schizoaffective disorder (SCZAFF), recurrent MDD (rMDD), cyclothymia, single episode MDD (sMDD) plus minor diagnoses (including: alcoholism, adolescent conduct disorder and anxiety) [18]. However, nine family members without the translocation also have psychiatric diagnoses, whereas two carriers show no evidence of psychiatric disorder, suggesting that additional factors may be involved in the clinical phenotype. We hypothesised that the variation in phenotypic expression in the family may reflect the inheritance of the translocation and a variable subset of predisposing/modifier variants. We combined genome-wide variance component multipoint linkage, regional two-point linkage and haplotype analyses on the full spectrum of variants within the t(1;11) family to identify additional potential risk/modifier loci and tested the association of these loci in two case–control samples drawn from the UK population.

## Materials and methods

The materials and methods are described in full in the Supplementary Information.

Details of the translocation family are given in Thomson *et al.* [18] (see also: Supplementary methods: Diagnoses and phenotypic models in the family). This study was approved by the Multi-Centre Research Ethics Committee in Scotland (09/MRE00/81). All study participants gave their written, informed consent.

DNA samples were sequenced to a median coverage of > 30 over three lanes of a HiSeq2000 using 101 bp paired-end reads. After local realignment around indels and base quality score recalibration of each library, single-nucleotide variants (SNVs) were called with GATK v2.4.9 [19-21], using the multi-sample joint calling mode to achieve consistent calling across samples (Supplementary table 1: GATK summary table; Supplementary figure 1a: Individual sample coverage; Supplementary methods: WGS sequencing and variant calling). Variant quality score recalibration (VQSR) parameters were applied as recommended in the GATK best practices documentation for GATK v2.4.9. The “truth sensitivity filter level” was set at 99.0. Deletions ≥ 1 kb that map to a single genomic location were detected by event-wise testing based on read depth [22] (Supplementary methods: Copy Number Variation (CNV) calling).

Validation of GATK VQSR modelling showed that 94% pass variants and 52% of failed variants were validated by custom designed Taqman assays (Supplementary methods: Variant validation-VQSR filter). All variants were therefore retained initially and an additional filter for Mendelian segregation in the family applied.

Variance component linkage analyses were performed using the SOLAR software package [23] (Supplementary methods: Linkage analysis). LOD scores were adjusted for deviation of the phenotype distribution from normal but, due to the nested nature of the phenotypes, were not adjusted for multiple testing. SNVs within the genomic regions under the linkage peaks were phased using the software SHAPEIT (v2.r83725) and the 1000 Genomes Phase 1 integrated reference panel26, incorporating the pedigree information to increase accuracy (Supplementary methods: Haplotype phasing). Minimal haplotype regions were defined by recombination breakpoints in the affected individuals in the family. Mixed logistic or linear regression models, fitting the inverse relationship matrix as a random effect to control for familial structure, were used to test phenotype associations of haplotypes using ASREML-R (www.vsni.co.uk/software/asreml). The significance of fixed effects within the model was assessed using a conditional Wald F-test. A model *p-*value of < 0.05 was considered significant. In addition to family structure, age and sex were fitted in analyses of global assessment of functioning (GAF), current IQ and attention/processing speed.

Association studies of affective disorder and related traits were performed in two population-based cohorts: Generation Scotland: Scottish Family Health Study (GS:SFHS) [24–26] and UK Biobank (UKB) [27], fitting principal components and cohort/phenotypes appropriate covariates using subsets of unrelated individuals (see Supplementary methods: UK Population-based cohorts: GS:SFHS and UKB and region-wide association analyses).

## Results

### Whole-genome sequencing in the translocation family

Whole-genome sequencing was performed on comprising all 48 individuals from the t(1;11) family for whom DNA was available. The final dataset included 9.76 million SNVs present in at least one individual of which 16.46 and 5.80% were not found in the 1000 Genomes phase 3 European [28] or gnomAD non-Finnish European subsets, respectively, and 5.41% were found in neither repository (July 1017; Supplementary figure 1b: Minor allele frequencies (MAF) of SNVs in 1000 Genomes phase 3 European sample (EUR) and GnomAD non-Finnish European (NFE)). In addition, using a read depth approach, 27 unique deletions were identified with sizes ranging from 1.2 to 145 kb.

### Evidence for the effects of additional regions on risk of psychiatric disorder in the family

To study the genetic contribution to the range of diagnoses present in this family, nine phenotypic models were evaluated using genome-wide linkage analyses of the 48 t(1;11) family members; of whom 19 are translocation carriers (18 affected; 1 unaffected) and 29 non-carriers (6 affected; 22 unaffected; 1 unknown). Table 1 shows the numbers of cases and controls included in each model (Supplementary Table 2: Phenotype models split by translocation status). The maximum theoretical LOD (mtLOD) and the observed two-point LOD for the translocation was generated for each model using only the set of family members sequenced in this study. The translocation explained the majority of the mtLOD, particularly for Model A: SCZ, BD and SCZAFF (number affected = 6, 6 translocation carriers, 0 non-carriers) and Model H: SCZ and SCZAFF (number affected = 4, 4 translocation carriers, 0 non-carriers). However, the translocation LOD explained less than half of the mtLOD for affective disorders (Model F; number affected = 14, 9 translocation carriers, 5 non-carriers), Psychosis (number affected = 8, 8 translocation carriers, 0 non-carriers), and the models containing minor diagnoses (Model C and Model D; Table 1). This implies additional segregating genetic factors, over and above the translocation, which impact on the clinical presentation for these models.

**Table 1:** Summary of the phenotypic models in the 48 individuals from the t(1;11) family

Model	Diagnoses	Affected	Controls	NA	T-aff	NT-aff	mtLOD	t(1;11)
Model A	SCZ, BD, SCZAFF	6	23	19	6	0	3.2	2.8
Model B	SCZ, BD, SCZAFF, rMDD, cyclothymia	15	23	10	13	2	8.6	6.7
Model C	SCZ, BD, SCZAFF, rMDD, cyclothymia, MDD, adolescent conduct disorder, generalised anxiety disorder, alcoholism	24	23	1	18	6	12	5.8
Model D	MDD, adolescent conduct disorder, generalised anxiety disorder, alcoholism	9	23	16	5	4	5.8	1.3
Model E	SCZ, BD, SCZAFF, rMDD	12	23	13	10	2	6.9	4.8
Model F	BD, rMDD, MDD	14	23	11	9	5	8.9	4.2
Model G	BD, rMDD	8	23	17	6	2	5.7	3.1
Model H	SCZ, SCZAFF	4	23	21	4	0	2.4	2.2
Psychosis	Psychosis any diagnosis	8	23	17	8	0	4.4	1.9
Total		24	23	1	19	29		

mtLOD, maximum theoretical LODs; t(1;11), the two-point LOD score for the translocation; number of affected translocation carriers (T-aff) and affected non-carriers (NT-aff), further details are given in Supplementary Table 2

**Fig. 1:**
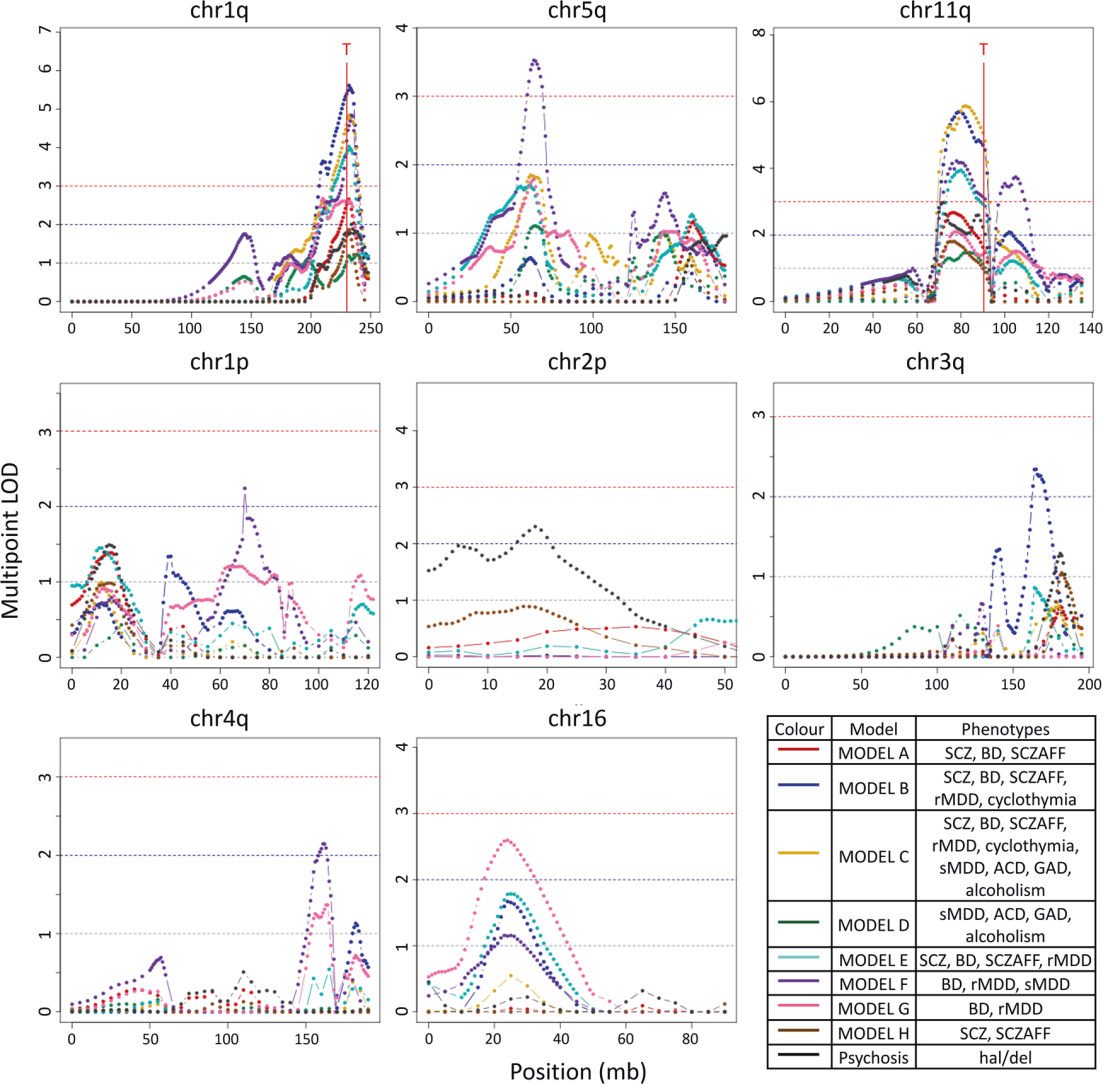
Multipoint linkage analysis of the t(1;11) family. Plots of the chromosomes with multipoint linkage peaks with LOD ≥ 2 and a summary table of the phenotype models (the colour coding reflecting the colours of the models in the multipoint plots). The plots represent multipoint LOD scores vs. chromosome position in Mb. Multipoint LODs = 1, 2 and 3 annotated with grey, blue and red horizontal dotted lines, respectively. The translocation breakpoints, T, are marked by a red vertical line. For plots of all chromosomes, see Supplementary Figure 2

Multipoint linkage analyses identified four genome-wide significant (LOD ≥ 3.3) peaks: three on the translocation chromosomes: one located on chr1q and two peaks on chr11q (chr11q1 and chr11q2); and a fourth peak on chr5q (Supplementary methods; Fig. 1, Table 2). The chr1q and the chr11q1 linkage peaks show evidence for linkage across all models, with maximum LODs for the broad phenotypic models (Model C: any psychiatric diagnosis, and Model B: SCZ, BD, SCZAFF, rMDD and cyclothymia). In contrast, the chr11q2 and chr5q peaks were only significant for the affective disorder phenotype (Model F: BD, rMDD and MDD). Five further regions with LOD ≥ 2 were also identified, each of which was specific to a phenotype model (Fig. 1, Table 2; plots for all chromosomes are given in Supplementary Figure 2).

**Table 2:** Summary of multipoint and two-point linkage analyses

Region	Start	End	Size	Max mLOD	Model	*N* genes	Max 2pt LOD	Model	Top 2pt variant	Closest gene distance
chr1q	223,246,661	224,932,082	1,685,421	5.6	B	14	9.3	C	chr1: 223,804,982	[*CAPN8*] intronic
chr5q	58,721,609	67,098,280	8,376,671	3.5	F	33	5.1	F	rs377870	[*C5orf64*] intronic
chr11q1	87,597,790	88,350,687	752,897	5.9	C	3	10.8	C	chr11: 88,219,524	[]*GRM5* 18,221 bp
chr11q2	99,616,360	99,753,867	137,507	3.7	F	1	8.5	F	chr11: 99,653,622	[*CNTN5*] intronic
chr1p	69,000,000	71,000,000	2,000,000	2.2	F	7	2.3	F	rs149129136	[]*LRRC7* 151,267 bp
chr2p	17,768,000	18,351,000	583,000	2.3	P	5	2.3	E	rs4832502	[]*KCNS3* 24,483 bp
chr3q	162,000,000	172,000,000	10,000,000	2.3	F	36	3.6	C	chr3: 164,306,631	[]*SI* 390,056 bp
chr4q	158,000,000	164,000,000	6,000,000	2.2	F	15	3.6	F	rs149639769	[]*FSTL5* 1,432,353 bp
chr16p	16,000,000	33,000,000	17,000,000	2.6	G	222	3.4	G	chr16: 23,353,022	[*SCNN1B*] intronic

Start and end position given for hg19; Max mLOD, maximum multipoint LOD score; Top 2 pt LOD, two-point LOD score; and the closest gene to the highest 2 pt LOD with the brackets denoting the relative position. Model definitions are shown in Table 1

### Minimum haplotype regions do not span the translocation breakpoints but contribute to prediction of psychiatric disorder in the family

**Fig. 2:**
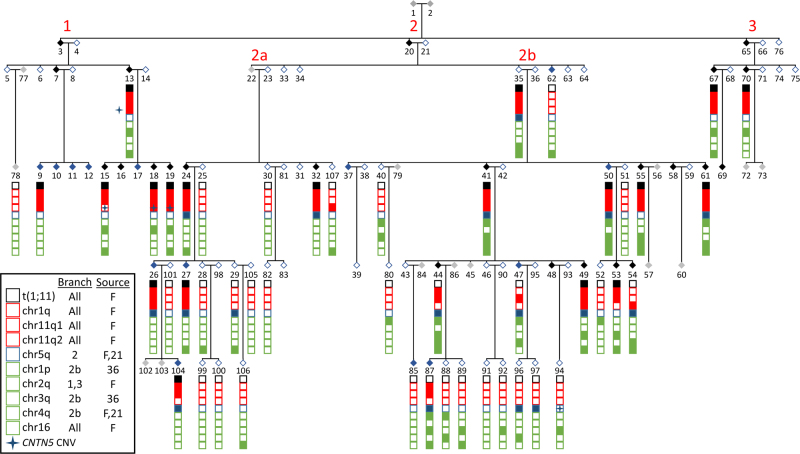
Segregation of linked haplotypes within the pedigree. Full pedigree showing affected status under Model B (SCZ, BP,  rMDD and Cyclothymia; open—unaffected, black—affected, blue—other diagnoses, grey—unknown) and carrier status of phenotype-linked haplotypes in sequenced individuals (boxes: filled—haplotype carrier, open—non-carriers). Blue star—CNTN5 CNV carriers. Red numbering—sub-branches of the pedigree. Legend shows, which sub-branches contain the linked haplotypes and the source of the haplotype: F— familial or married-in ID

The minimum haplotype regions were defined for each genome-wide significant peak and the diagnoses of the carriers identified (Supplementary Figure 3). Table 2 shows the boundaries of these linkage regions. Information on all the genes in the regions is given in Supplementary Table 3. The minimum haplotypes under the linkage peaks on chromosomes 1 and 11 (chr1q, chr11q1 and chr11q2), although adjacent to the translocation, do not include the breakpoints (hg19: chr1: 231,950,368 and chr11: 90,361,108; Supplementary Figure 3a and 3b). The maximum multipoint LOD scores in the region of the translocation are driven both by individuals with the translocation, who share a wide flanking region, and by individuals with psychiatric diagnoses who carry discrete regions shared with the translocation carriers (Supplementary Information: Haplotype phasing). The combination of haplotypes carried by each family member and their diagnosis are shown in Supplementary Table 4 (see also Fig. 2). The chr1q haplotype and the chr11q1 haplotypes are defined by all 19 of the translocation carriers and additional affected individuals who do not carry the translocation (Supplementary Figures 3a and 3b). The chr11q2 haplotype is not present in three of the translocation carriers; this region is defined by recombination events in two translocation carriers (ID 18 and 19), beyond the recombination event inherited from the married-in parent, and an individual with rMDD who does not carry the translocation. The chr5q haplotype is shared by 10 translocation carriers and 7 non-carriers (Supplementary Figure 3c).

An indirect effect of the translocation in the family is to “lock together” the translocation, chr1q, chr11q1 and chr11q2 regions in translocation carriers. In translocation carriers, the minimum shared haplotypes spanning the breakpoints were defined (Supplementary Figures 2d and 2e). The haplotypes shared by the translocation carriers spanned 17.5 Mb on the derived chr1 and 22.2 Mb on the derived chr11. The size of these shared regions is consistent with the lack of evidence for reduced recombination around the translocation reported by He et al. [29].

Sequence-level analyses of the regions on chr1q and chr11q1, shared by the translocated chromosomes that have undergone recombination (IDs 18 and 19; Supplementary Figure 3b) and three non-translocation carriers (siblings with IDs 44 and 47, and the child of 44, ID 87; Fig. 2; Supplementary Table 4: Summary of haplotype segregation in the t(1;11) family), show that these haplotypes are near identical to those on the derived chromosomes. Although it is not possible to definitely show that these haplotypes are inherited from the familial parent, these regions span multiple haplotype blocks in the Scottish population and the combinations found on the derived chromosomes occur at <1% frequency, suggesting that these haplotype arose once in the family. One individual in the family appears to carry two copies that are 97.3% identical across the chr11q1 region suggesting that regions homologous to the chromosomes flanking the translocation are present within the Scottish population.

We next used mixed regression models to investigate the contribution of each haplotype to phenotype prediction. Given the relationships between the family members, and the total number of individuals, the correlation structure between the haplotypes is complex (Supplementary information: Haplotype correlations). This must be considered when interpreting the results of phenotypic prediction using backward selection, as the translocation event, chr1q and chr11q1 regions are likely to explain the same variance in phenotype and be retained randomly in the models (Supplementary Information: Regional correlations; Supplementary Figure 4: Intra-family regional correlations). Analyses of Model F (affective disorder) and Psychosis suggest that additional haplotypes (chr2p, chr3q and chr5q), in combination with the haplotypes on the derived chromosomes, may contribute to the phenotypic heterogeneity seen in the translocation family (Supplementary Information: Phenotype prediction; Supplementary Table 5a and 5c). Age of onset was available for 23 of 24 of the individuals with a diagnosis of affective disorder (Model F). The minimum models for this trait retained both chr11q2 and chr16p (Supplementary Table 5c). In analyses of translocation carriers only, no individual haplotype predicted phenotype; however, combined analyses of all haplotypes not on the translocated arms (chr5q–chr16p) did predict Model F and Psychosis. One of the haplotypes that contribute to Model F has entered the family through an unaffected individual who married into a sub-branch of the family (chr3q, sub-branch 2b, Fig. 2). In contrast, chr16p for Model F and chr2p for psychosis were present in the founders of the family, whereas it is unknown whether chr5q was inherited from the founders or through the non-familial parent founder of branch 2 (Fig. 2: Segregation of linked haplotypes within the pedigree).

### Fine-mapping linked regions using two-point linkage analyses

Detailed two-point linkage analysis was performed across the haplotype-associated regions under the four genome-wide significant multipoint linkage peaks and the five regions with multipoint LODs ≥ 2. Two-point linkage was performed on those variants that passed QC filters: Hardy–Weinberg equilibrium (HWE) *p* > 0.001 and present in at least three individuals with an additional raw case–control odds ratio filter for the LOD ≥ 2 regions (Supplementary Methods: Linkage analysis; Supplementary Figures 5 and 6: Regional two-point summaries). The top 100 LOD scores for each of the haplotype regions, and for all variants analysed in genome order, are listed in the Supplementary Tables 6a-e.

With the caveat that two-point linkage can be unstable if the population allele frequencies are mis-specified, the top two-point LOD scores and the respective model for each linkage region are shown in Table 2. No two-point LOD score (mLODs) achieved the maximum theoretical LOD scores (mtLODs, Table 1); however, the highest two-point LODs for the chr11q1 (mLOD = 10.8; mtLOD = 12, Model C: any diagnosis), chr11q2 (mLOD = 8.3; mtLOD = 8.9, Model F: affective disorder) and chr4q (mLOD = 4.7; mtLOD = 5.7, Model G: BD and rMDD) regions explain the majority of the maximum theoretical linkage signal. The four genome-wide significant linkage regions and chr16p achieved higher maximum two-point LODs than those of the translocation for the same models (Table 1). Examination of the two-point linkage results across the regions revealed multiple blocks of linkage in each region with evidence that different subregions show strongest linkage under different models (Supplementary Figures 5 and 6; Supplementary Information: Two-point linkage analyses). This may reflect the presence of multiple risk loci within each region.

Many GWAS significant SNPs have been identified in non-coding regions and are predicted to tag regulatory or missense variants [30–33]. Functional annotation of variants under the genome-wide significant linkage peaks detected evidence of missense variants and brain expression quantitative trait loci (eQTL) (Supplementary Information: Functional annotation of variants under the genome-wide significant linkage peaks, Supplementary Tables 6a-e). Variant effect prediction indicated that variants in *CAPN2*, *TP53BP2*, *NVL* in chr1q; and *CTSC* and *CNTN5* in chr11q1 and chr11q2, respectively, had a high probability of being damaging (Polyphen) or deleterious (SIFT; Supplementary Tables 7: Variant Effect Predictor annotation). High LOD SNVs and SNPs in regions affected by deletions were associated with eQTLs, with the strongest evidence in each region for the genes: *ENAH* (rs75866472), *GRM5* (rs10128749), *CNTN5* (intronic deletion: DGV id: esv2661704) and *PDE4D* (rs988827) in chr1q, chr11q1, chr11q2 and chr5q, respectively (Supplementary Information: Functional annotation of variants under the genome-wide significant linkage peaks, Supplementary Tables 8: Functional investigation of top LOD variants within the phenotype-associated haplotypes).

### Association analysis of the linkage regions in two samples from the Scottish population

Sequence-level analyses of the linked haplotypes indicated that these haplotypes may be present in the Scottish population, and may be independently associated with risk. Evidence for association between SNVs in the linked regions and affective disorder outside of the family (Model F, MDD and BD combined) was investigated using unrelated individuals from two population-based UK cohorts: GS:SFHS (2060 cases and 4495 controls) and UKB (8294 cases and 15,872 controls) (Supplementary Figures 5 and 6, Supplementary Tables 9a). No SNV reached genome-wide significance, but nominal evidence (*p* < 5 × 10^−4^ in GS:SFHS or UKB and *p* < 0.05 in the alternate cohort) was found for association of *GRM5* (chr11q1), *CNTN5* (chr11q2), *PDE4D* (chr5q), *LRRC7* (chr1p) and *VSNL1* (chr2p) (Table 3).

**Table 3:** Association analysis of affective disorder and multiple related traits across the linkage regions in GS and UKB

-LOG(P)	chr1q	chr11q1	chr11q2	chr5q	chr1p	chr2p	chr3q	chr4q	chr16p
GS_GWAS	**3.39** ***CAPN8***	**3.84** ***GRM5***	3.28 *CNTN5*	**3.67** ***PDE4D***	**3.63** ***LRRC7***	3.0 *VSNL1*	**4.18** ***BCHE***	**3.32** ***GLRB***	**4.59** ***PRKCB***
UKB_GWAS	3.18 *TLR5*	2.78 *GRM5*	***3.82 CNTN5***	**3.92** ***PDE4D***	**3.68** ***LRRC7***	**4.05** ***VSNL1***	**4.16** ***EGFEM1P***	**5.2**	**5.09 TNRC6A**
Neuroticism	2.05	1.96	3.08	*2.91*	2.17	2.06	3.10	2.77	3.21
Extroversion	2.45	2.60	2.29	***5.22*** ***PPWD1***	2.73	2.07	2.33	2.22	2.77
Digit symbol coding	2.94	2.21	**3.41** ***CNTN5***	*2.57*	2.35	2.79	2.32	**3.56 RAPGEF2**	3.12
Verbal fluency	2.94	2.24	2.64	*3.10*	2.22	3.04	2.40	2.94	2.75
Vocabulary	3.01	2.43	**3.49** ***CNTN5***	***4.54 PDE4D***	2.83	2.49	2.79	**3.55 RAPGEF2**	2.59
Logical memory	2.22	2.17	2.49	***4.22 RAB3C***	1.92	2.48	2.24	**3.34** ***LINC02233***	**3.84** ***SCNN1B***
g Factor	1.90	2.25	**3.33** ***CNTN5***	**3.31 MAST4**	2.31	3.02	1.83	***3.58 RAPGEF2***	2.81
GHQ total	2.34	**4.03 5’** ***RAB38***	***3.76 CNTN5***	**3.73** ***RNF180***	2.15	3.12	1.80	2.84	3.26
MDD	2.22	3.04	2.03	***3.74 PDE4D***	2.83	2.35	2.79	3.27	**3.93** ***HS3ST4***
MDQ total	2.88	1.26	3.16	3.29	2.52	**3.80 3**’***KCNS3***	2.63	2.62	2.93
SPQ total	2.42	3.13	2.17	**3.91** ***MAST4***	3.04	1.39	2.74	2.91	2.87
Age of onset	3.25	2.44	**3.32** ***CNTN5***	3.28	2.27	2.05	2.64	2.33	2.68
Episode count	2.84	2.17	***3.55 CNTN5***	2.53	3.24	1.99	2.52	1.90	**3.54** ***HS3ST4***
Region (Mb)	223–225	87.5–89	98.5–100.5	58–67	69–71	17.5–18.5	168–172	158–162	21–27
Genotyped SNPs	382	363	585	1511	297	214	698	563	1239

Most significant *p*-value from case–control association analyses of imputed GWAS data in GS and UKB for affective disorder and for MDD and depression-related traits in GS (Supplementary Methods). Bold—*p* < 0.0005, -Log_10_ = 3.3

To investigate the potential pleiotropy of the associated haplotypes, cognitive, personality and mental health-related traits associated with psychiatric disorders were tested for association in GS:SFHS (Supplementary methods: Region-wide association analyses; Table 3; Supplementary Tables 9b; Supplementary Figures 7 and 8). Nominal association (*p* < 5 × 10^-4^) was identified for multiple traits and genes. Of these, *CNTN5* in chr11q2 showed evidence for nominal association with number of episodes of depression (SCID), psychological distress (GHQ-28B) and the cognitive variables: digit symbol coding and Mill Hill vocabulary. Similarly, *PDE4D* in chr5q was nominally associated with MDD, and Mill Hill vocabulary. Multiple cognitive traits were also nominally associated with SNPs in *RAPGEF2* in chr4q (digit symbol coding, Mill Hill vocabulary and the general cognitive factor g).

Although these cross-trait associations are in some cases within the same subregion, as defined by the recombination rates across the region in the 1000 Genome UK population, for example, for affective disorder, cognitive variable and number of episodes in *CNTN5* (Supplementary Figure 7c), or affective disorder, MDD and Mill Hill vocabulary in *PDE4D* (Supplementary Figure 7d), this is not true for affective disorder and psychological distress in *CNTN5* (Supplementary Figure 7c), or affective disorder and MDD in chr4q (Supplementary Figure 8d). These results suggest that variants in the genes within the family-defined haplotypes are associated, at low penetrance, in the UK population and may modulate aspects of phenotype such as number of episodes and cognitive ability.

Evidence of nominal association with cognitive and mental health-related traits were therefore examined in the t(1;11) family. Haplotype carrier status was used to predict global function (GAF) [34], current IQ (Wechsler Abbreviated Scale of Intelligence) [35] and attention/processing speed (Cambridge Neuropsychological Test Automated Battery, CANTAB) [36]. No association was seen with any haplotype and either current IQ or attention/processing speed (*p* > 0.05). However, chr1q, chr11q1 and chr5q, but not the translocation, predicted GAF (*p* < 0.05 adjusting for age and sex (Supplementary Table 10: Association of haplotypes with global function and cognitive variables in the t(1;11) family). A backward selection mixed regression model retained the translocation, chr11q1, chr5q and chr2p in the minimal model for GAF (*p* = 1.2 × 10^−4^). This confirms a contribution of these haplotypes to global function in the family.

The associations detected in the two UK-based cohorts are not significant at the genome-wide level. The nominal associations with affective disorder and related traits suggest that, despite the evidence for a strong individual effects in the family, these were not observed at a population level with the sample sizes tested.

### Common risk variants predict psychiatric disorder in the translocation family

Using the latest publicly available summary statistic from the Psychiatric Genetics Consortium (PGC) GWAS of MDD [37], BD [6] and SCZ [7], we generated polygenic risk scores (PRS) using PLINK v1.09 [38], *p-*value threshold = 1, from the 48 family members (Supplementary Table 5b: Phenotype prediction using PRS). PRS did not predict Model F (BD, rMDD, MDD), Model B (SCZ, BD, rMDD) or Model C (any diagnosis). PRS for both BD and SCZ were, however, associated with psychosis, even when the analysis was restricted to translocation carriers. Translocation status showed conditional association with psychosis after adjusting for the variance explained by the PRS (Supplementary Table 5c: Minus PRS - t1_11). Backward selection of models containing all three PRS, translocation status and all haplotypes retained haplotypes on the derived chromosomes under all four phenotypic models and were the only variables retained in Model B, Model C and Model F (Supplementary Table 5c: Phenotype prediction). PRS were not significantly associated with age of onset in the family, suggesting that there was no evidence of an increased load of common variants modifying the onset of the phenotypes. To test whether there was an accumulation of common risk variants with subsequent generations, ‘generation’ was used to predict PGC PRS. There was no significant association between PGC PRS and generations in the family (PRS-BD *p* = 0.063, PRS-MDD *p* = 0.148, PRS-SCZ *p* = 0.691). These results suggest that common risk variants, particularly those associated with BD and SCZ, may contribute to psychosis within the family, but there is no evidence of accumulation or loss of polygenic risk over generations.

## Discussion

The evidence for high heritability and familial clustering in psychiatric disorders is counter balanced by the limitations of current diagnostic criteria, over-lapping symptomatology and absence of definitive biomarkers, physiology or pathology. Using whole-genome sequencing, we investigated the existence of disease-modifying loci in a large Scottish pedigree in which a balanced t(1;11) translocation predisposes to major psychiatric disorders. The Scottish t(1;11) family is exceptional because of its size, longitudinal clinical follow-up and detailed molecular genetic study. The foundational finding is of a t(1;11) translocation that disrupts three genes: *DISC1*, *DISC2* and *DISC1FP*, alters *DISC1* expression and results in production of abnormal fusion transcripts [39, 40]. Direct disruption of *DISC1* impacts on neurodevelopment, glutamate-signalling, cognitive ability and liability to psychiatric disorder [41–50]. Studies on individuals from the t(1;11) family have identified abnormalities in brain structure particularly white matter integrity [51] and cortical thickness [18, 52], and in brain activation identified through P300 amplitude and latency [53], as well as activation during working memory tasks, and altered glutamate signalling [18]. These functions are congruent with prevailing hypotheses of neurodevelopmental and synaptogenic origins of SCZ and related disorders. However, the penetrance of the t(1;11) is incomplete and the variability in both age of onset and presentation of symptoms remained unexplained.

Comparison of the two-point LOD scores for the translocation and the theoretical maximum LODs across multiple phenotypes indicated that, while the translocation event and its disruption of the breakpoint genes explains the majority of the linkage to SCZ, SCAFF and BD in the family, additional variants predisposing to affective disorder and psychosis may be identified through linkage analyses. Genome-wide multipoint linkage analyses identified four genome-wide significant linkage peaks (LOD > 3.3), spanning 11 Mb and 51 genes, and five peaks with LOD scores > 2, spanning 35.5 Mb and 285 genes. To prioritise variants, we combined information across linkage analyses in the t(1;11) family, brain cis-eQTLs, and association in two UK population-based cohorts and identified consistent, although nominal, evidence of association between variants in *GRM5*, *PDE4D* and *CNTN5*. All three genes have previously been associated with psychiatric or neurodevelopmental disorders (Supplementary Information: *GRM5*, *PDE4D* and *CNTN5*). PDE4D is a direct protein–protein interactor of DISC1 [54, 55], GRM5 modulates glutamatergic signalling, and *CNTN5* is a neurodevelopmental gene implicated in the specification of dendritic arbors [56, 57]. In the family, it is not possible to separate the direct effects of the translocation and the linked loci on the derived chromosomes. However, the identification of these variants suggests that the family may be segregating multiple genetic hits on causal pathways for psychiatric disorders, consistent with the polygenic nature of these complex traits. Studies of the effects of disrupting *DISC1* in cell and animal models show a clear effect of the translocation on risk of psychiatric illness through this direct effect [49]. The genetic effects of variants in the linkage regions identified in this study will require similar validation.

Although we have highlighted these three genes, multiple subregions within the linked haplotypes may contain independent variants that, in sum, are responsible for different aspects of clinical presentation in the family (e.g. *MAST4* in chr5q and *RAPGEF2* in chr4q; Supplementary Figures 7 and 8). Further, we have shown that PRS derived from PGC summary data for BD and SCZ can predict liability to psychosis in the family, suggesting a contribution from additional common variants. Although, it is important to note that all cases of SZ, BD and psychosis carry the translocation, not all individuals who have the translocation have developed psychotic illness. Our analyses suggest that chr5q, chr2p or chr3q, may contribute in part to phenotypic presentation (Supplementary Table 5). However, unlike those on the derived chromosomes (the translocation, chr1q and chr11q1), these are not present in all individuals affected with psychotic illness (Supplementary Table 4). These results are consistent with the association of the BD and SCZ PRS derived from the PGC data with psychosis, which, when fitted with translocation status, again demonstrates association of the translocation on the background of additional risk variants. Indeed, this study provides evidence for the contribution of variants across a wide spectrum of allele frequencies: common (rs72953088, *GRM5*, MAF = 0.06) as well as rare (rs61749255, *CNTN5*, MAF = 0.0014–0.0056) and the family–specific *DISC1*-truncating mutation caused by the translocation.

This study is limited by the number of family members available for study, restricting the power to detect all variants contributing to the phenotype. However, this is counter balanced by the advantage of reduced genetic heterogeneity and the ability to examine multiple copies of ultra-rare variants in a single pedigree. The contribution of a broad spectrum of allele frequencies to psychiatric disorders, as in other complex traits, argues against the present practise of analysing either very rare or common variants in isolation. It suggests that emerging methods that combine both, such as those searching for compound heterozygosity [58], may be beneficial in predicting complex phenotypes and will allow us to test the prediction that a combination of rare disruptive and common polymorphisms explains a measurable fraction of the individual differences in phenotype. Further, that the separate and joint effects of potentially modulating loci and associated genetic variants need to be assessed for biological consequence and phenotypic impact [59, 60]. We do not rule out the influence of differential environmental exposures on psychiatric outcome, but our study clearly indicates that the high density of psychiatric disorder in the t(1;11) family involves a combination of: direct effects of the translocation on the genes located at the breakpoint (*DISC1*, *DISC2*, *DISC1FP*), the “locking together” of familial risk factors, additional unlinked loci and common risk variants.

In conclusion, although GWAS has identified well over 100 statistically robust findings at the population level for psychiatric disorder, a substantial proportion of variance remains unexplained and this is more so at the individual and family level. Family studies have identified rare, high penetrant variants (akin to the t(1;11), but typically large copy number variants), which have provided valuable pointers to biological targets. Between these two genetic mainstays, classical linkage analysis has had less impact, reflecting the failure to identify consistent linkage at the population level. However, as for the complex neurological trait of Hirschsprung’s Disease [61], our study suggests that an oligogenic model best describes the spectrum of psychiatric presentations in the t(1;11) family. We suggest the application of a combined strategy justifies the linkage approach, not in a sib-pair study design, but an extended within-family design [62]. This strategy has the added benefit of identifying, as we have done here with *DISC1*, *CNTN5*, *PDE4D* and *GRM5*, a subset of variants with both statistical support and biological plausibility. Our study suggests that genetic modifiers may play a significant role in psychiatric disorders and that this is now tractable by whole-genome sequencing in families.

## Electronic supplementary material

Supplementary Information

Dataset 1
