# Supplementary material for: DNA sequence-level analyses reveal potential phenotypic modifiers in a large family with psychiatric disorders
Source: Mol Psychiatry. 2018 Jun 7;23(12):2254–65. doi: 10.1038/s41380-018-0087-4 (PMC6294736; doi:10.1038/s41380-018-0087-4)

Supplementary Information: DNA sequence level analyses reveal potential phenotypic modifiers in a large family with psychiatric disorders. Ryan *et al.*

Italicised page numbers for Tables and Figures denote a link to an external supplementary file: F23 WGS Supplementary_Tables_MolPsych.xls.

Supplementary Methods Page

Diagnoses and phenotypic models in the family 3

WGS sequencing and variant calling 3

i) Library construction and sequencing 3

ii) Mapping and SNV calling in GATK Unified Genotyper 3

iii) Data QC 4

iv) Copy Number Variation (CNV) 4

v) Variant validation - VQSR filter 5

Linkage analysis 6

Haplotype phasing 7

Variant annotation 8

UK Population-based cohorts: GS:SFHS & UKB 8

Region-wide association analyses 9

Supplementary Information

Regional correlations 10

Phenotype prediction 10

Two-point linkage analyses 10

Functional annotation of variants under the genome-wide significant linkage peaks 12

GRM5, *PDE4D* and *CNTN5* 13

Supplementary References

15

Supplementary Tables

Supplementary Table 1 GATK summary table 17

Supplementary Table 2 Phenotype models split by translocation status 17

Supplementary Table 3 Genes in linkage regions. a) RefSeq genes and associated GO

terms for experimentally confirmed biological processes, most significant dbGAP mental health/behavioural phenotypes and Ensembl phenotype annotations. b) Associated dbGAP mental health and behaviour traits p < 1 x 10^{-5} *17*

Supplementary Table 4 Summary of haplotype segregation in the t(1;11) family 18

Supplementary Table 5 Phenotype prediction 19

Supplementary Table 6 Two-point LOD scores for the haplotype regions: a) chr1q; b) chr11q1; c) chr11q2; d) chr5q and e) Regions LOD>2 *19*

Supplementary Table 7 Variant Effect Predictor annotation 20

Supplementary Table 8 Functional investigation of top LOD variants within the phenotype-associated haplotypes: a) Missense b) chr1q; c) chr11q1; d) chr11q2; and e) chr5q and f) Regions LOD>2 *22*

Supplementary Table 9 Association summary: a) Affective disorder GS:SFHS & UKB (p<0.001) b) Cognitive and mental health-related traits *22*

Supplementary Table 10 Association of haplotypes with global function and cognitive variables in the t(1;11) family 22

Supplementary Figures

Supplementary Figure 1a-c. Individual sample coverage and minor allele frequencies 23

Supplementary Figure 2 Multipoint linkage analysis of the t(1;11) family. 26

Supplementary Figure 3. Disease-associated haplotypes 32

Supplementary Figure 4. Intra-family regional correlations 37

Supplementary Figure 5a-e. Regional two-point summaries - variants across the LOD ≥ 3 linkage regions 38

Supplementary Figure 6a-e. Regional two-point summaries - variants across the LOD ≥ 2 linkage regions 42

Supplementary Figure 7a-e. Association summaries: a) chr1q; b) chr11q1; c) chr11q2; and d) chr5q. 47

Supplementary Figure 8a-e. Association summaries: a) chr1p; b) chr2p; c) chr3q; d) chr4q; and e) chr16q. 51

Supplementary Methods

Diagnoses and phenotypic models in the family

Psychiatric diagnoses of the t(1;11) family members were established by consensus between two psychiatrists (D.B. and A.W., the latter being blind to karyotype status[1]) in accordance with DSM-IV criteria using the Structured Clinical Interview for DSM-IV (SCID), hospital records and general practitioner records. Psychosis in this context was defined the presence of one or more of the following symptoms: delusions; hallucinations; disorganized speech (e.g., frequent derailment or incoherence); grossly disorganized or catatonic behaviour. Further details on the clinical assessment of these individuals can be found in Thomson *et al.*, 2016[1].

The study was approved by the Multicentre Research Ethics Committee for Scotland (09/MRE00/81). A detailed description of the study was given and written informed consent was obtained from all individuals prior to participation.

WGS sequencing and variant calling

i) Library construction and sequencing

For the first 10 samples, 1 µg of DNA was sheared with the S2 Series Covaris and libraries were prepared with the Illumina TruSeq paired end adapter kit. For the remaining 39 samples, 100 ng aliquots of genomic DNA were fragmented using the S2 Series Covaris (with Illumina recommended settings) and paired end sequencing libraries were prepared using the Illumina TruSeq Nano LT library protocol. The gDNA was sheared to ~250 bp fragments, and size selection was performed using AMPure magnetic beads (Agencourt). The fragments were then end repaired, adenylated, and Illumina adapters were ligated. Ten cycles of PCR enrichment were performed. Library quality was assessed using an Agilent Bioanalyzer HS chip and library quantity was determined using a Qubit dsDNA HS assay kit. Each sample was sequenced over 3 lanes of a HiSeq2000, using a paired end 101bp run. Basecalls were generated using the Illumina Real Time Analysis (RTA) software. The binary files containing sequences and quality scores were transferred to a shared HPCC running Linux for further processing. The Illumina bcl2fastq software was then used to convert the files to Fastq format, and only reads passing the standard Illumina quality filter were included in the output files.

ii) Mapping and SNV calling in GATK Unified Genotyper

Reads were aligned to the human reference genome (UCSC hg19) with the BWA program[2], allowing two mismatches in a 30 base pair seed. SAMtools[3] was used to process the alignments, including sorting, merging, and indexing. Duplicate read pairs (reads with identical outer coordinates) were

removed with Picard (http:// http://broadinstitute.github.io/picard/) to counteract PCR artifacts generated during the sample preparation. Bamtools[4] was used to filter for properly paired reads. Picard CollectMultipleMetrics and GCbias was used to determine insert size and quality stats for each library. SNPs and small indels were called with GATK v2.4.9[5-7]. Local realignment around indels and base quality score recalibration was performed for each library. SNVs were called using the Unified Genotyper in multi-sample joint calling mode in order to achieve consistent calling across samples. GATK Variant Quality Score Recalibration (VQSR) modeling was employed, and only variants passing all metrics were used for downstream analyses.

Variants were checked for Mendelian segregation using Pedcheck in Merlin[8]. Multiallelic variants and those with one or more Mendelian errors were removed from the analysis.

iii) Data QC

Data for each genome was assessed for overall quality and coverage. The percentage of reads properly paired, rate of PCR duplicates, library complexity, mean library insert size, mean coverage and GC bias were examined. Ts/Tv ratios of the SNVs called by Unified Genotyper for each individual and a comparison of SNV calls to dbSNP 129 were determined using GATKVariantEval. A check of sample sex was performed for each sample by comparing heterozygosity on the X chromosome and coverage of the X and Y chromosomes to the reported sex. Males were expected to have few heterozygous sites on chrX and to have similar coverage levels on the X and Y chromosomes, while females were expected to have many heterozygous calls on chrX, higher chrX coverage and little coverage of the Y chromosome.

We performed a kinship analysis in order to assess the degree of relatedness amongst samples and to rule out any sample mislabelling. We used the software KING[9] to compute the coefficient of relatedness from the VCF file using PASS variants.

To confirm the population of samples, PCA analysis was performed using Peddy 0.3.2[10].

iv) Copy Number Variation (CNV)

An adaptation of a CNV detection tool developed by Yoon *et al.*[11] was used to identify CNVs. Each chromosome was split into non-overlapping 100bp windows and depth of reads with mapping quality 20 or greater was calculated within the window using bedtools. Mean and standard deviation of read depth within each individual were computed after filtering windows overlapping with segmental

duplications, gaps, and mappability less than 1. For mappability, the wgEncodeCrgMapabilityAlign100mer table was downloaded from UCSC genome browser.

Using the mean and standard deviation, read depth per window was converted and normalized within each individual as Z-score, named z1-score. To control for batch effects, we used fourteen WGS in-house control samples. The Z-score of each window was standardized against the mean Z-score of the control samples in the same window, named z2-score. The segmentation algorithm in Yoon et al (2009) was applied to the z2-scores. Focusing on deletion calls, raw deletion calls were filtered by size >=1000 bp, z1-score< -1.5 and z2-score < -1.5. Reads are assigned to have mapping quality 0 (q0 reads) when mapped to multiple locations in genome. The deletion regions with high q0 reads (>5%) were filtered out to select calls from uniquely mapped regions.

From filtered deletion regions, we selected the regions that were preferentially shared between affected members of each nuclear family, i.e. within first-degree relatives. The signals of reads with mapping quality 20 or greater of the called regions and flanking regions were visually inspected to remove poor quality and noisy calls. Then IGV plots were next examined to filter by additional evidence (soft clipped reads and read alignment) around the breakpoints. After manual filtering, 27 unique deletion regions are obtained with sizes ranging from 1.2 kb to 145 kb. The intergenic CNV in *CNTN5* was confirmed by PCR amplification and Sanger sequencing. Sanger sequencing was carried out using custom primers and the Big Dye terminator sequencing kit (Life Technologies) on an Applied Biosystems 3730XL DNA sequencer. The deletion breakpoints were determined by aligning the sequences to the hg19 genome using BLAT[12].

v) Variant validation - VQSR filter

Custom designed Taqman assays (Applied Biosystems) were developed to 231 SNVs and genotyped across all 48 DNAs from the t(1;11) family to confirm both the presence of the SNV and correct variant segregation. Of these 116 SNVs had rs#, of which 67 were Pass variants and 49 were non-Pass variants. The global MAF (dbSNP) of rs# variants tested ranged from 0.002 to 0.49, with 3 Pass variants and 10 non-Pass variants having no global MAFs. There was no significant difference in global MAF between Pass and non-Pass variants tested (p=0.67). In total, 180 variants validated (78%) of which 134 were VQSR Pass variants (94% of all Pass variants tested, 134 out of 143 variants) and 46 (52% of all non-Pass variants tested, and 46 out of 88 variants). SNVs that failed to validate included those where the alternate allele was not detected in the family by the Taqman assay (N=36; 3 Pass and 33 non-Pass), and those where the heterozygotes did not match the sequencing data (N=7; 3 Pass and 4 non-Pass).

The identified variants have been deposited in dbSNP (accession numbers ss2137543799 and ss3343271435-ss3353031818).

Linkage analysis

The linkage analysis tool SOLAR (version 8.1.1)[13,14] was used to perform both two-point and multipoint variance component linkage analysis. Minor allele frequencies were taken from the 1000 Genomes Phase 3 for Europeans (EUR) data set[15]. LOD adjustment was performed using the LODadj command in SOLAR[13, 14] to correct deviation of the data from multivariate normality. This command corrects the potential inflation of observed LOD scores by regressing observed LOD scores on simulated LOD scores, generated by a simulation of a normally distributed trait (under a null hypothesis of no linkage) over 10,000 permutations[14,16]. All LOD scores presented are adjusted LOD scores.

Identity by descent matrices (IBDs) and multipoint IBDs were calculated for two-point and multipoint linkage analyses respectively[13]. To maximise CPU usage, the IBD step of SOLAR was parallelised across multiple cores using a custom script.

Two-point linkage analysis was performed on quality control (QC) filtered variants within the boundaries of the haplotypes in the genome-wide significant (LOD ≥ 3.3) multipoint linkage regions on chr1q, chr5q and chr11q (two separate haplotypes: chr11q1 and chr11q2). The variant filter parameters were: Hardy-Weinberg equilibrium (HWE) p> 0.001; Mendelian errors = 0; seen in ≥3 individuals. In addition, due to the size of the region and number of SNPs, the variants in the chr5q were also filtered to exclude variants that did not achieve an OddsRatio (OR) ≥ 1.5 in association with the model F phenotypes. Two-point linkage analysis was also performed on variants under the nominally significant multipoint linkage peaks (LODs ≥ 2) on chr1p, chr2p, chr3q, chr4q and chr16. These variants were also selected based on the QC parameters described above, with the variants under the chr1p, 3q, 4q and chr16p peaks being further restricted to those that could not be imputed using the 1000 Genomes Phase 3 reference panel. This was again due to the large number of SNPs in these regions.

We calculated the maximum theoretical two-point LODs (mtLODs) for the pedigree, drawn from the 48 whole genome-sequenced individuals for each phenotypic model. These are presented in Table 1. These LODs represent the highest LOD scores achievable in this pedigree assuming perfect segregation of a phenotype-associated allele with a MAF of 0.00001 (the lowest value accepted by SOLAR), showing the maximum power in the family for each phenotype model.

For the multipoint analyses, random sets of variants were selected across the autosomal chromosomes (chr1-12 separated into p and q arms) from the GATK PASS filtered variants from the t(1;11) family WGS data. These variants were chosen using the following additional QC parameters: an observed heterozygosity >0.2 and <1; no Mendelian errors; Hardy-Weinberg equilibrium (HWE) p>0.001; and present on at least 2 chromosomes. Three independent datasets were selected for multipoint analysis and combined to give an approximate density of three variants per mega-base (Mb).

Haplotype phasing

Regions of the genome with genome-wide significant or suggestive multipoint LOD scores (LOD≥3.3 and LOD≥2 respectively) were phased and the linked haplotypes identified. The genomic regions under the linkage peaks were phased using the software SHAPEIT (v2.r837) and the 1000 Genomes Phase 1 integrated reference panel (b37 NCBI build, release June 2014: ALL.integrated_phase1_SHAPEIT_16-06-14.nosing.tgz).

Disease-linked haplotypes were defined by identifying the minimum region of homology shared by the maximum number of affected individuals around the top LOD variants identified. The haplotype boundaries were defined by the first recombination breakpoint in an affected individual at each end of the region.

Variant annotation

The genes that lie under the multipoint linkage peaks were identified using the UCSC genome browser (GRCh37/hg19 assembly) *refGene* MySQL table (from genome-mysql.cse.ucsc.edu). Gene names were limited those approved by HUGO. Missense variants were assessed using VEP. Variants were assessed for their potential to alter gene expression using two approaches: Regulome DB[17], which predicts regulatory elements in non-coding regions of the genome; and comparison to BRAINEAC[18], a brain specific eQTL database. A LOD cut-off of 0.5 x mtLOD for the best model for each linkage region was used to filter variants; only variants above this threshold being taken forward for functional investigation.

Regulome DB (http://www.regulomedb.org/index) scores SNPs using a scoring system that represents the likelihood of a variant having a functional impact on gene expression. A description of the supporting data associated with each Regulome DB score is included in Supplementary Table 8 (See F23 WGS Supplementary_Tables_MolPsych.xls).

The BRAINEAC database contains cis-eQTL data generated across 10 different brain regions in 134 neuropathologically normal control individuals (http://www.braineac.org/). Where a variant was not present in BRAINEAC, variants with r^2=1 were identified from the 1000 Genomes Project phase 1 using SNAP (https://archive.broadinstitute.org/mpg/snap/ldsearch.php) and these proxy SNPs were queried in BRAINEAC. Supplementary Table 8 (See F23 WGS Supplementary_Tables_MolPsych.xls) reports all SNPs which are significant cis- eQTLs (p-value < 0.05; either directly or via a proxy SNP), the genes with significantly altered expression levels, as well as the most significant gene(s) and its corresponding p-value.

UK Population-based cohorts: GS:SFHS & UKB

GS:SFHS genotyping, imputation and quality control is detailed in a previous publication[19]. The following were removed from the sample: individuals with discrepancies of sex, duplicate samples, and individuals of suspected Italian descent. SNPs with info < 0.3, minor allele frequency < 0.5% or a Hardy-Weinberg Equilibrium test $p<10^{-6}$ were excluded. After initial quality control, the data set consisted of 19,994 individuals with multiple degrees of kinship genotyped for 8,967,714 SNVs. Individuals were retained based on relatedness, maximizing retention of case individuals, using PLINK (pi-hat < 0.05). A sample of 6,555 individuals for 186,982 imputed SNPs in the linkage regions remained for further analysis. Quality control and association analyses of GS:SFHS and clinical samples were performed using Plink 1.9[20] (www.cog-genomics.org/plink/1.9/).

Details of sample processing specific to the UK Biobank project and the Axiom array are available from the following links respectively: http://biobank.ctsu.ox.ac.uk/crystal/refer.cgi?id=155583; http://media.affymetrix.com/support/downloads/manuals/axiom_2_assay_auto_workflow_user_guide.pdf. UK Biobank genotyping and the stringent QC protocol applied to UKB data before it was released can be found at (http://biobank.ctsu.ox.ac.uk/crystal/refer.cgi?id=155580). This research has been conducted using the UK Biobank Resource under UK Biobank main application 4844.

We applied the following additional quality control steps: individuals were removed based on UKB genomic analysis exclusion (UKB Data Dictionary item #22010), non-white British ancestry (#22006: genetic ethnic grouping; from those individuals who self-identified as British, principal components analysis was used to remove outliers), high missingness (#22005), genetic relatedness (#22012; no pair of individuals have a KING-estimated kinship coefficient > 0.0442), QC failure in UK BiLEVE study (#22050 and #22051: UK BiLEVE Affymetrix and UK BiLEVE genotype quality controls for samples) and

gender mismatch (#22001: genetic sex), MAF ≥ 0.1%. Furthermore, individuals in UKB who were also participants in GS:SFHS were excluded to remove any chance of overlap of individuals between the two UK samples. A final sample of 24,166 individuals with 11,157,897 imputed SNVs remained for further analysis including 235,798 SNPs in the linkage regions.

The frequency of the phenotype-linked haplotypes in the Scottish population was calculated in Haploview[21] using the genotype data from GS:SFHS.

Region-wide association analyses

Affective disorder was defined as a diagnosis of MDD or BD in GS:SFHS[22] or probable BD or MDD in UKB[23]. Further phenotypes used in GS:SFHS are described in [22,24].

For association analyses, sample specific covariates were applied as follows:

GS:SFHS. Imputed data was analysed using ProbAbel[25]. All analyses were adjusted for age, sex and 6 PCs to account for population stratification.

UKB. Imputed data was analysed using SNPTest[26]. All analyses were adjusted for centre, array and batch as random effects, and age, sex and 15 informative principal components as fixed effects (PCs; UKB Data Dictionary items #22009.01 to #22009.15) to take account of batch effects and possible population stratification.

Additional mood and cognitive measures included the General health questionnaire 28 (GHQ28)[27], the mood disorder questionnaire (MDQ)[28] and schizotypal personality (SPQ-B)[29] questionnaire. The personality traits of neuroticism and extraversion were assessed using the Eysenck personality questionnaire-revised short form.[30] Cognitive tests applied in GS:SFHS were Logical Memory immediate and delayed scores from the Wechsler Memory Scale III[31], Digit Symbol-Coding from the Wechsler Adult Intelligence Scale III[32], phonemic verbal fluency using the letters C, F and L[33], and Mill Hill Vocabulary (combined junior and senior synonyms)[34].

Potential pleiotropy of 5,853 genotyped SNPs in the regions was examined in GS:SFHS using Plink 1.9[20]. All analyses were adjusted for age, sex and 6 PCs to account for population stratification. The reported p-values are empirical p-values were derived using adaptive permutation. Nominal significance was assessed at the threshold $p < 5x10^{-4}$, consistent with haplotype permutation analyses and with the assessment of 1.5% of the euchromatic genome at a genome-wide nominal significance p-value of $1x10^{-5}$ (0.00001*1/0.015).

Supplementary Information

Regional correlations

As expected, there are very high correlations between the translocation and the chr1q and chr11q1 haplotypes (Spearman's rho > 0.88) (Supplementary Figure 4: Intra-family regional correlations). These three regions showed lower, but strong correlations with the chr11q2 haplotype (rho = 0.78, 0.71 and 0.67, respectively; all p<0.01). Moderate correlations were also detected between the chr1, chr11q1 and chr11q2 haplotypes and the chr5q haplotype (rho = 0.40, 0.46 and 0.32, respectively; all p<0.05); although the correlation between the translocation and the chromosome 5q haplotype was lower (rho = 0.29, p<0.05). Similarly, a moderate correlation (rho = 0.43 - 0.45; all p<0.01) was observed between the chr2p and chr16p haplotypes and the chr1q haplotype. Moderate correlations were also seen between chr4q and the chr5 and chr1p haplotypes (rho = 0.48 and 0.49, respectively; all p<0.01).

Phenotype prediction

Under a backward selection logistic mixed regression model, only haplotype chr11q1 is retained for Model C: any psychiatric diagnosis (final model $p=1.4x10^{-11}$). For narrower models, the contribution of the additional chromosomes reflects their model specific multipoint LODs, with chr11q2 contributing to the affective disorder phenotype (BD, rMDD, MDD; Model F; final model $p=1.1x10^{-7}$); and the translocation, chr1q, chr2p, chr3q and chr5q being retained for Psychosis (final model $p=1.7x10^{-9}$). Analyses of age of onset for individuals with a diagnosis of affective disorder retained both chr11q2 and chr16p in the minimal model (p=0.018). These results suggest that in addition to the translocation and associated haplotypes (chr1q, chr11q1 and chr11q2), the t(1;11) family carries additional risk haplotypes that increase the risk of Psychosis (chr5q, chr2p and chr3q) and affective disorder (chr11q2) and may modify the age of onset of illness (chr11q2 and chr16p). Supplementary Table 5.

Two-point linkage analyses

Supplementary Figure 5a shows the two-point LODs across the chr1q haplotype. Within this region there appear to be multiple blocks with strong linkage to Model B (SCZ, SCZAFF, rMDD and cyclothymia) and a smaller region showing maximum linkage to Model C (any psychiatric diagnosis).

The most significant block of linkage appears to span the first five exons and the proximal intergenic region 5' of *CAPN8* (up to ~20kb from the TSS). The variant with the highest two-point LOD score within the chr1q multipoint linkage region, chr1:223804982 (LOD 9.3, Model C; mtLOD: 12.0), is located within this linkage block. This variant is located within intron 9 of *CAPN8*. A second block, that shows strong linkage to Model C, spans the promoter, 5'UTR and first exon of *SUSD4* and the gene *CCDC18* (mLOD 7.8).

A similar pattern of two-point LODs can be seen for the chr11q1 haplotype. Again, it is worth noting the complexity of the haplotype region, with blocks of linkage maximally-associated with multiple models (Supplementary Figure 5b). The most consistent linkage block covers an intergenic region upstream of *GRM5* (~56-6.5 Kb away from the TSS) which is highly linked in Model C. This region also contains the variant with the highest two-point LOD for this haplotype, chr11:88219524 (10.8 LOD, Model C). This variant is intergenic, located between *CTSC* and *GRM5*. The second highest two-point LOD score (10.3, model C) is for rs12275439, located in the third intron of *GRM5*.

Two-point analysis of the chr11q2 haplotype (Supplementary Figure 5c) region shows the strongest linkage to Model F, with a discrete signal in *CNTN5* containing three SNPs. The highest two-point LOD for this region was 8.3 (Model F; mtLOD: 8.9) for chr11:99653622, located in an intron of *CNTN5*). Inspection of the phased sequencing data revealed that three further individuals who contribute to the top two-point LODs for Model F in this region, IDs 44,47,and 87 (Table 4).

The chr5q haplotype appears to have two Model F defined sub-regions: one upstream of *PDE4D* and a second downstream of *ZSWIM6* (Supplementary Figure 5d). The variant with the best two-point LOD score for the chr5q haplotype was rs377870 (LOD 5.1, Model F). This variant is intergenic and located between *ZSWIM6* and *KIF2A*. The block of linkage around the promoter of *PDE4*D contains multiple high LOD variants for Model F including rs201533757 which generated the second highest LOD for the region (LOD 4.9, Model F) and is intergenic between *PDE4D*/*PART1* and *DEPDC1B*.

Similar patterns can be seen for the limited two-point analyses in the regions with LOD> 2 (Supplementary figure 6a-e).

Full results from the two-point linkage analyses are given in Supplementary Table 6 Two-point LOD scores for the haplotype regions containing tables for the top 100 SNV for each region and for all variants tested

Functional annotation of variants under the genome-wide significant linkage peaks

The four genome-wide significant linkage regions contain 21 amino acid altering variants shared by all individuals contributing to the LOD scores: 14 on chr1q, five on chromosome 11q1 and one on chr11q2 and chr5q. We used Ensembl's Variant Effect Predictor (VEP) tool (McLaren et al., 2016) to annotate the predicted consequences of these non-synonymous substitutions (Supplementary Tables 7: Variant Effect Predictor annotation). In chr1q1, probable damaging variants were detected in *TLR5*, *CAPN2,* and *NVL*, and additional variants in *CCDC185*, *CAPN8* and *TP53BP2* are predicted to be damaging or possibly damaging by Polyphen. In chr11q1 and chr11q2, missense variants in *CTSC* and *CNTN5* respectively, were predicted to be deleterious by SIFT, but not by PolyPhen. The chr5q haplotype contains one missense variant, rs150642131, in *CWC27*; however SIFT and PolyPhen suggest that this substitution is tolerated/benign. Of the 21 missense variants, 12 variants have evidence of being brain cis-eQTLs, including: rs17599, rs35539373 and rs71644745 that were associated with expression of *CAPN2*, *WDR26*, *CCDC185*, and *NVL;* while rs217086 was associated with *GRM5* expression (Supplementary Tables 8a: Functional investigation of top LOD variants within the phenotype-associated haplotypes).

To assess the effects of non-coding variants within the phenotype-associated haplotypes, 340 variants that achieved a LOD of greater than half the maximum theoretical LOD were assessed for potential functional effects in Encode data from RegulomeDB[17] and brain cis-eQTL data from Braineac[18]. For variants not present in Braineac, proxy SNPs with an $r^2 = 1$ were identified in the European panel of the 1000 Genomes phase 1 using the online tool SNAP[35]. Twelve SNPs were likely to affect binding of transcription factors (RegulomeDB score 2c or above) and 184 SNPs were significantly associated with gene expression in at least one brain region ($p<0.05$, Supplementary Tables 8: Functional investigation of top LOD variants within the phenotype-associated haplotypes). The strongest evidence for a cis-eQTL in chr1q is for rs75866472 (intronic SNP in *CNIH3*) which is associated with altered expression of *ENAH* in the hippocampus ($p = 2.6 \times 10^{-6}$). In chr11q1, only a single variant, rs72953088 (intronic SNP in *GRM5*), is likely to affect protein binding, disrupting a CTCF motif. This SNP was not present in Braineac, but the proxy SNP rs4533040 is predicted to alter the expression of multiple genes including *GRM5*. The top cis-eQTL in chr11q1 was rs10128749, which was associated with the expression of *GRM5* in the medulla. No SNPs in chr11q2 gave evidence for altered protein binding; however, the most significant eQTL, rs76452538 (an intronic SNP in *CNTN5*), was associated with expression changes in *FLJ32810, TMEM133, ARHGAP42* in the temporal cortex ($p = 0.00085$) and *CNTN5* ($p = 0.036$) in the white matter tracts. Neither of the two SNPs with the highest LOD in the chr5q region gave evidence of protein binding; however rs377870, a SNP in the intron of *C5orf64*, is associated with the expression of *ERCC8* in the temporal cortex. Two SNVs in chr5q with LOD > 3.3 are associated with

altered expression of *PDE4D*. Multiple high LOD SNPs lie in the promoter region of *PDE4D* (Supplementary Table 6d), of which rs988827 and rs11957508 are predicted to be cis-eQTLs for *PDE4D* (rs988827, p = 0.0013 in the occipital cortex and p = 0.037 averaged over all regions).

Two of the 27 deletion variants detected in the family are located within the linkage regions: an intergenic deletion, chr4:163,393,600-163,397,100 (MAF = 0.50 1000G phase v3, DGV id: esv3602938) and chr11:99185458-99186698, an intronic deletion in *CNTN5* (MAF = 0.11 1000G phase v3, DGV id: esv2661704). Multiple SNPs in the *CNTN5* deleted intronic region (hg19 snp147) are associated with altered *CNTN5* expression, with the best evidence being for rs56316662 ($p = 5 \times 10^{-4}$ in the substantia nigra).

Functional annotation of SNV with LODs> 2 are given in Supplementary Table 6e and Supplementary Table 8f. Within the chr4q deletion region multiple SNV are associated with the expression of genes in the region, most prominently rs6822066, shown to affect the expression of *FSTL5*, *NPY1R*, *TKTL2*, *NAF1*, and *NPY5R* (best p = 0.00077 for *FSTL5* in the thalamus).

GRM5, *PDE4D* and *CNTN5*

GRM5 maps within the chr11q1 haplotype, close to the translocation breakpoint[36], and encodes a glutamate receptor previously associated with psychiatric disorders[37-39]. This protein is involved in the regulation of neural network activity and synaptic plasticity. Glutamatergic neurotransmission is involved in most aspects of normal brain function and can be perturbed in many neuropathological conditions. Multiple non-coding variants in the intron and downstream region of *GRM5* were predicted to alter expression of *GRM5* and other genes in the region in multiple brain regions. These included rs72953088 (MAF – 0.04; LOD 7.3 SCZ, BP, rMDD and cyclothymia; Model B) a SNP predicted to disrupt a CTCF binding site, CTCF is transcriptional regulator that binds chromatin insulators and prevents interaction between promoter and nearby enhancers and silencers. This SNP is associated with altered expression of both *GRM5* and other genes in the region. In clinical brain imaging studies of the family, t(1;11) carriers show abnormal glutamate signalling[1] probably in part through the direct disruption of *DISC1*[40,41]. The identification of potentially functional variants associated with disrupted expression of a glutamate receptor may indicate a double-hit on function of this excitatory neurotransmitter.

PDE4D maps within the chr5q haplotype and encodes a cAMP-specific 3',5'-cyclic phosphodiesterase and degrades cAMP, which acts as a signal transduction molecule. This protein is known to interact with DISC1, the protein product of the gene directly disrupted by the translocation, and may indicate

a further hit on the same neurodevelopmental signalling pathway. Association analysis in the Scottish population suggest that variants in this gene are associated with MDD and Mill Hill vocabulary a test of crystallised cognitive ability thought to reflect premorbid IQ.

CNTN5 is the only gene in the chr11q2 haplotype, and its protein is involved in the formation of axon connections in the developing nervous system. *CNTN5*, and the closely related genes *CNTN4* and *CNTN6*, have been associated with neurodevelopmental disorders and psychiatric disorders particularly autism[42,43] and most recently Tourette Syndrome[44]. It has recently been implicated in the patterning of dendritic arborisation[45]. We identified multiple potential risk variants in *CNTN5*: a CNV; multiple SNPs within the introns; and rs61749255, a non-synonymous amino acid substitution in exon 6 (MAF - 0.0056; LOD 4.5 SCZ, BP, rMDD and cyclothymia). Association analysis in the Scottish population suggested that variants in this gene impact on the number of episodes, symptoms of psychological distress and cognitive traits.

Supplementary References

1. Thomson, P.A. *et al.* Balanced translocation linked to psychiatric disorder, glutamate, and cortical structure/function. *NPJ Schizophr* **2**, 16024 (2016).
2. Li, H. & Durbin, R. Fast and accurate short read alignment with Burrows-Wheeler transform. *Bioinformatics* **25**, 1754-60 (2009).
3. Li, H. *et al.* The Sequence Alignment/Map format and SAMtools. *Bioinformatics* **25**, 2078-9 (2009).
4. Barnett, D.W., Garrison, E.K., Quinlan, A.R., Stromberg, M.P. & Marth, G.T. BamTools: a C++ API and toolkit for analyzing and managing BAM files. *Bioinformatics* **27**, 1691-2 (2011).
5. McKenna, A. *et al.* The Genome Analysis Toolkit: a MapReduce framework for analyzing next-generation DNA sequencing data. *Genome Res* **20**, 1297-303 (2010).
6. Van der Auwera, G.A. *et al.* From FastQ data to high confidence variant calls: the Genome Analysis Toolkit best practices pipeline. *Curr Protoc Bioinformatics* **43**, 11 10 1-33 (2013).
7. DePristo, M.A. *et al.* A framework for variation discovery and genotyping using next-generation DNA sequencing data. *Nat Genet* **43**, 491-8 (2011).
8. Abecasis, G.R., Cherny, S.S., Cookson, W.O. & Cardon, L.R. Merlin--rapid analysis of dense genetic maps using sparse gene flow trees. *Nat Genet* **30**, 97-101 (2002).
9. Manichaikul, A. *et al.* Robust relationship inference in genome-wide association studies. *Bioinformatics* **26**, 2867-73 (2010).
10. Pedersen, B.S. & Quinlan, A.R. Who's Who? Detecting and Resolving Sample Anomalies in Human DNA Sequencing Studies with Peddy. *Am J Hum Genet* **100**, 406-413 (2017).
11. Yoon, S., Xuan, Z., Makarov, V., Ye, K. & Sebat, J. Sensitive and accurate detection of copy number variants using read depth of coverage. *Genome Res* **19**, 1586-92 (2009).
12. Kent, W.J. BLAT--the BLAST-like alignment tool. *Genome Res* **12**, 656-64 (2002).
13. Almasy, L. & Blangero, J. Multipoint quantitative-trait linkage analysis in general pedigrees. *Am J Hum Genet* **62**, 1198-211 (1998).
14. Goring, H.H., Williams, J.T. & Blangero, J. Linkage analysis of quantitative traits in randomly ascertained pedigrees: comparison of penetrance-based and variance component analysis. *Genet Epidemiol* **21 Suppl 1**, S783-8 (2001).
15. Genomes Project, C. *et al.* A global reference for human genetic variation. *Nature* **526**, 68-74 (2015).
16. Blangero, J., Williams, J.T. & Almasy, L. Robust LOD scores for variance component-based linkage analysis. *Genet Epidemiol* **19 Suppl 1**, S8-14 (2000).
17. Boyle, A.P. *et al.* Annotation of functional variation in personal genomes using RegulomeDB. *Genome Res* **22**, 1790-7 (2012).
18. Ramasamy, A. *et al.* Genetic variability in the regulation of gene expression in ten regions of the human brain. *Nat Neurosci* **17**, 1418-28 (2014).
19. Kerr, S.M. *et al.* Pedigree and genotyping quality analyses of over 10,000 DNA samples from the Generation Scotland: Scottish Family Health Study. *BMC Med Genet* **14**, 38 (2013).
20. Chang, C.C. *et al.* Second-generation PLINK: rising to the challenge of larger and richer datasets. *Gigascience* **4**, 7 (2015).
21. Barrett, J.C., Fry, B., Maller, J. & Daly, M.J. Haploview: analysis and visualization of LD and haplotype maps. *Bioinformatics* **21**, 263-5 (2005).
22. Smith, B.H. *et al.* Cohort Profile: Generation Scotland: Scottish Family Health Study (GS:SFHS). The study, its participants and their potential for genetic research on health and illness. *Int J Epidemiol* **42**, 689-700 (2013).
23. Smith, D.J. *et al.* Prevalence and characteristics of probable major depression and bipolar disorder within UK biobank: cross-sectional study of 172,751 participants. *PLoS One* **8**, e75362 (2013).

24. Smith, B.H. *et al.* Generation Scotland: the Scottish Family Health Study; a new resource for researching genes and heritability. *BMC Med Genet* **7**, 74 (2006).
25. Aulchenko, Y.S., Struchalin, M.V. & van Duijn, C.M. ProbABEL package for genome-wide association analysis of imputed data. *BMC Bioinformatics* **11**, 134 (2010).
26. Marchini, J., Howie, B., Myers, S., McVean, G. & Donnelly, P. A new multipoint method for genome-wide association studies by imputation of genotypes. *Nat Genet* **39**, 906-13 (2007).
27. Goldberg, D.P. & Hillier, V.F. A scaled version of the General Health Questionnaire. *Psychological medicine* **9**, 139-145 (1979).
28. Hirschfeld, R.M. *et al.* Development and validation of a screening instrument for bipolar spectrum disorder: the Mood Disorder Questionnaire. *American Journal of Psychiatry* **157**, 1873-1875 (2000).
29. Raine, A. & Benishay, D. The SPQ-B: A brief screening instrument for schizotypal personality disorder. *Journal of personality disorders* **9**, 346-355 (1995).
30. Eysenck, S.B., Eysenck, H.J. & Barrett, P. A revised version of the psychoticism scale. *Personality and individual differences* **6**, 21-29 (1985).
31. Wechsler, D. WMS-IIIUK administration and scoring manual. (Psychological Corporation, London, UK, 1998).
32. Wechsler, D. *Manual for the Wechsler Adult Intelligence Scale-III*, (1981).
33. Lezak, M. Neuropsychological Testing. (Oxford University Press, Oxford, 1995).
34. Raven, J.C. & Court, J.H. *Raven's progressive matrices and vocabulary scales*, (Oxford Psychologists Press, 1998).
35. Johnson, A.D. *et al.* SNAP: a web-based tool for identification and annotation of proxy SNPs using HapMap. *Bioinformatics* **24**, 2938-9 (2008).
36. Semple, C.A., Devon, R.S., Le Hellard, S. & Porteous, D.J. Identification of genes from a schizophrenia-linked translocation breakpoint region. *Genomics* **73**, 123-6 (2001).
37. Devon, R.S. *et al.* The genomic organisation of the metabotropic glutamate receptor subtype 5 gene, and its association with schizophrenia. *Mol Psychiatry* **6**, 311-4 (2001).
38. Matosin, N., Fernandez-Enright, F., Lum, J.S. & Newell, K.A. Shifting towards a model of mGluR5 dysregulation in schizophrenia: Consequences for future schizophrenia treatment. *Neuropharmacology* **115**, 73-91 (2017).
39. Matosin, N. *et al.* Effects of common GRM5 genetic variants on cognition, hippocampal volume and mGluR5 protein levels in schizophrenia. *Brain Imaging Behav* (2017).
40. Dawson, N. *et al.* Altered functional brain network connectivity and glutamate system function in transgenic mice expressing truncated Disrupted-in-Schizophrenia 1. *Transl Psychiatry* **5**, e569 (2015).
41. Brandon, N.J. *et al.* Understanding the role of DISC1 in psychiatric disease and during normal development. *J Neurosci* **29**, 12768-75 (2009).
42. Oguro-Ando, A., Zuko, A., Kleijer, K.T.E. & Burbach, J.P.H. A current view on contactin-4, -5, and -6: Implications in neurodevelopmental disorders. *Mol Cell Neurosci* **81**, 72-83 (2017).
43. Zuko, A. *et al.* Contactins in the neurobiology of autism. *Eur J Pharmacol* **719**, 63-74 (2013).
44. Huang, A.Y. *et al.* Rare Copy Number Variants in NRXN1 and CNTN6 Increase Risk for Tourette Syndrome. *Neuron* **94**, 1101-1111 e7 (2017).
45. Peng, Y.R. *et al.* Satb1 Regulates Contactin 5 to Pattern Dendrites of a Mammalian Retinal Ganglion Cell. *Neuron* **95**, 869-883 e6 (2017).

Supplementary Tables

Supplementary Table 1 GATK summary table

Number of Samples	49		
Total Number of Variants	10,894,387		
GATK Filter			
PASS	7,953,978	73.01%	
LowQual	209,547	1.92%	
VQSRTrancheSNP99.00to99.90	2,033,354	18.66%	
VQSRTrancheSNP99.90to100.00	697,508	6.40%	
	ts	tv	ts/tv
All	6,402,693	3,269,113	1.96
Coding	40,228	14,984	2.68
PASS	4,963,283	2,308,215	2.15
PASS & Coding	34,053	11,282	3.02

Supplementary Table 2 Phenotype models split by translocation status

Summary data for t(1;11) family, showing the diagnostic categories included in each phenotype model; the number of cases within each model, further split into those who carry the translocation (T) and those who do not (NT); the maximum theoretical LOD for each model (mtLOD); and t(1;11) - the LOD achieved by the translocation itself.

Model	Diagnoses	T	NT	ALL	mtLOD	t(1;11)
MODEL A	SCZ, BD, SCZAFF	6	0	6	3.2	2.8
MODEL B	SCZ, BD, SCZAFF, rMDD, cyclothymia	13	2	15	8.6	6.7
MODEL C	SCZ, BD, SCZAFF, rMDD, cyclothymia, MDD, adolescent conduct disorder, generalised anxiety disorder, alcoholism	18	6	24	12.0	5.8
MODEL D	MDD, adolescent conduct disorder, generalised anxiety disorder, alcoholism	5	4	9	5.8	1.3
MODEL E	SCZ, BD, SCZAFF, rMDD	10	2	12	6.9	4.8
MODEL F	BD, rMDD, MDD	9	5	14	8.9	4.2
MODEL G	BD, rMDD	6	2	8	5.7	3.1
MODEL H	SCZ, SCZAFF	4	0	4	2.4	2.2
Psychosis	Psychosis any diagnosis	8	0	8	4.4	1.9
Unaffected		1	22	23		
Unknown		0	1	1		
**Total**		**19**	**29**	**48**		

Supplementary Table 3 Genes in linkage regions

a) RefSeq genes within the boundaries of the linkage haplotypes and associated GO terms for experimentally confirmed biological processes, most significant dbGAP mental health/behavioural phenotypes and Ensembl phenotype annotations. b) Associated dbGAP mental health and behaviour traits $p < 1 \times 10^{-5}$.

See F23 WGS Supplementary_Tables_MolPsych.xls

Supplementary Table 4 Summary of haplotype segregation in the t(1;11) family

Distribution of haplotypes across the sequenced t(1;11) family members. family IDs; diagnostic classes (1 = affected); t(1;11) - translocation status (1 2 = carrier; 1 1 = non carrier) and individuals haplotypes carriers (light blue = familial haplotype carrier sharing the entire haplotype region , and *CNTN5* CNV carriers (dark blue).

Id	Any	Psychosis	SCZ BP	SCZ BP rMDD Cyclo	BP rMDD MDD	BP rMDD	t(1;11)	chr1 q	chr11q1	chr11q2	chr5q	chr1p	chr2p	chr3q	chr4q	chr16p	*CNTN5* CNV
70	1	1	1	1			1 2	1	1	1			1				1
13	1	1		1	1	1	1 2	1	1	1			1			1	1
19	1	1		1	1	1	1 2	1	1	1			1			1	1
18	1	1	1	1			1 2	1	1	1			1				
67	1	1	1	1	1	1	1 2	1	1	1			1	1		1	1
15	1	1	1	1			1 2	1	1				1			1	
49	1	1	1	1			1 2	1	1	1	1			1		1	
41	1	1	1	1	1	1	1 2	1	1	1	1	1		1	1	1	
61	1			1	1	1	1 2	1	1	1	1	1			1	1	
50	1				1		1 2	1	1	1	1	1		1	1	1	
24	1			1	1	1	1 2	1	1	1	1					1	
27	1				1		1 2	1	1	1	1					1	
26	1				1		1 2	1	1	1	1						
32	1			1			1 2	1	1	1	1						
55	1			1			1 2	1	1	1				1	1	1	
9	1						1 2	1	1	1							
104	1						1 2	1	1		1						
53	1			1			1 2	1	1					1		1	
44	1			1	1	1	1 1	1	1		1	1		1	1	1	
87	1				1		1 1	1	1		1	1		1		1	
47	1				1		1 1		1		1	1			1		
54	1			1	1	1	1 1			1	1				1	1	
62	1						1 1									1	
85	1				1		1 1										
35							1 2	1	1	1	1				1	1	
107							1 1			1						1	
29							1 1				1					1	
96							1 1				1				1		
97							1 1				1						
40							1 1					1		1			
52							1 1					1					
80							1 1					1					
88							1 1					1			1		
28							1 1									1	
106							1 1									1	
89							1 1							1		1	
30							1 1										
51							1 1										
82							1 1										
94							1 1							1			1
100							1 1										
25							1 1										
78							1 1										
91							1 1										
92							1 1							1			
99							1 1										
101							1 1										
105							1 1										

Supplementary Table 5 Phenotype prediction

a.

Haplotype	Phenotype	Model B	Model C	Model F	Psychosis	AOO-F
Individual variable p-value	t1_11	**$7.4x10^{-5}$**	**$9.4x10^{-5}$**	**$7.4x10^{-4}$**	0.658	0.143
	chr1q	**$8.3x10^{-5}$**	**$3.9x10^{-5}$**	**$2.1x10^{-4}$**	0.674	0.180
	chr11q1	**$8.3x10^{-5}$**	**$2.4x10^{-5}$**	**$1.3x10^{-4}$**	0.682	0.082
	chr11q2	**$1.5x10^{-4}$**	**$9.1x10^{-5}$**	**$5.4x10^{-4}$**	**0.016**	**0.015**
	chr5q	0.071	**0.014**	**0.006**	0.899	0.123
	chr1p	0.900	0.690	0.230	0.752	0.226
	chr2p	0.807	0.817	0.848	**0.011**	0.119
	chr3q	0.176	0.245	0.277	0.436	0.261
	chr4q	0.117	0.135	0.085	0.666	0.082
	chr16p	**0.002**	**0.003**	**0.002**	0.081	**0.040**
Full Model	ALL-chr	**$3.3x10^{-7}$**	**$4.5x10^{-6}$**	**$2.7x10^{-4}$**	**$4.0x10^{-7}$**	0.473
T-carriers	chr5q-16p	0.171	0.053	**$3.4x10^{-4}$**	**$5.4x10^{-3}$**	0.396

b.

Haplotype	Phenotype	Model B	Model C	Model F	Psychosis	AOO-F
Individual variable p-value	PRS-MDD	0.597	0.389	0.768	0.659	0.197
	PRS-BD	0.056	0.056	0.102	**0.039**	0.441
	PRS-SCZ	0.118	0.101	0.120	**0.038**	1
Full Model	ALL-PRS	0.203	0.136	0.409	**$3.0x10^{-3}$**	0.339
T-carriers	Tonly ALL-PRS	0.533	0.302	0.494	**$7.8x10^{-4}$**	0.717

c.

Haplotype /Score	Phenotype	Model B	Model C	Model F	Psychosis	AOO-F
Adjusted Models	Minus ALL-chr - ALL-PRS	0.796	0.746	0.741	0.536	0.990
	Minus PRS - ALL-chr	**$1.0x10^{-4}$**	**$1.9x10^{-4}$**	**$1.0x10^{-3}$**	**$5.5x10^{-4}$**	0.803
	Minus PRS - t1_11	**$4.9x10^{-6}$**	**$1.1x10^{-5}$**	**$8.8x10^{-4}$**	**$2.9x10^{-3}$**	0.513
Combined Models	ALL + PRS	**$7.1x10^{-6}$**	**$4.5x10^{-5}$**	**$1.9x10^{-3}$**	**$3.0x10^{-6}$**	0.789
	Final Model p-value	**$5.8x10^{-11}$**	**$1.4x10^{-11}$**	**$1.1x10^{-7}$**	**$1.7x10^{-9}$**	**0.018**
	Variable remaining in the Final model	t1_11 chr1q chr11q2	chr11q1	t1_11 chr11q1 chr11q2	t1_11 chr1q chr5q chr2p chr3q	chr11q2 chr16p

P-values of mixed linear regression models: a) using haplotype carrier status to predict phenotype individually and combined (Full model). Models for only translocation carriers (T-carriers) were also fitted using the haplotypes excluding chromosome arms containing the translocation breakpoints (e.g. chr5q, chr1p, chr2p, chr3q, chr4q, chr16p). AOO-F – Age of onset Model F. b) using PRS to predict phenotype individually, fitted together (Full model) and in a multiple regression backwards selection model (Final model). c) using PRS or translocation status to predict phenotype individually after conditioning on a model containing all linkage regions (Minus All) or all PRS (Minus PRS) respectively; fitted together (Combined models) and in a multiple regression backwards selection model (Final model).

Supplementary Table 6a-e Two-point LOD scores for the haplotype regions LOD>=3.3: a) chr1q; b) chr11q1; c) chr11q2; and d) chr5q and e) regions LOD>2

See F23 WGS Supplementary_Tables_MolPsych.xls

Each SNP ID additionally annotated with: the allele on the disease associated haplotype (Haplo); the 1000 Genomes Phase 3 EUR alleles (A1 and A2) and corresponding allele frequencies (AF1, AF2); gene annotations if within the boundaries of a gene, the name of that gene (Gene name) and whether the SNP is exonic or intronic; the nearest genes 10kb upstream and downstream of the variants; whether the variant is located within a known gene promoter, VEP Consequence, IMPACT, aa change and SIFT and PolyPhen results.

Supplementary Table 7 Variant Effect Predictor annotation

Region	Variant ID	Position (hg19)	LOD	Haplo Allele	MAF*	VEP IMPACT	Transcript	GENE	Amino Acids	SIFT	PolyPhen
chr1q	rs5744174	223284528	1.95	A	0.41	MODERATE	ENST00000342210	*TLR5*	F/L	tolerated(1)	benign(0.001)
	rs2072493	223284599	1.02	T	0.17	MODERATE	ENST00000342210	*TLR5*	N/I	tolerated(0.21)	benign(0.003)
	rs5744168	223285200	1.55	G	0.06	HIGH	ENST00000342210	*TLR5*	**R/***	-	-
	rs45528236	223285833	1.55	G	0.06	MODERATE	ENST00000342210	*TLR5*	Q/K	tolerated(0.62)	benign(0)
	rs764535	223286129	0.63	G	0.01	MODERATE	ENST00000342210	*TLR5*	T/I	tolerated(0.08)	benign(0.029)
	rs10907376	223567803	**4.75**	A	0.27	MODERATE	ENST00000366875	*CCDC185*	G/D	tolerated(0.63)	**possibly_damaging(0.663)**
	rs4072247	223716508	0.08	C	0.10	MODERATE	ENST00000366872	*CAPN8*	E/K	-	benign(0.003)
	rs61823553	223718651	2.13	G	0.55	MODERATE	ENST00000366872	*CAPN8*	T/M	-	benign(0.445)
	rs35539373	223813586	2.04	T	0.52	MODERATE	ENST00000366872	*CAPN8*	S/Y	-	**possibly_damaging(0.834)**
	rs71644745	223816383	0.43	G	0.05	MODERATE	ENST00000366872	*CAPN8*	A/V	-	**possibly_damaging(0.579)**
	rs17599	223954080	1.21	A	0.25	MODERATE	ENST00000295006	*CAPN2*	K/Q	**deleterious(0.03)**	**possibly_damaging(0.46)**
	rs61824007	223985963	0.15	C	0.01	MODERATE	ENST00000343537	*TP53BP2*	Q/H	tolerated(0.56)	**probably_damaging(0.997)**
	rs61749337	223991959	0.15	G	0.01	MODERATE	ENST00000343537	*TP53BP2*	A/V	tolerated(1)	**probably_damaging(0.997)**
	rs34631151	224482084	1.26	C	0.04	MODERATE	ENST00000281701	*NVL*	V/I	**deleterious(0.04)**	**possibly_damaging(0.802)**
chr11q1	rs302646	87908448	0.78	G	0.13	MODERATE	ENST00000526372	*RAB38*	F/L	tolerated(1)	benign(0)
	rs3812730	87908563	2.63	G	0.27	MODERATE	ENST00000531138	*RAB38*	P/L	tolerated(0.11)	benign(0.006)
	rs3888798	88027209	1.12	C	0.052	MODERATE	ENST00000227266	*CTSC*	I/V	**deleterious(0.04)**	benign(0.244)
	rs217086	88045583	3.32	A	0.15	MODERATE	ENST00000227266	*CTSC*	I/T	tolerated(0.68)	benign(0)
	rs11600158	88070914	2.02	A	0.08	MODERATE	ENST00000527018	*CTSC*	V/A	-	unknown(0)
chr11q2	rs61749255	99715891	**4.48**	G	0.00	MODERATE	ENST00000279463	*CNTN5*	I/M	**deleterious(0.03)**	benign(0.138)
chr5q	rs150642131	64273045	2.55	T	0.00	MODERATE	ENST00000381070	*CWC27*	E/D	tolerated(0.54)	benign(0.004)

*MAF – 1000 Genomes Phase 3

Supplementary Table 8 Functional investigation of top LOD variants within the phenotype-associated haplotypes: a) Missense b) chr1q; c) chr11q1; d) chr11q2; and e) chr5q and f) Regions LOD>2

See F23 WGS Supplementary_Tables_MolPsych.xls

These data combine: prediction of regulatory function from Regulome DB; annotation of whether the SNP is a brain specific eQTL (as annotated by BRAINEAC), including the tissue that shows most significant association; LOD scores (and associated model), haplotype allele, major and minor allele frequencies.

Supplementary Table 9 Association summary: a) Affective disorder GS:SFHS & UKB (p<0.001) b) Cognitive and mental health-related traits

See F23 WGS Supplementary_Tables_MolPsych.xls

Supplementary Table 10 Association of haplotypes with global function and cognitive variables in the t(1;11) family

**p-value***	**t(1;11)**	**chr1q**	**chr11q1**	**chr11q2**	**chr5q**	**chr1p**	**chr2p**	**chr3q**	**chr4q**	**chr16p**
GAF	0.053	**0.010**	**5.6×10^{-4}**	0.062	**1.5×10^{-3}**	0.350	0.233	0.546	0.565	0.543
Current_IQ	0.812	0.697	0.700	0.422	0.585	0.283	0.531	0.960	0.596	0.967
Attn_ProcessingSpeed	0.791	0.791	0.969	0.265	0.141	0.250	0.542	0.795	0.535	0.440
Carriers GAF (N)	14	16	17	14	16	10	2	11	9	18
Non-carriers GAF (N)	24	22	21	21	22	28	36	27	29	20
Carriers Current_IQ (N)	15	14	15	13	14	8	2	6	8	16
Non-carriers Current_IQ (N)	13	14	13	13	14	20	26	22	20	12
Carriers Attn_Processing (N)	13	13	14	13	13	6	2	5	7	15
Non-carriers Attn_Processing (N)	13	13	12	12	13	20	24	21	19	11

*adjusted for age and sex

GAF – Global Assessment of Functioning; Current IQ and Attn/ProcessingSpeed - attention/processing speed; bold p<0.05.

Supplementary Figures

Supplementary Figure 1 Individual sample coverage and minor allele frequencies. a. Individual sample coverage: mean read depth, percentage bases at 10x and 20x. b. Minor allele frequencies (MAF) of SNV identified in t(1;11) family in 1000 Genomes phase 3 European sample (EUR) and GnomAD non-Finnish European (NFE). 1c. PCA plots before and after filtering variants by Mendelian segregation.

1a. Individual sample coverage.

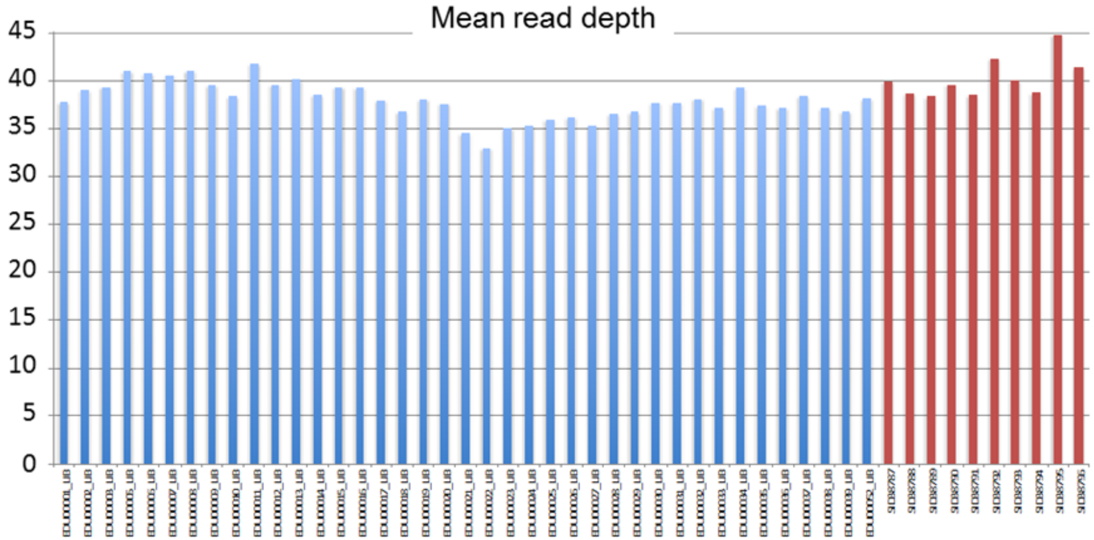

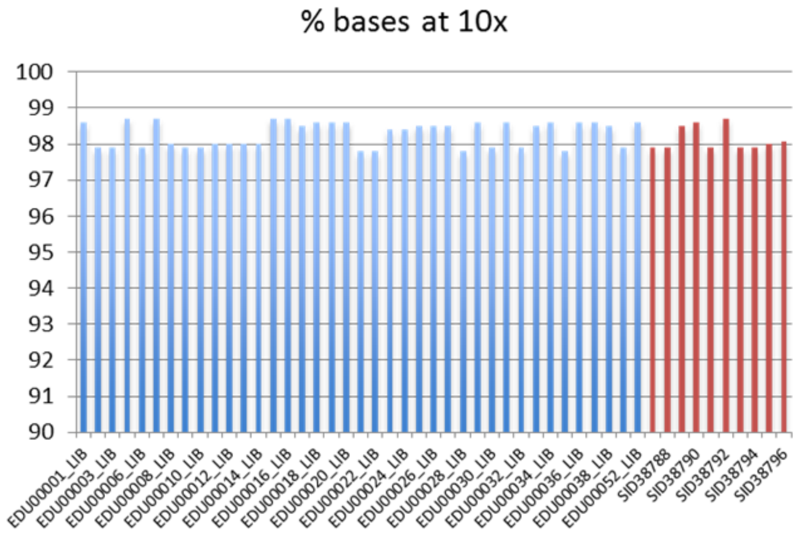

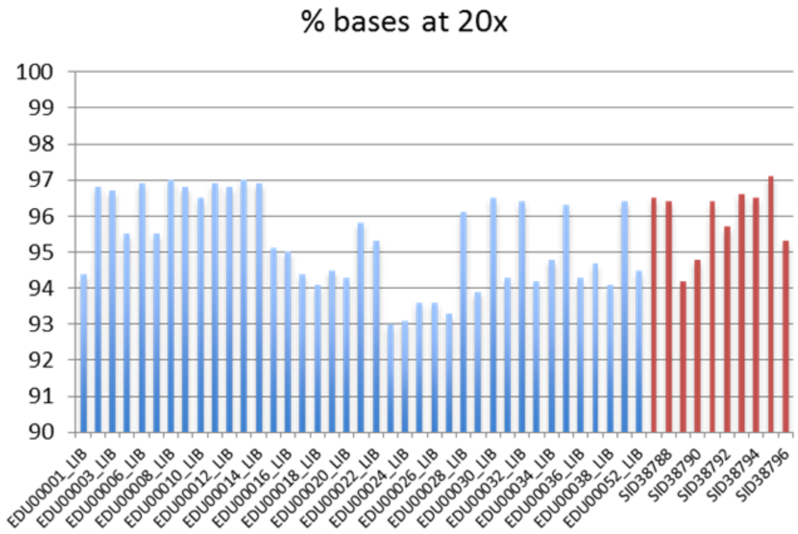

1b. Minor allele frequencies (MAF) of SNVs in 1000 Genomes phase 3 European sample (EUR) and GnomAD non-Finnish European (NFE).

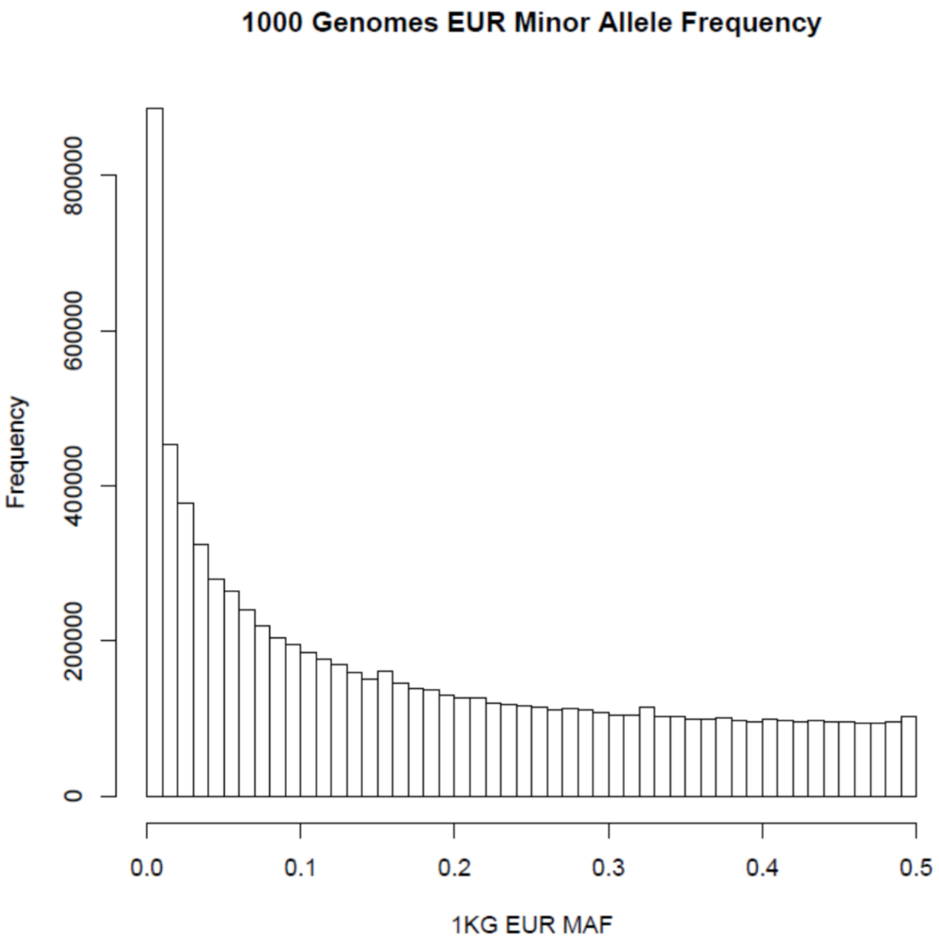

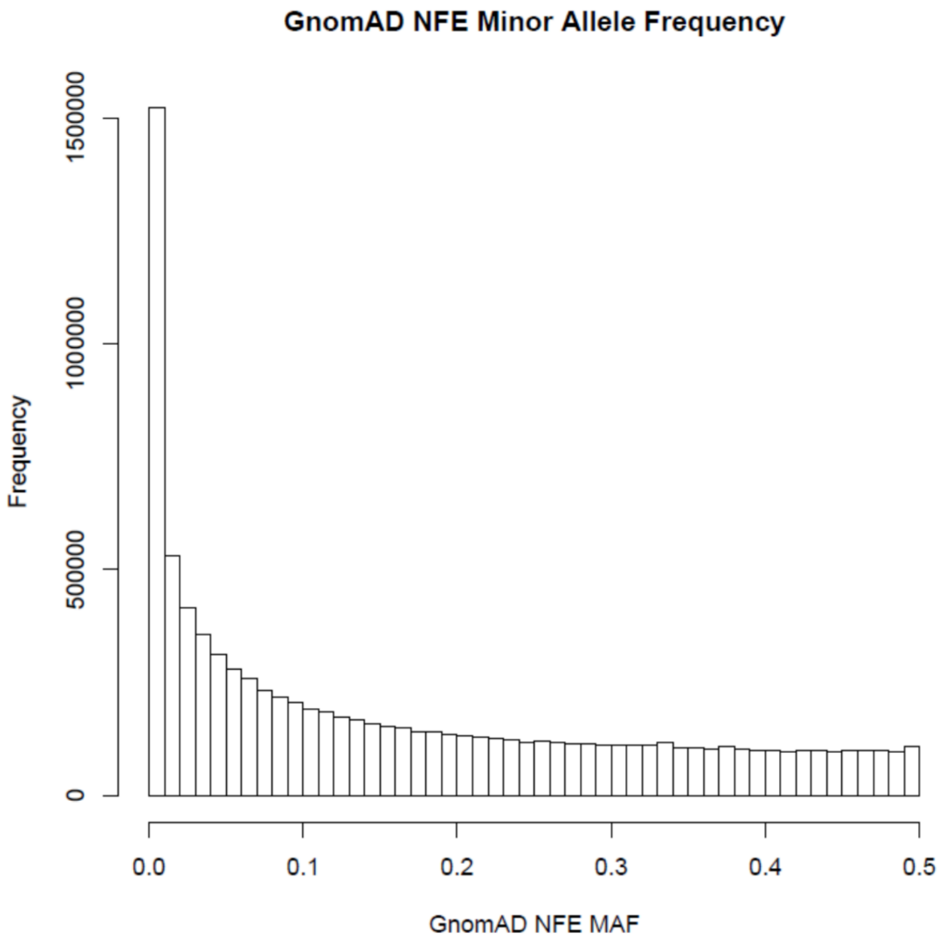

1c. PCA plots before and after filtering variants by Mendelian segregation.

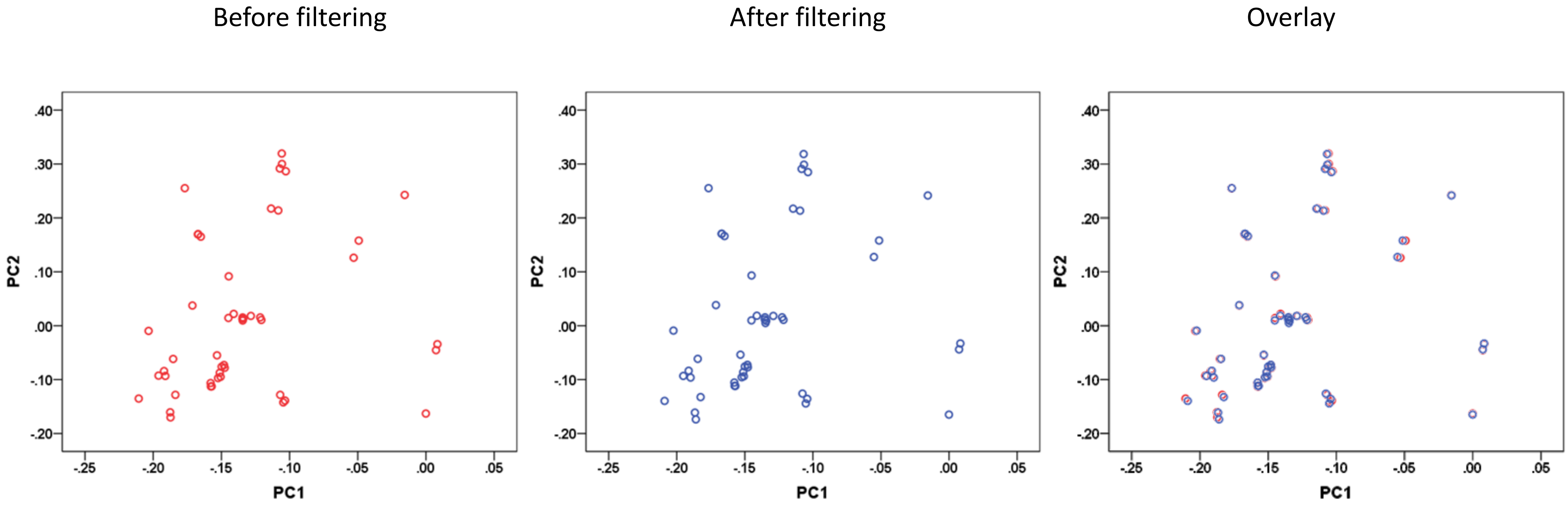

Supplementary Figure 2 Multipoint linkage analysis of the t(1;11) family. Plots of all chromosomes. The plots represent multipoint LOD scores vs. chromosome position in Mb. Multipoint LODs = 1, 2 and 3 annotated with grey, blue and red horizontal dotted lines respectively.

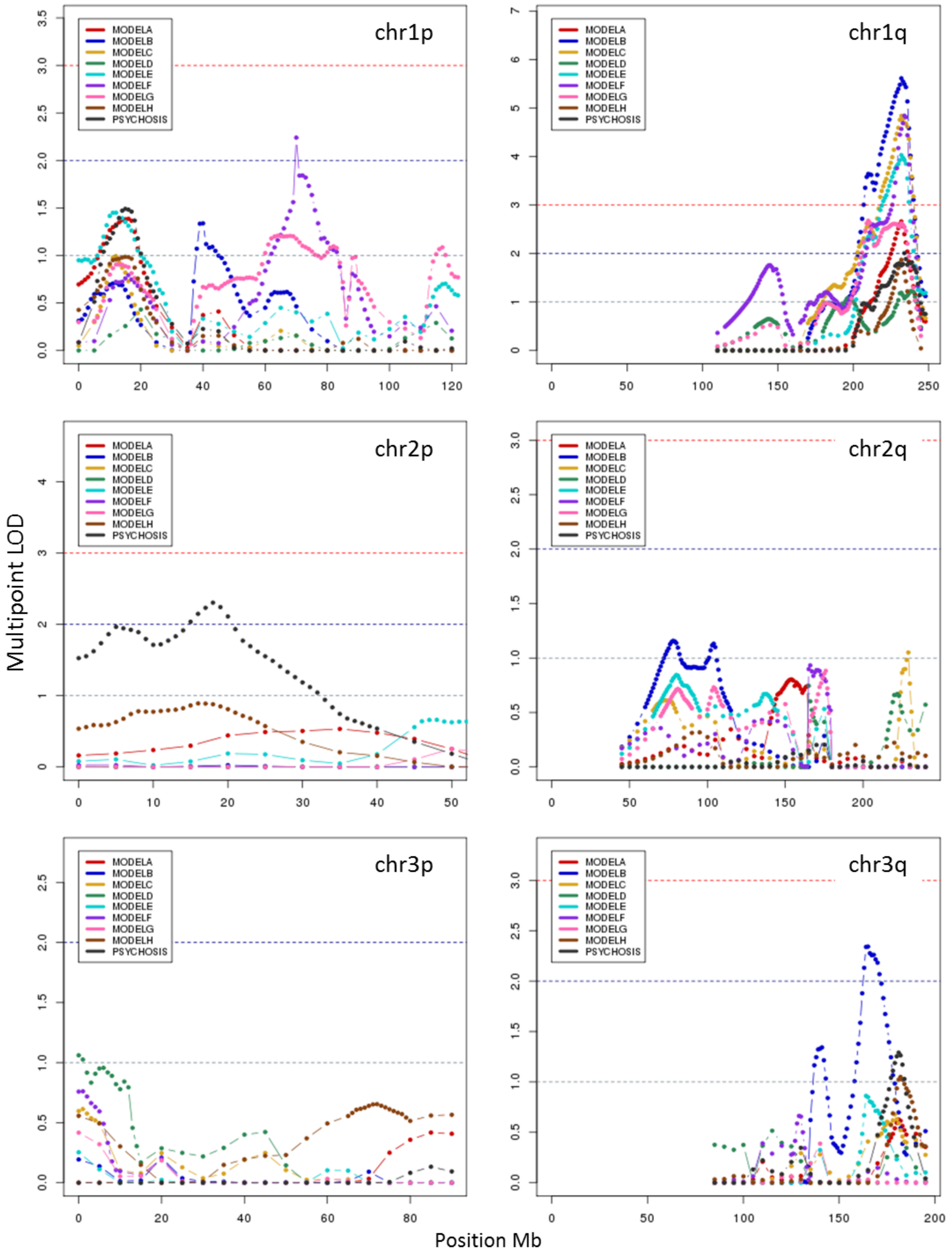

Supplementary Figure 2 Multipoint linkage analysis of the t(1;11) family. Cont…

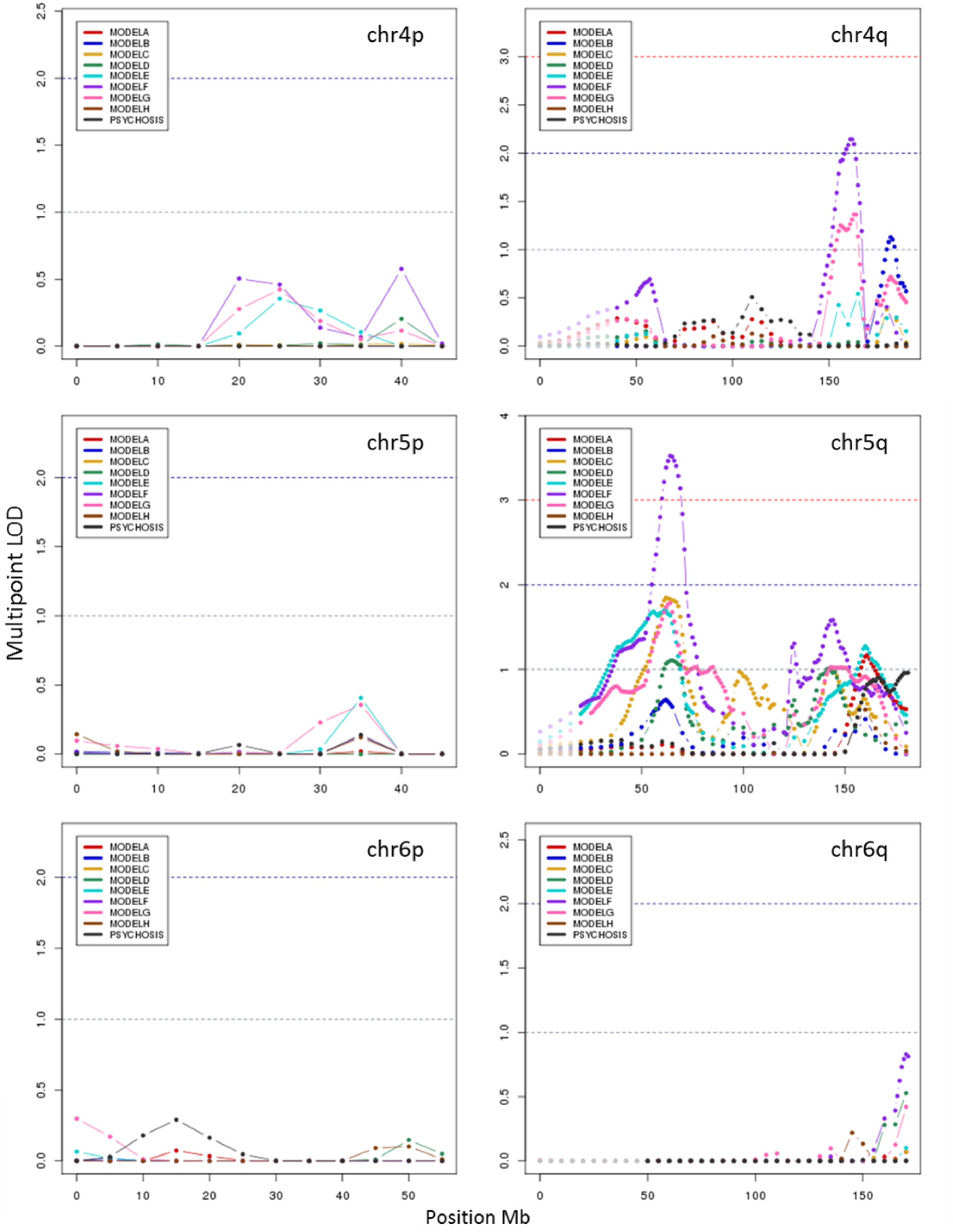

Supplementary Figure 2 Multipoint linkage analysis of the t(1;11) family. Cont…

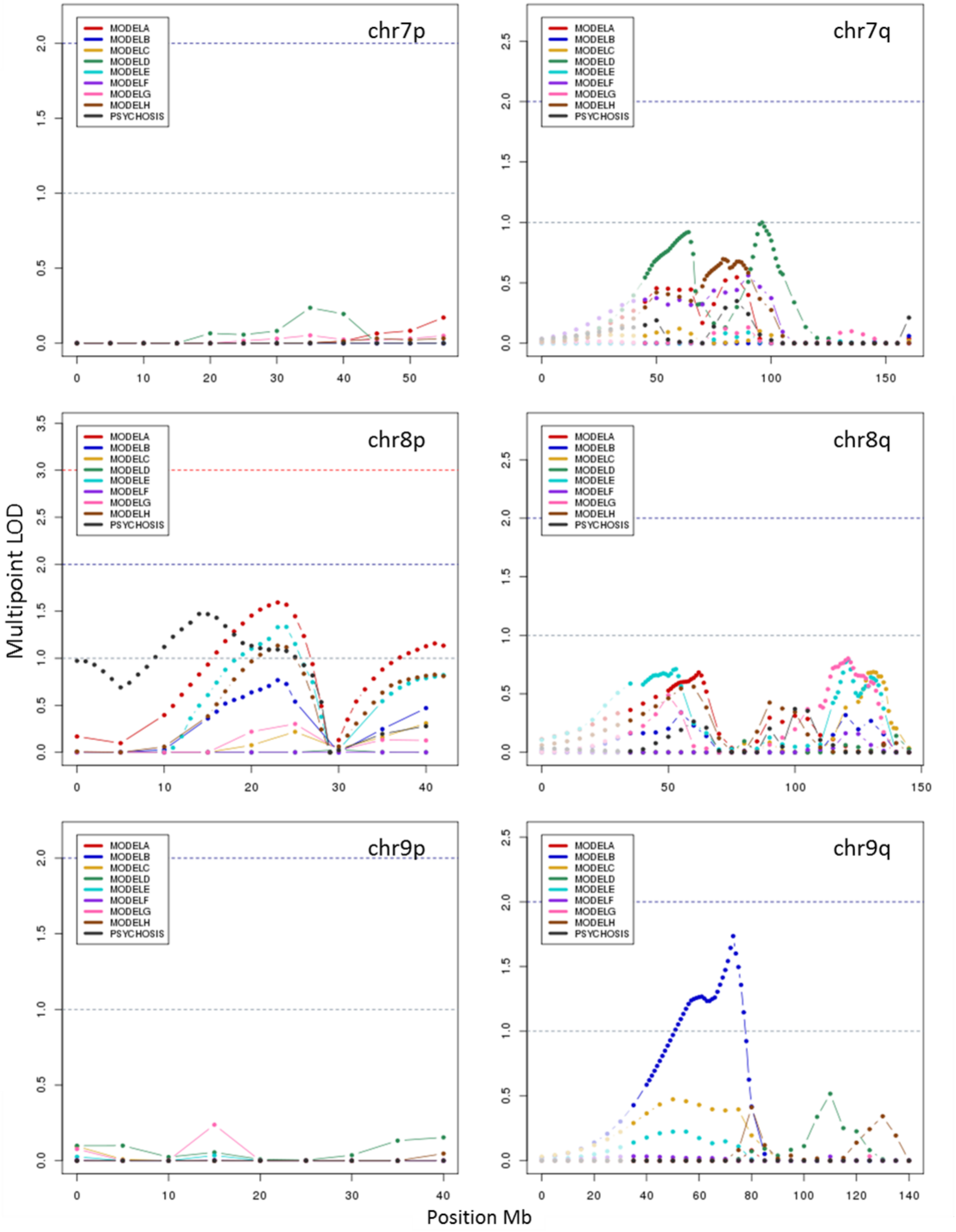

Supplementary Figure 2 Multipoint linkage analysis of the t(1;11) family. Cont…

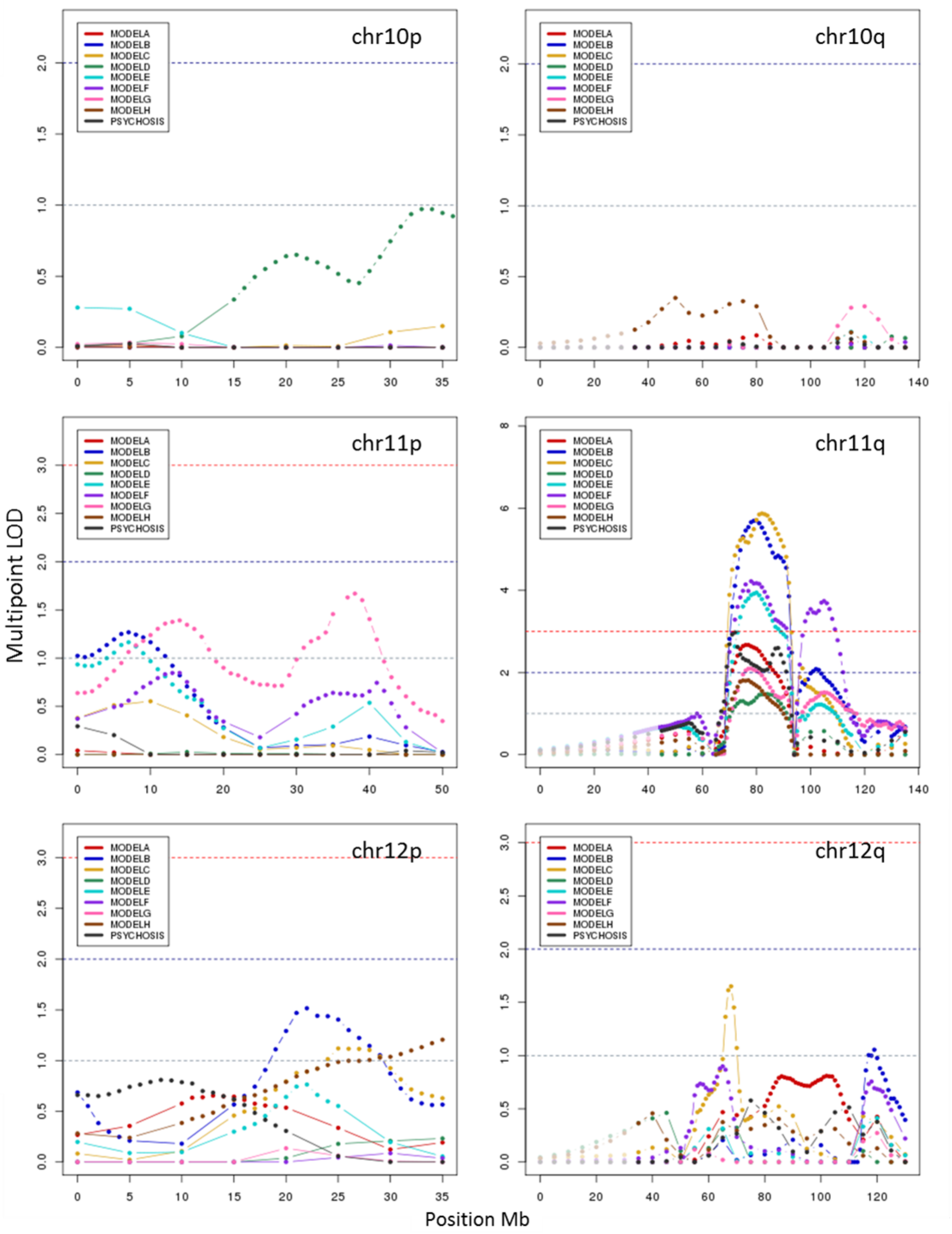

Supplementary Figure 2 Multipoint linkage analysis of the t(1;11) family. Cont…

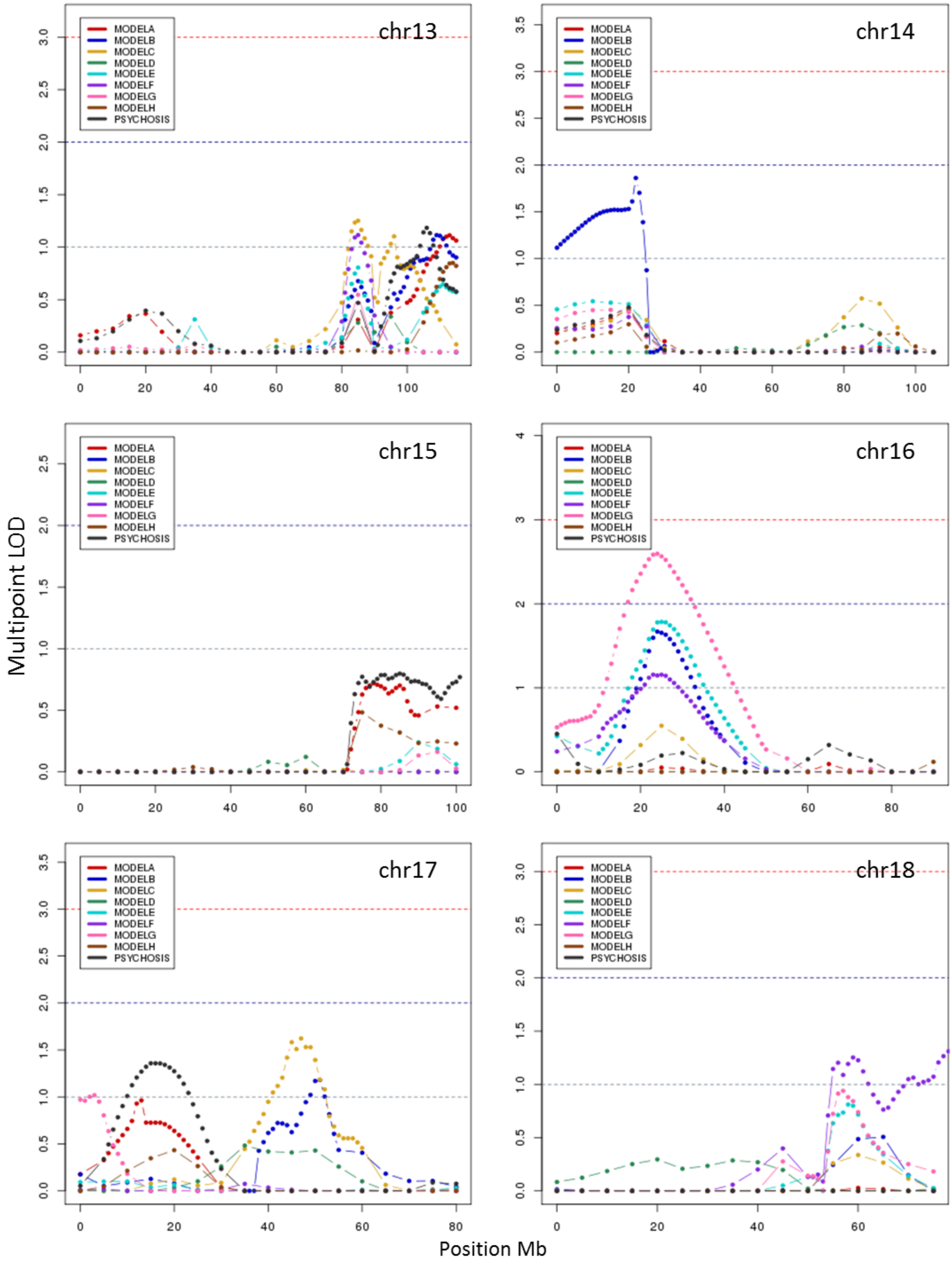

Supplementary Figure 2 Multipoint linkage analysis of the t(1;11) family. Cont…

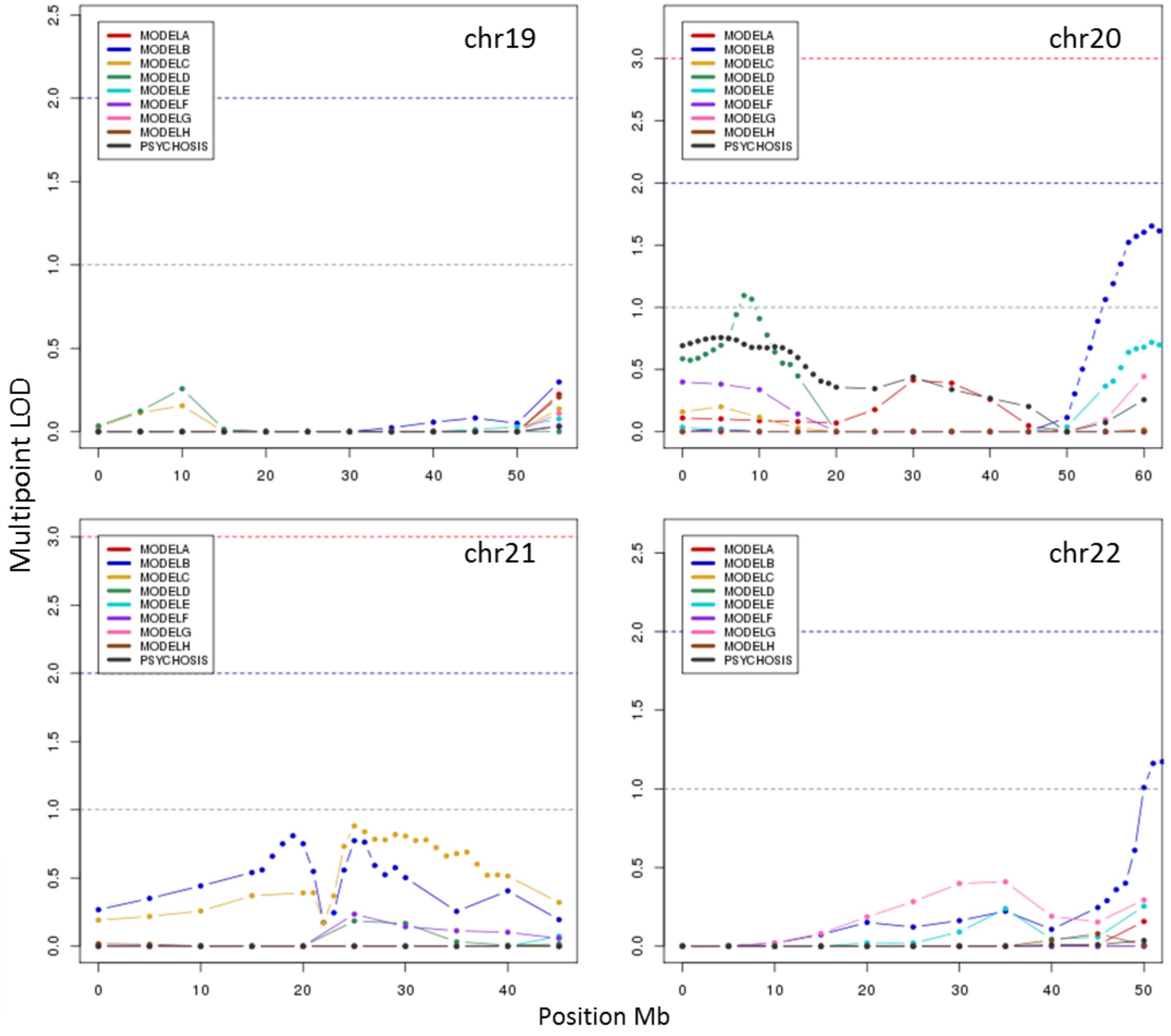

Supplementary Figure 3. Disease-associated haplotypes. a. chr1q; b. chr11q1 & chr11q2; and c. chr5q, d. derived chr1 and e. derived chr11, showing extent of haplotype sharing. The black blocks mark the boundaries of the minimum haplotypes; translocation breakpoint ("T") is marked as a grey vertical line; red haplotypes are affected individuals; blue haplotypes unaffected individuals. The individual IDs and translocation status (T: translocation carrier) are shown on the left, phenotypes are shown above each haplotype. The centromeric boundaries of all 3 haplotype regions are defined by a single recombination breakpoint where multiple haplotype carriers diverge. This is also the case for the telomeric boundary of the chr1q region. These are marked with black lines.

3a. Chr1q

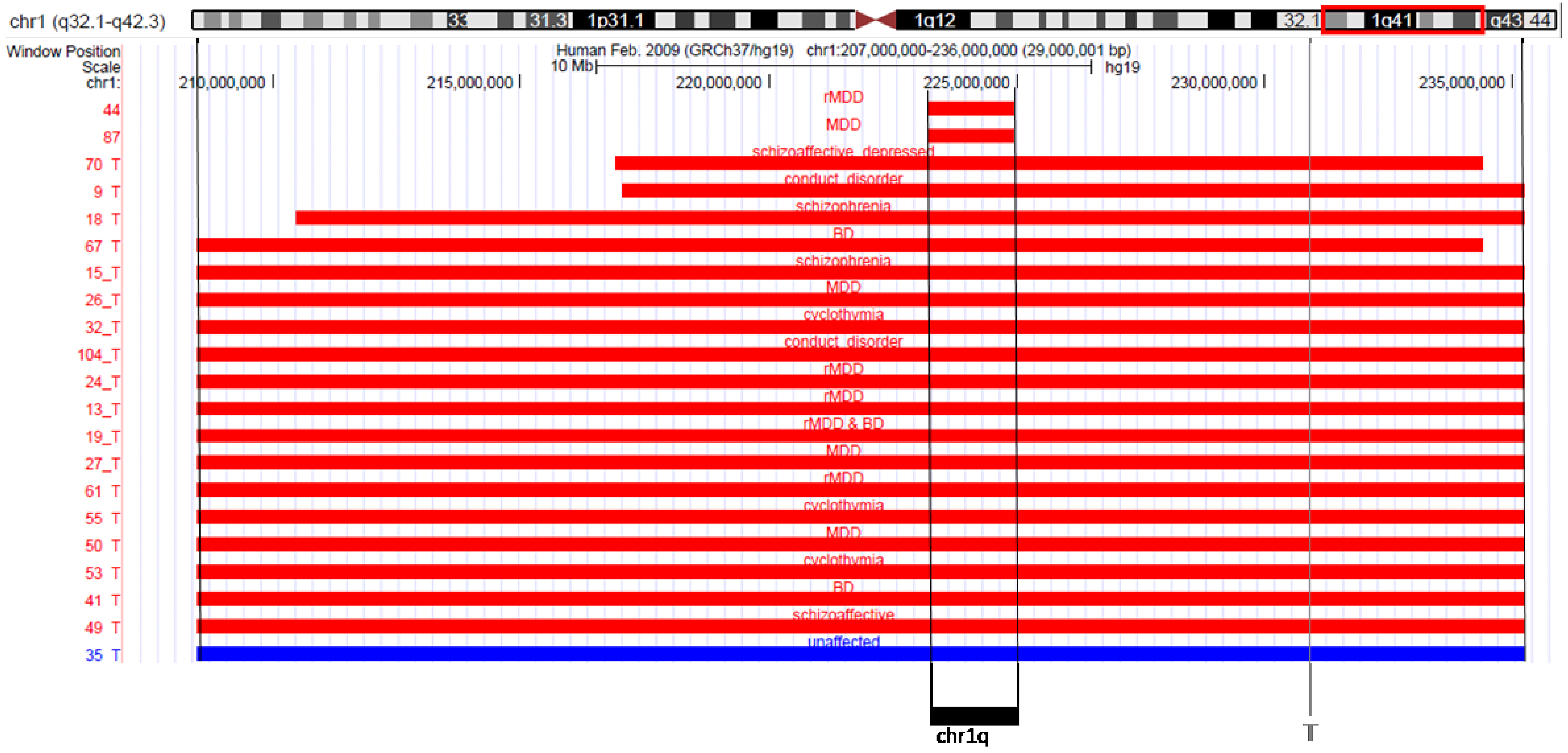

3b. Chr11q1 and chr11q2

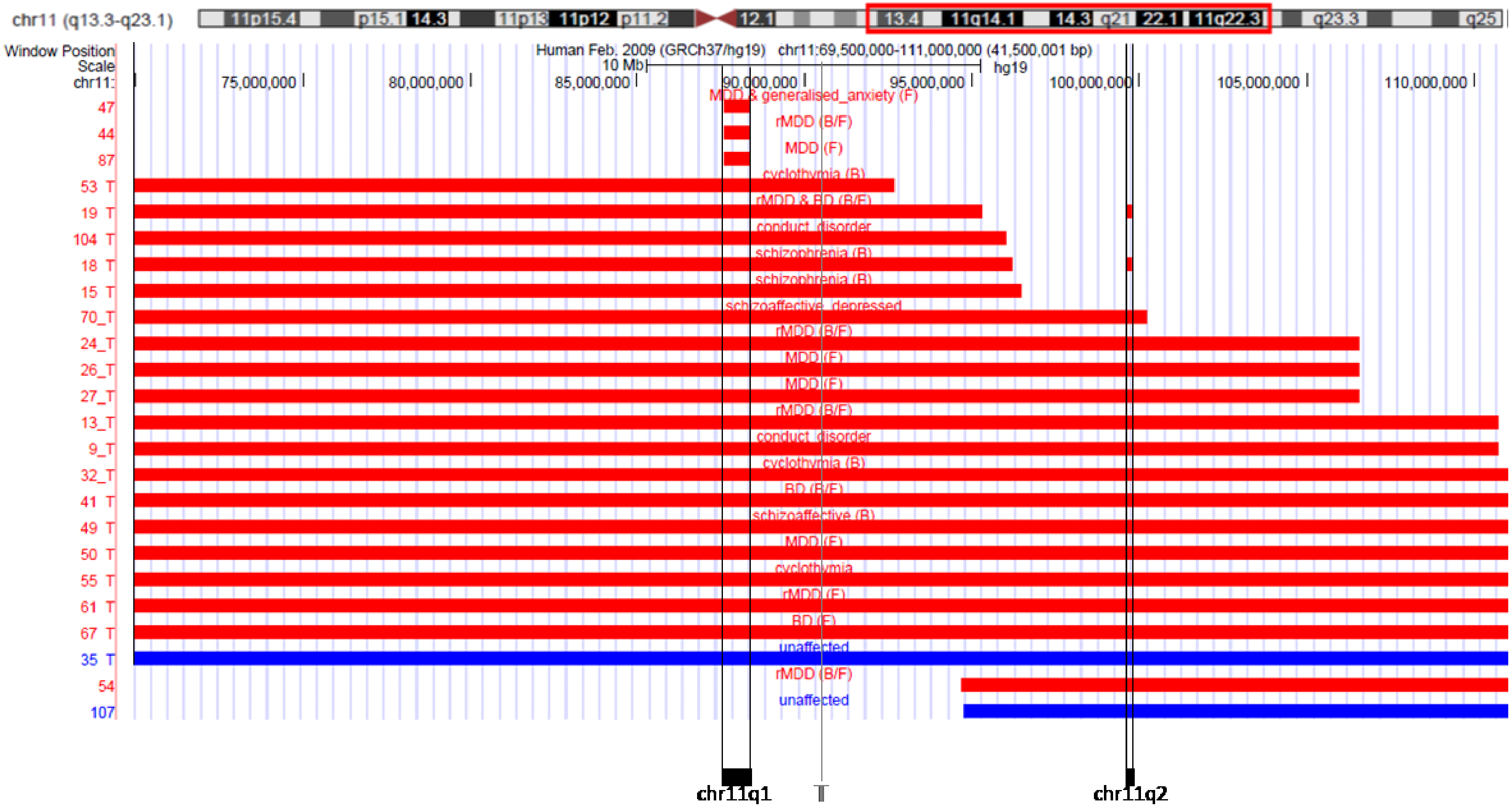

3c. Chr5q

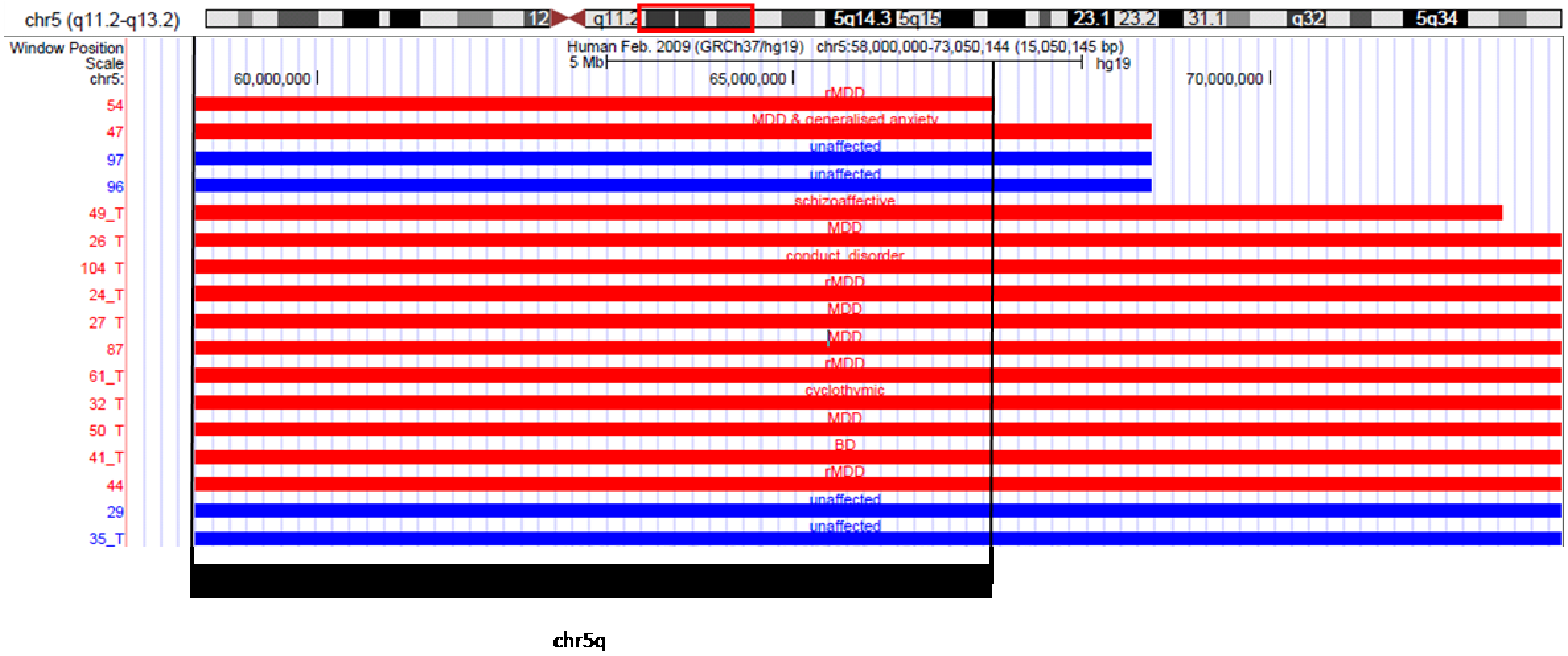
chr5 (q11.2-q13.2)
Window Position
Scale
chr5:
Human Feb. 2009 (GRCh37/hg19) chr5:58,000,000-73,050,144 (15,050,145 bp)
5 Mb
hg19
60,000,000
65,000,000
70,000,000
54 rMDD
47 MDD & generalised anxiety
97 unaffected
96 unaffected
49_T schizoaffective
26_T MDD
104_T conduct_disorder
24_T rMDD
27_T MDD
87 MDD
61_T rMDD
32_T cyclothymic
50_T MDD
41_T BD
44 rMDD
29 unaffected
35_T unaffected
chr5q

3d. t(1;11) derived chromosome 1

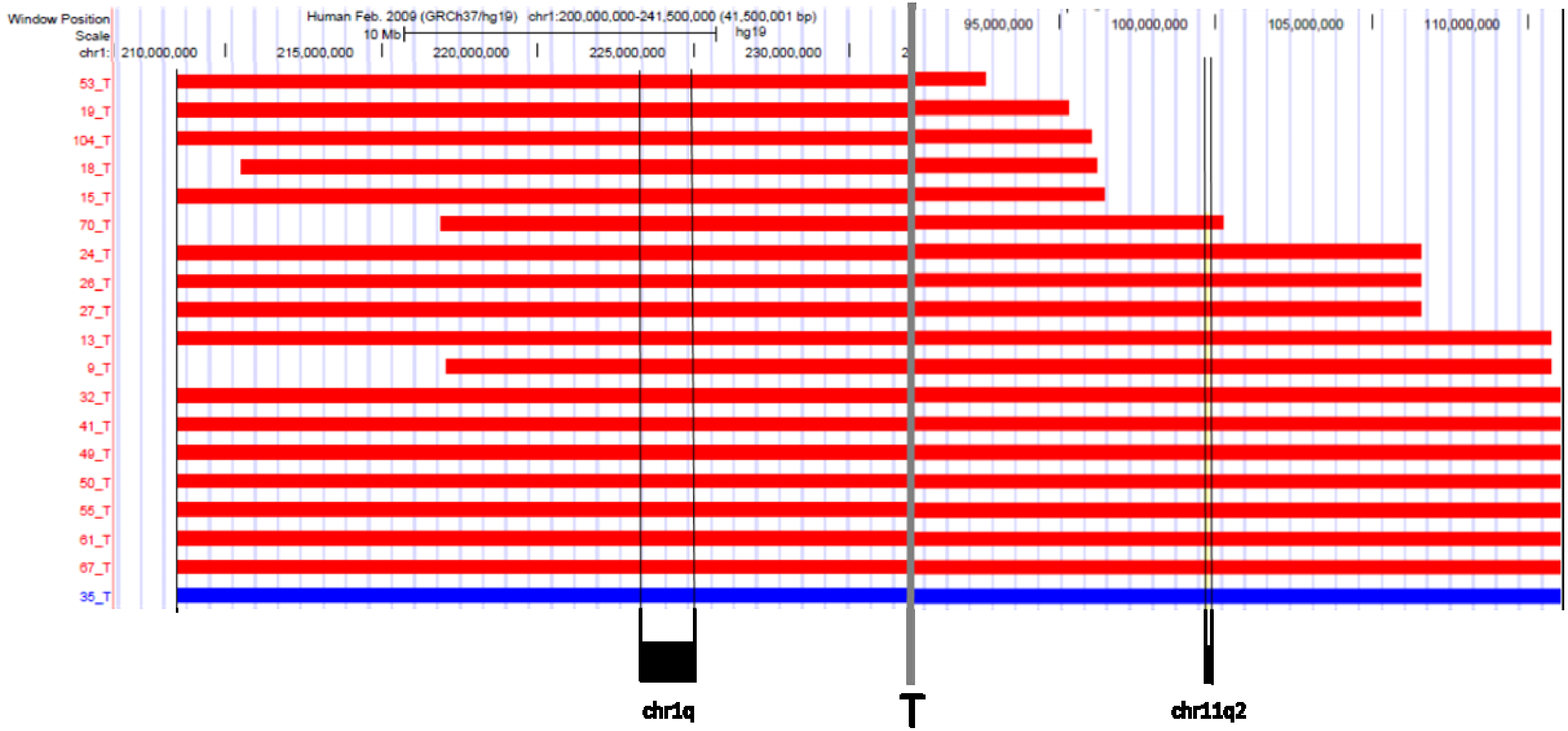

3e. t(1;11) derived chromosome 11

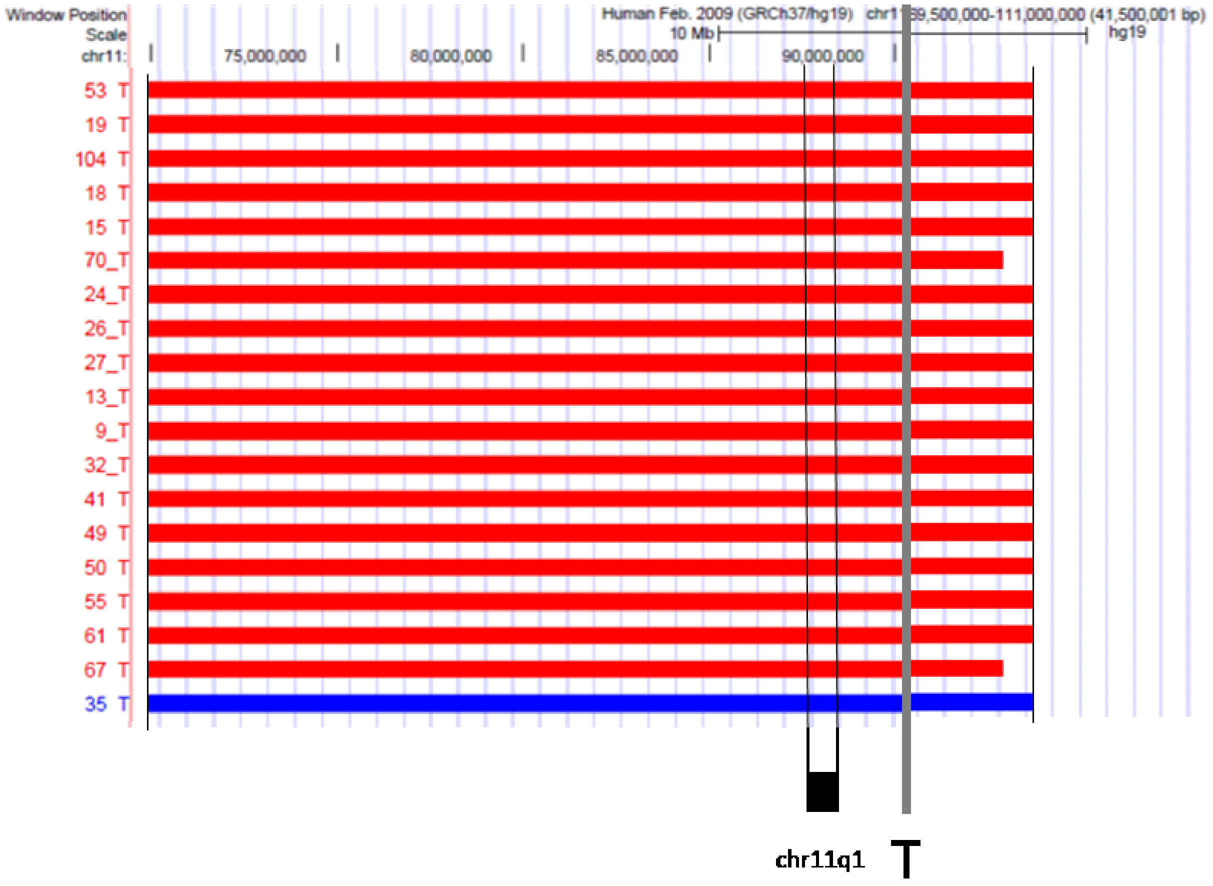

Supplementary Figure 4: Intra-family regional correlations

**Spearman's rho**	Correlations									
	t(1;11)	chr1q	chr11q1	chr11q2	chr5q	chr1p	chr2p	chr3q	chr4q	chr16p
t(1;11)	1	.918**	.880**	.781**	.291*	-0.101	.466**	0.065	0.109	.367*
chr1	.918**	1	.959**	.705**	.401**	0.065	.428**	0.219	0.168	.453**
chr11q1	.880**	.959**	1	.669**	.455**	0.146	.410**	0.195	0.249	.413**
chr11q2	.781**	.705**	.669**	1	.326*	-0.079	.360*	-0.013	0.238	.410**
chr5	.291*	.401**	.455**	.326*	1	0.264	-0.28	0.114	.478**	0.28
chr1p	-0.101	0.065	0.146	-0.079	0.264	1	-0.194	.331*	.495**	0.043
chr2p	.466**	.428**	.410**	.360*	-0.28	-0.194	1	-0.206	-0.194	0.147
chr3q	0.065	0.219	0.195	-0.013	0.114	.331*	-0.206	1	0.209	.294*
chr4q	0.109	0.168	0.249	0.238	.478**	.495**	-0.194	0.209	1	0.249
chr16p	.367*	.453**	.413**	.410**	0.28	0.043	0.147	.294*	0.249	1

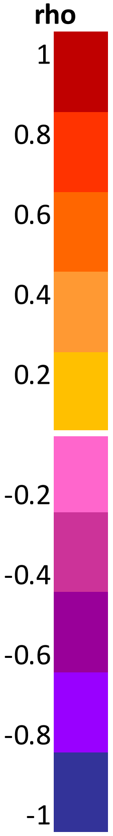

**. Correlation is significant at the 0.01 level (2-tailed).
*. Correlation is significant at the 0.05 level (2-tailed).

Supplementary Figure 5a-e. Regional two-point summaries - variants across the LOD ≥ 3.3 linkage regions. a) chr1q; b) chr11q1; c) chr11q2; and d) chr5q. Top: the two-point LOD scores across all nine models are plotted against Mb position. Middle: UCSC genes. Bottom: GS:SFHS and UKB association results for affective disorder left axis: --log(p-values), right axis: recombination rate cM/Mb GBR 1000 Genomes (grey line).

5a. Chr1q – chr1: 223246661-224932082

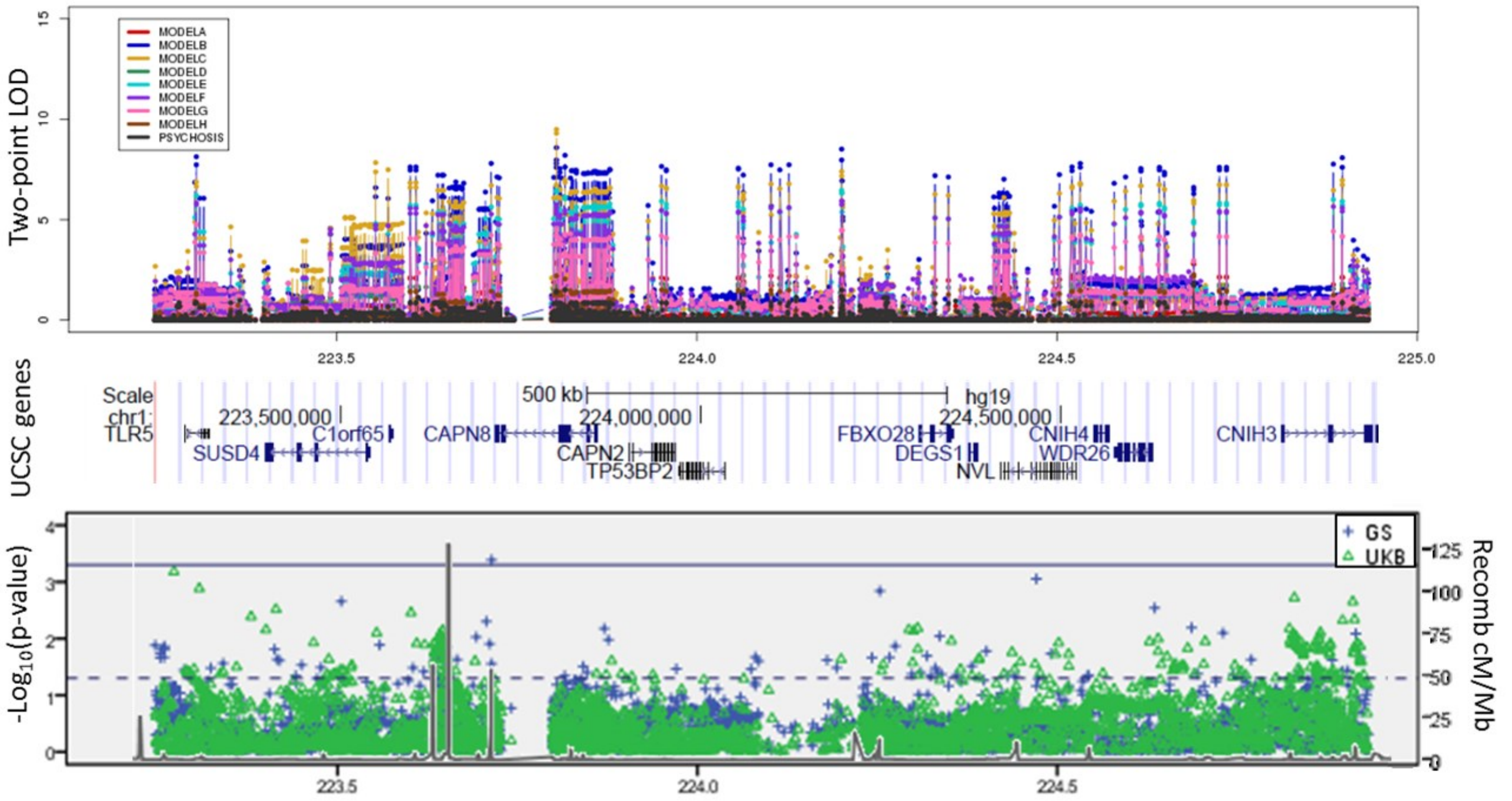

5b. Chr11q1 - chr11: 87597790-88350687

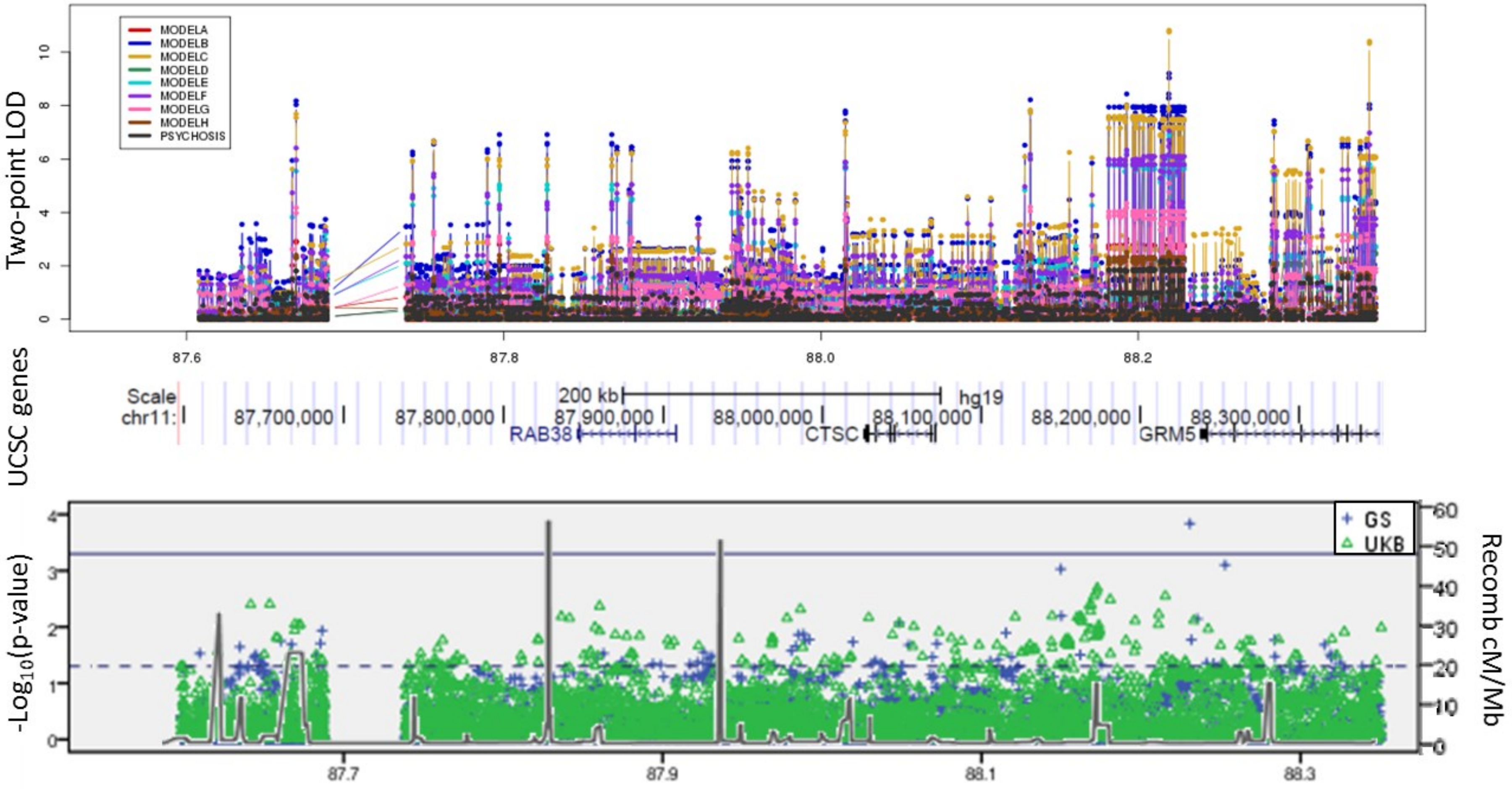

5c. Chr11q2 – chr11: 99616360-99753867

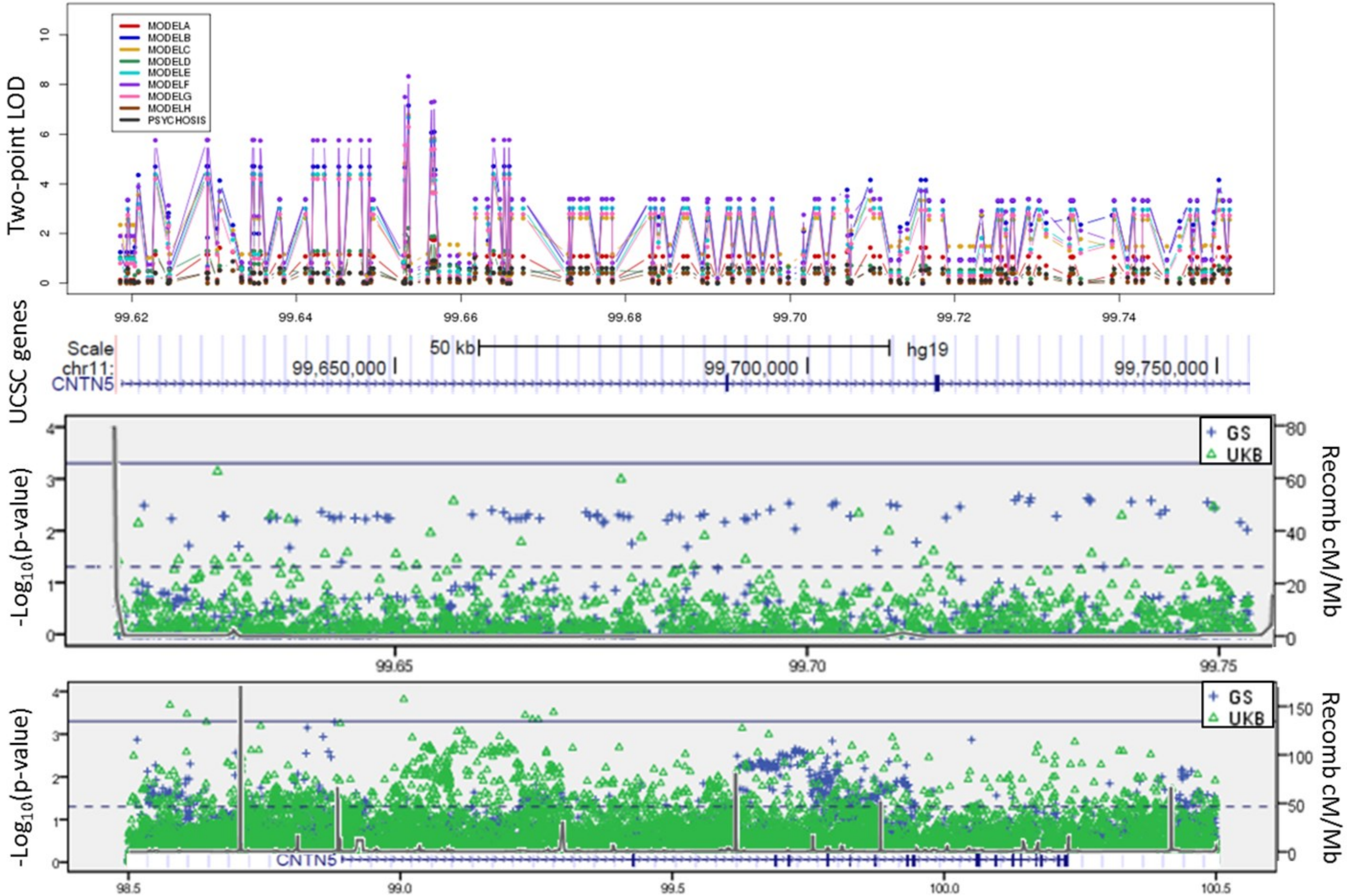

5d. Chr5q – chr5: 58721609-67098280

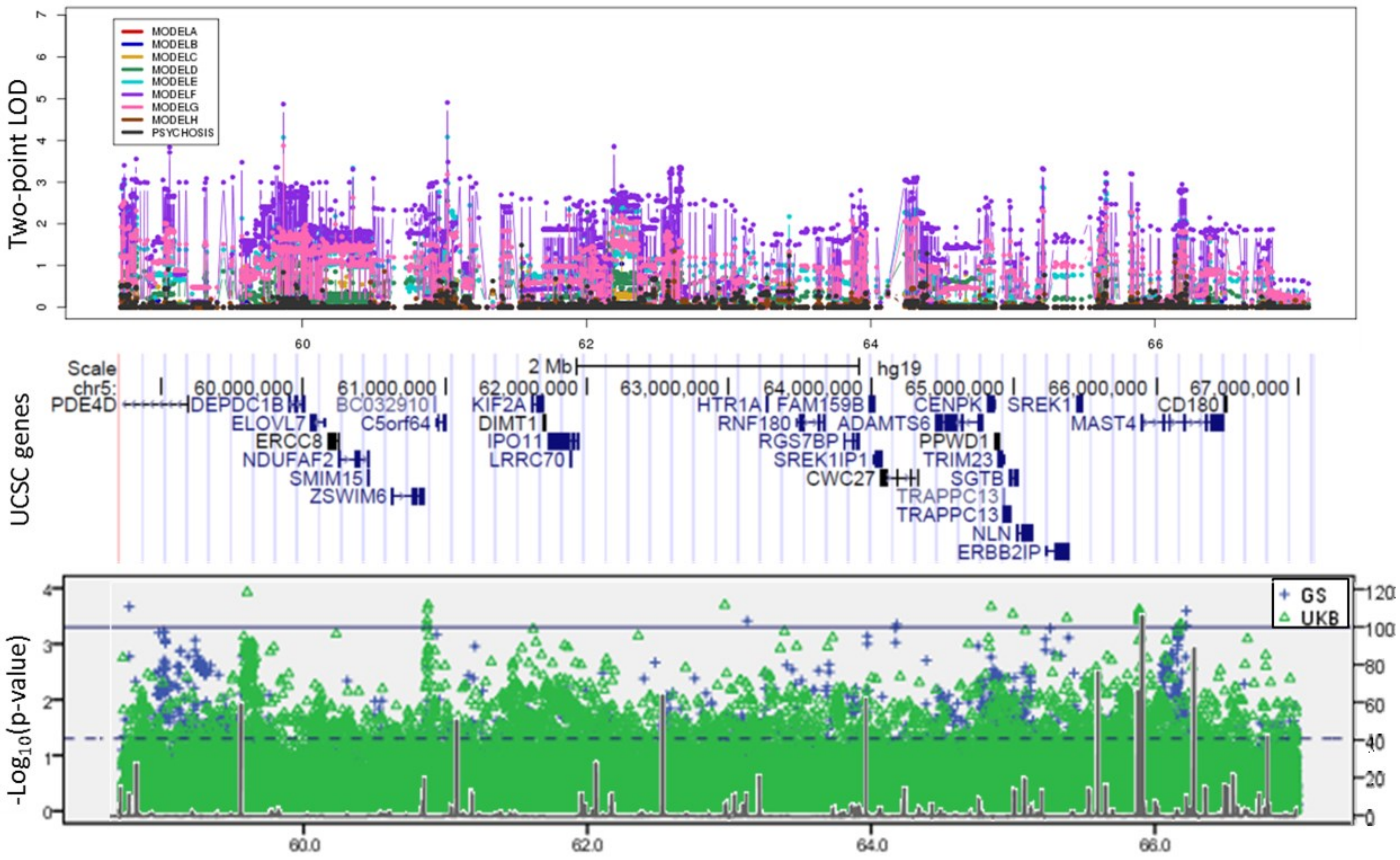

Supplementary Figure 6a-e. Regional two-point summaries - variants across the LOD ≥ 2 linkage regions. a) chr1p; b) chr2p; c) chr3q; d) chr4q; and e) chr16q. Top: the two-point LOD scores across all nine models are plotted against Mb position. Middle: UCSC genes. Bottom: GS:SFHS and UKB association results for affective disorder left axis: --log(p-values), right axis: recombination rate cM/Mb GBR 1000 Genomes (grey line).

6a**.** chr1p – chr1: 69000000-71000000

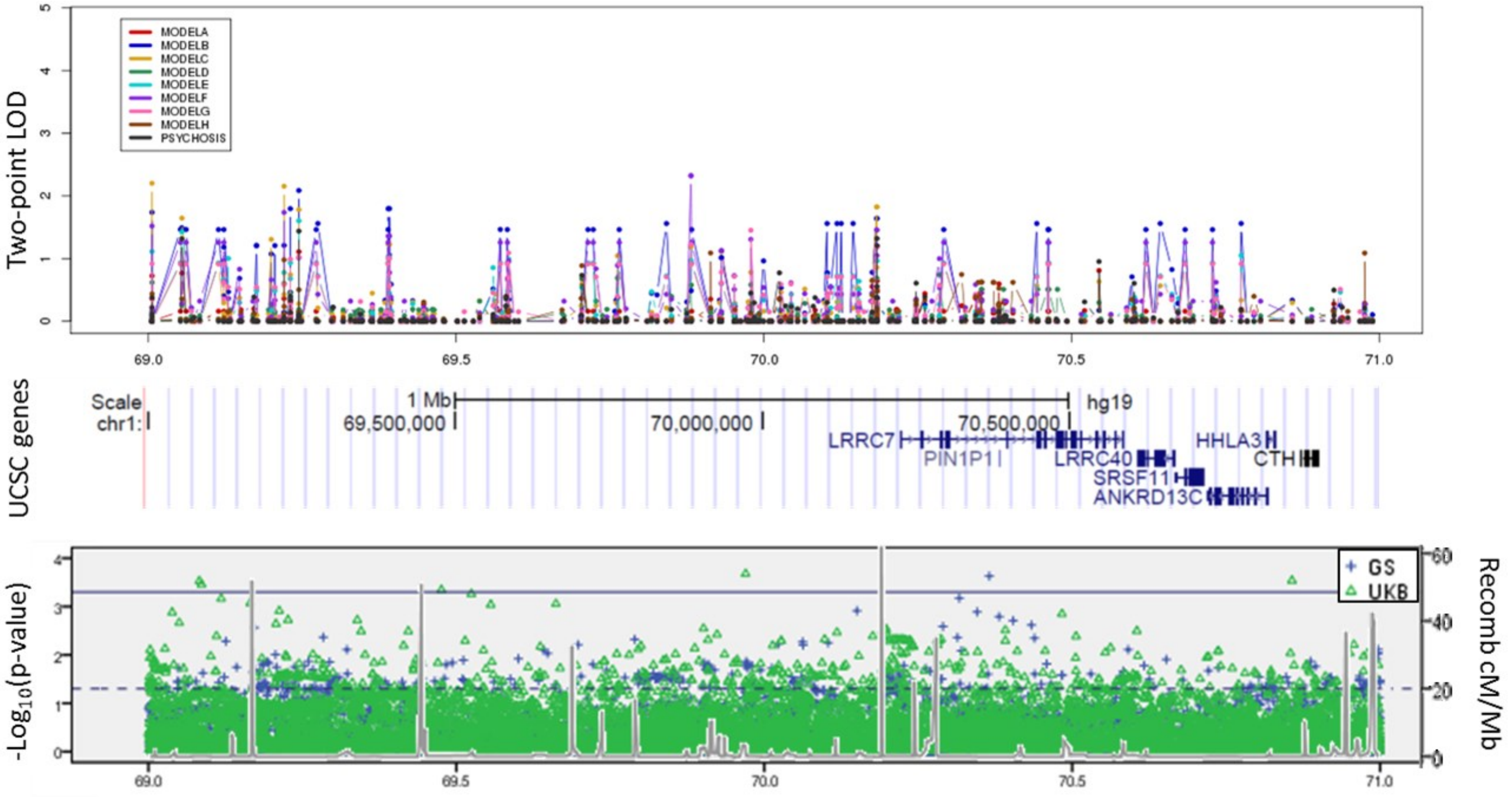

6b. chr2p – chr2: 17768000-18351000

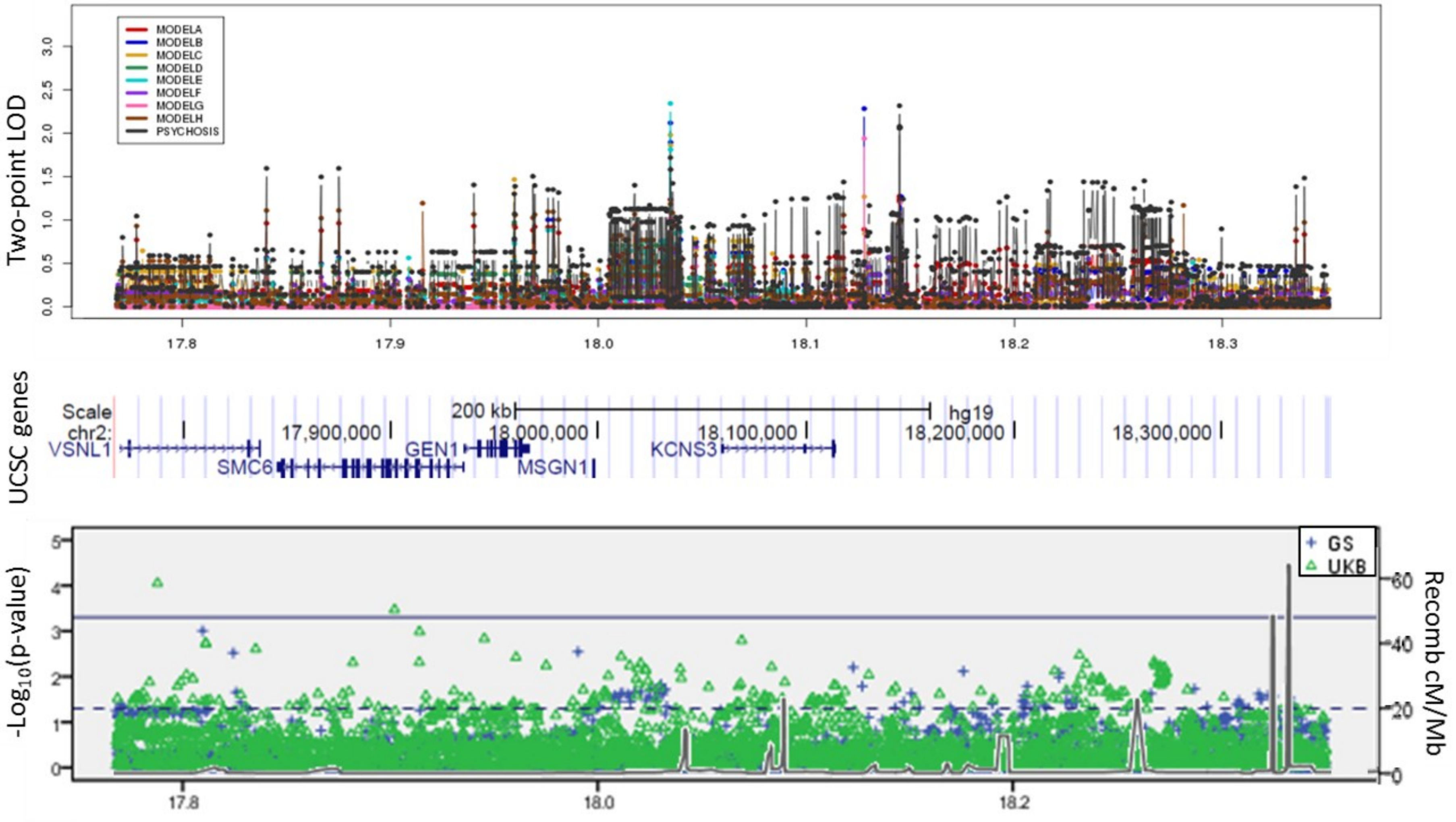

6c. chr3q – chr3: 162000000-172000000

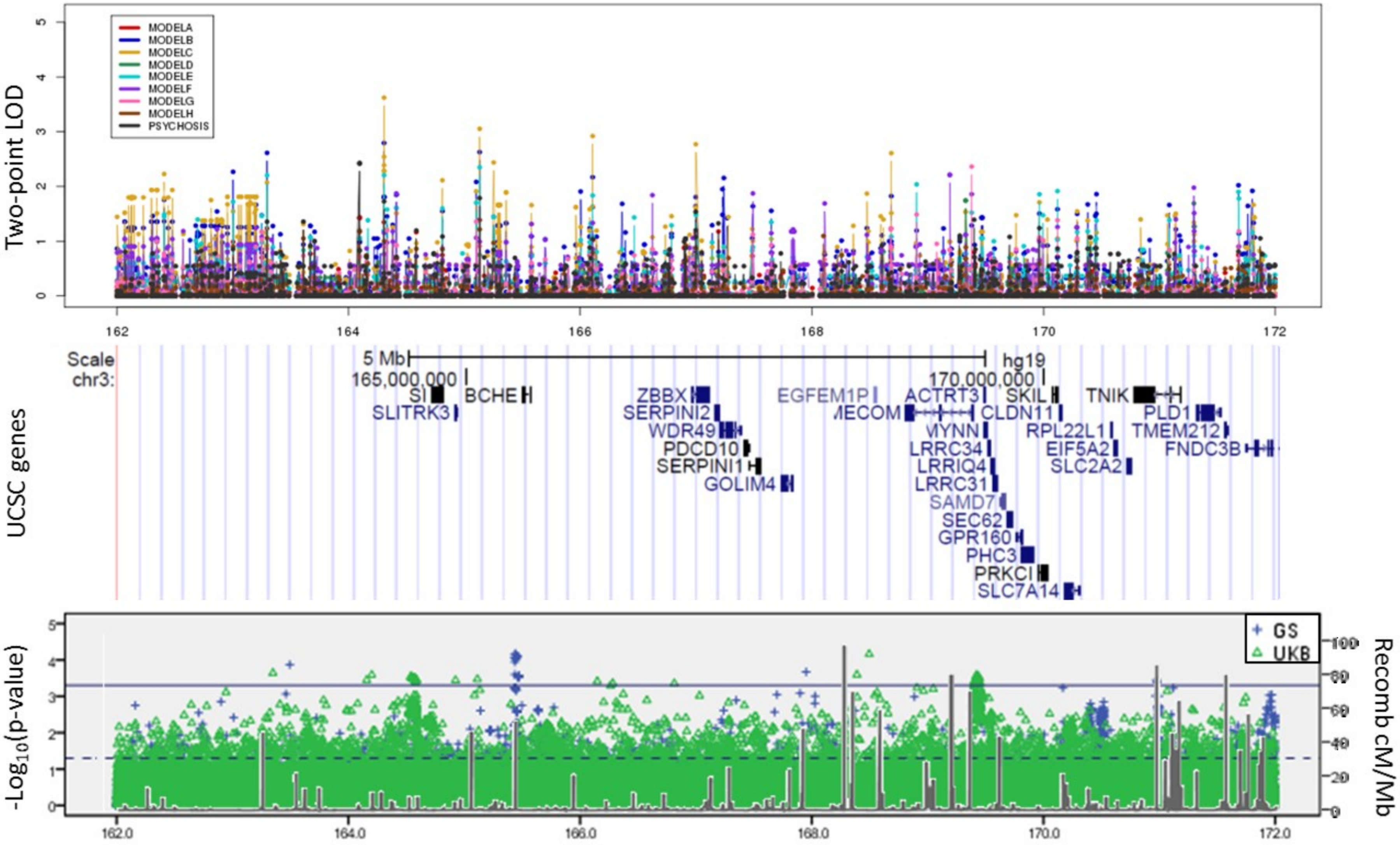

6d. chr4q – chr4: 158000000-164000000

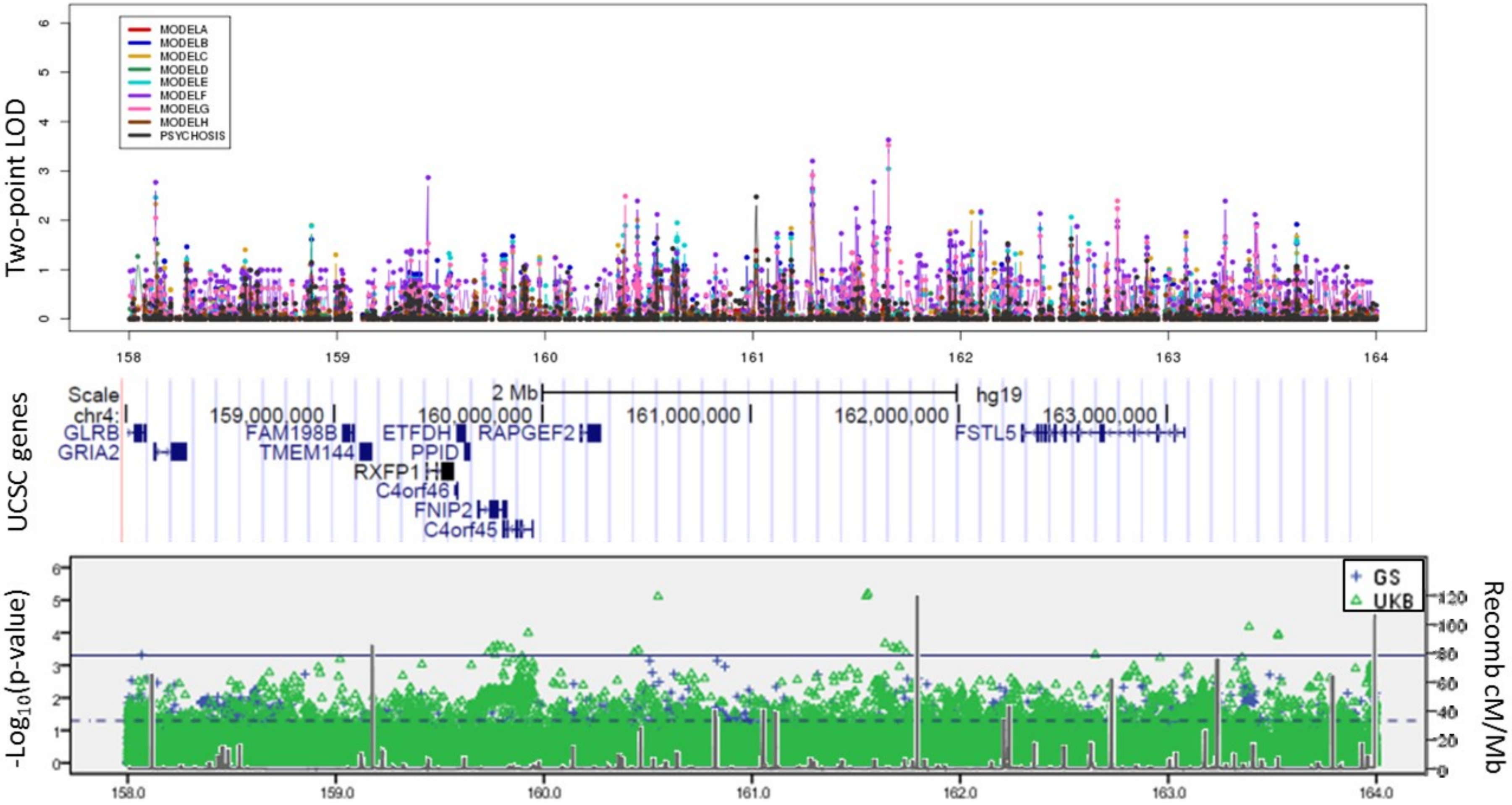

6e. chr16p – chr16: 16000000-33000000

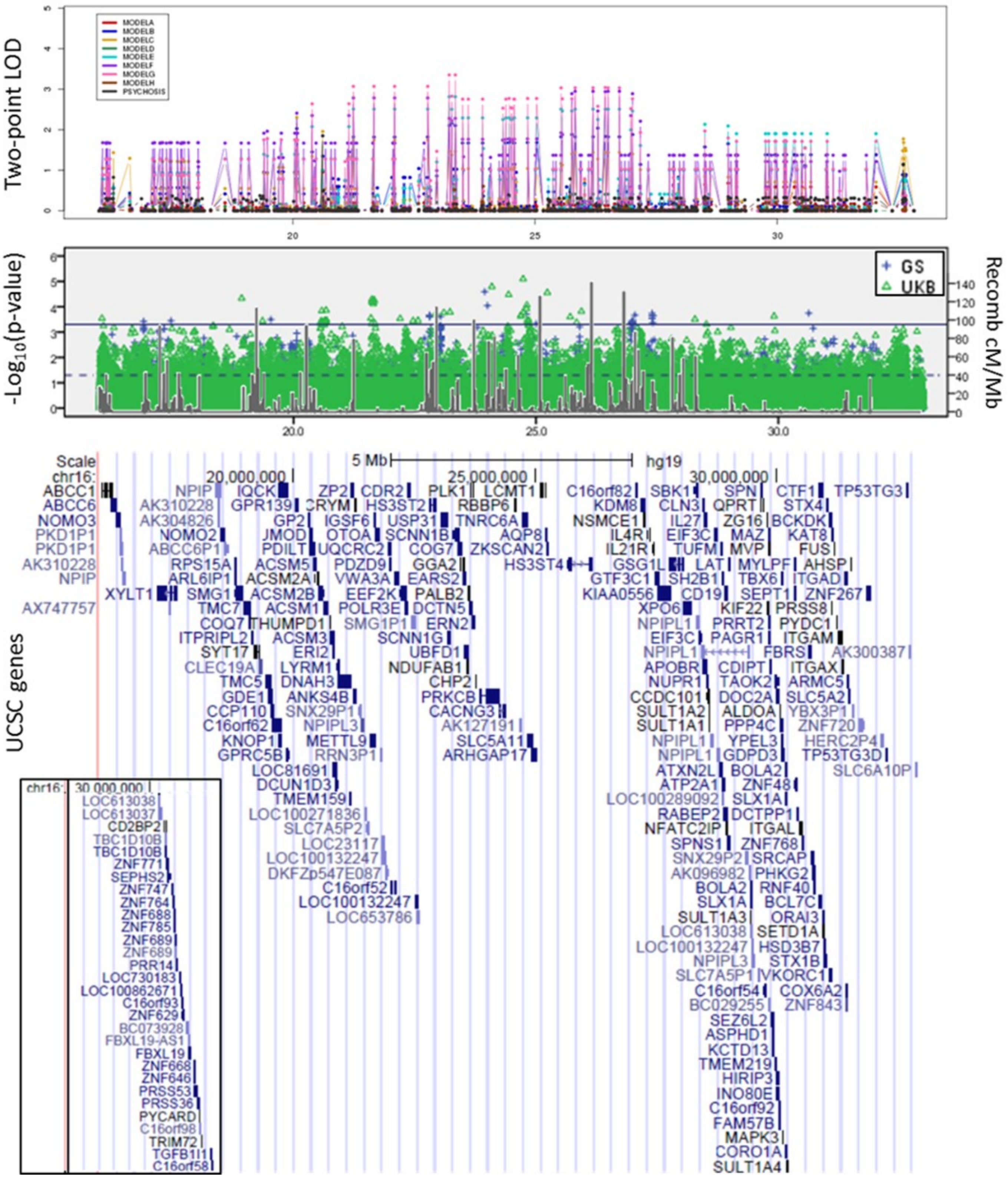

Supplementary Figure 7a-e. Association summaries: a) chr1q; b) chr11q1; c) chr11q2; and d) chr5q. Top: UCSC genes. Bottom: GS:SFHS and UKB association results for affective disorder and multiple related traits, left axis: --log(p-values), right axis: recombination rate cM/Mb GBR 1000 Genomes (grey line) are plotted against Mb position.

7a. Chr1q – chr1: 223000000-225000000

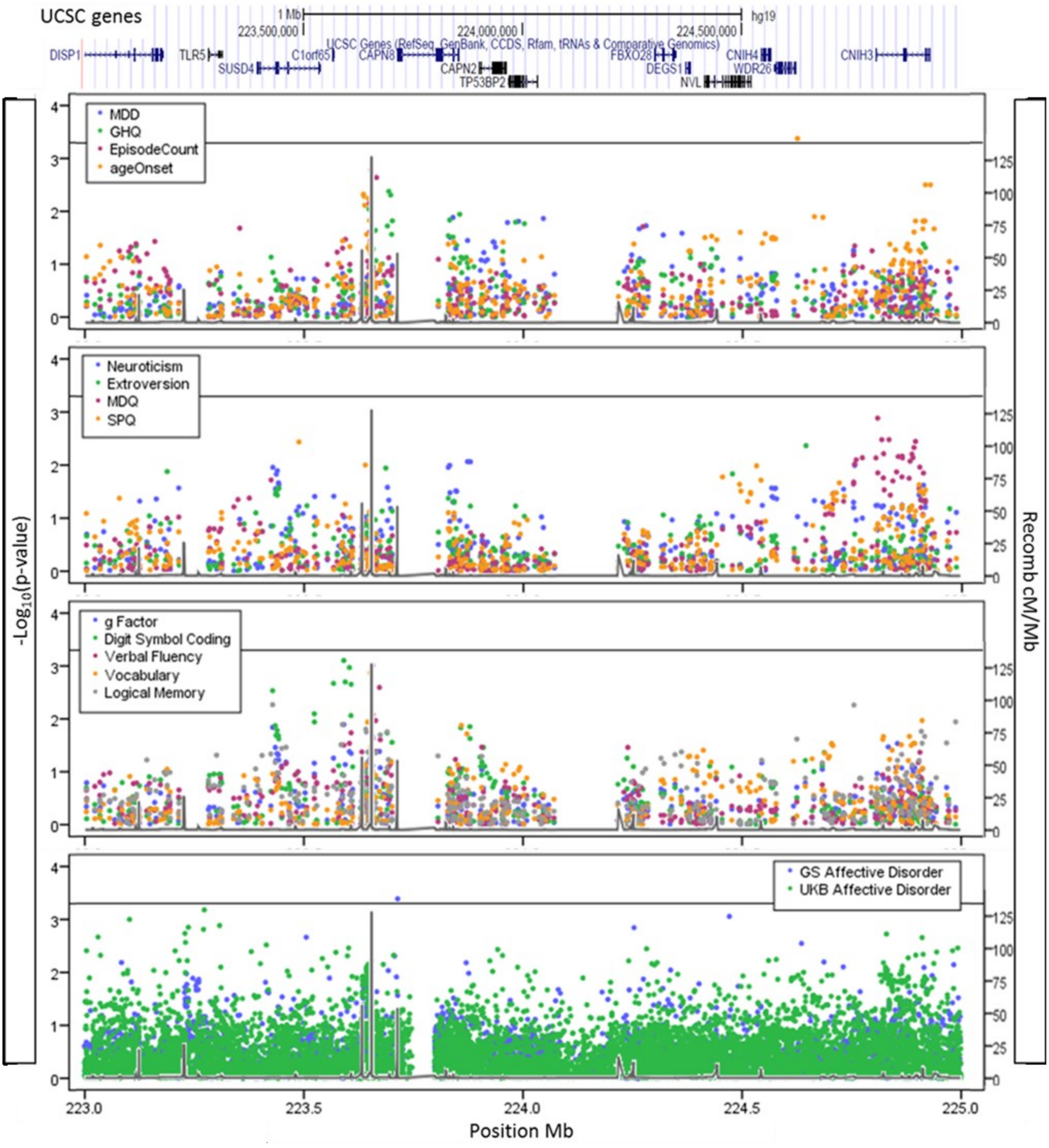

7b. Chr11q1 – chr11: 87500000-89000000

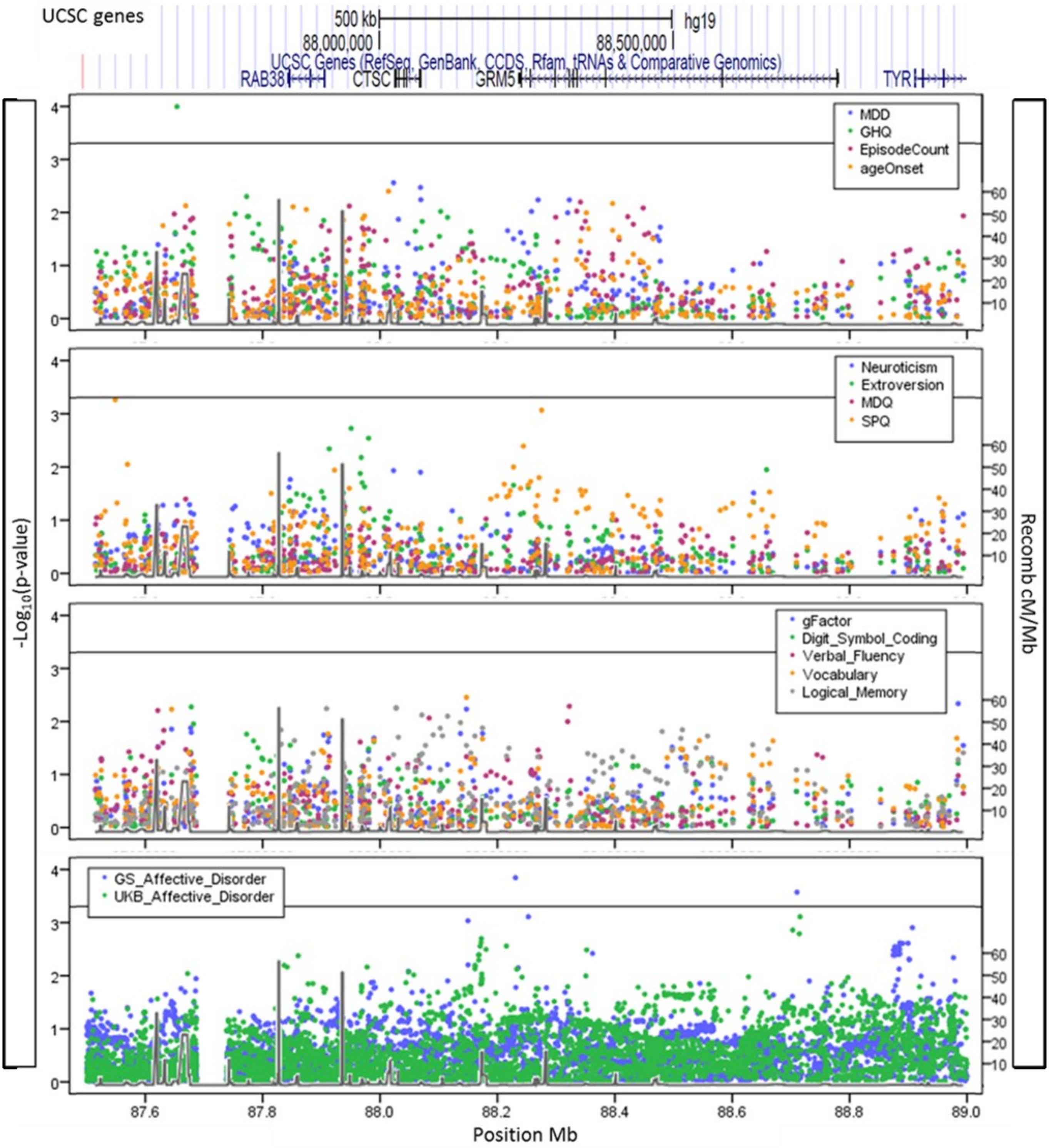

7c. Chr11q2 – chr11: 98500000-100500000

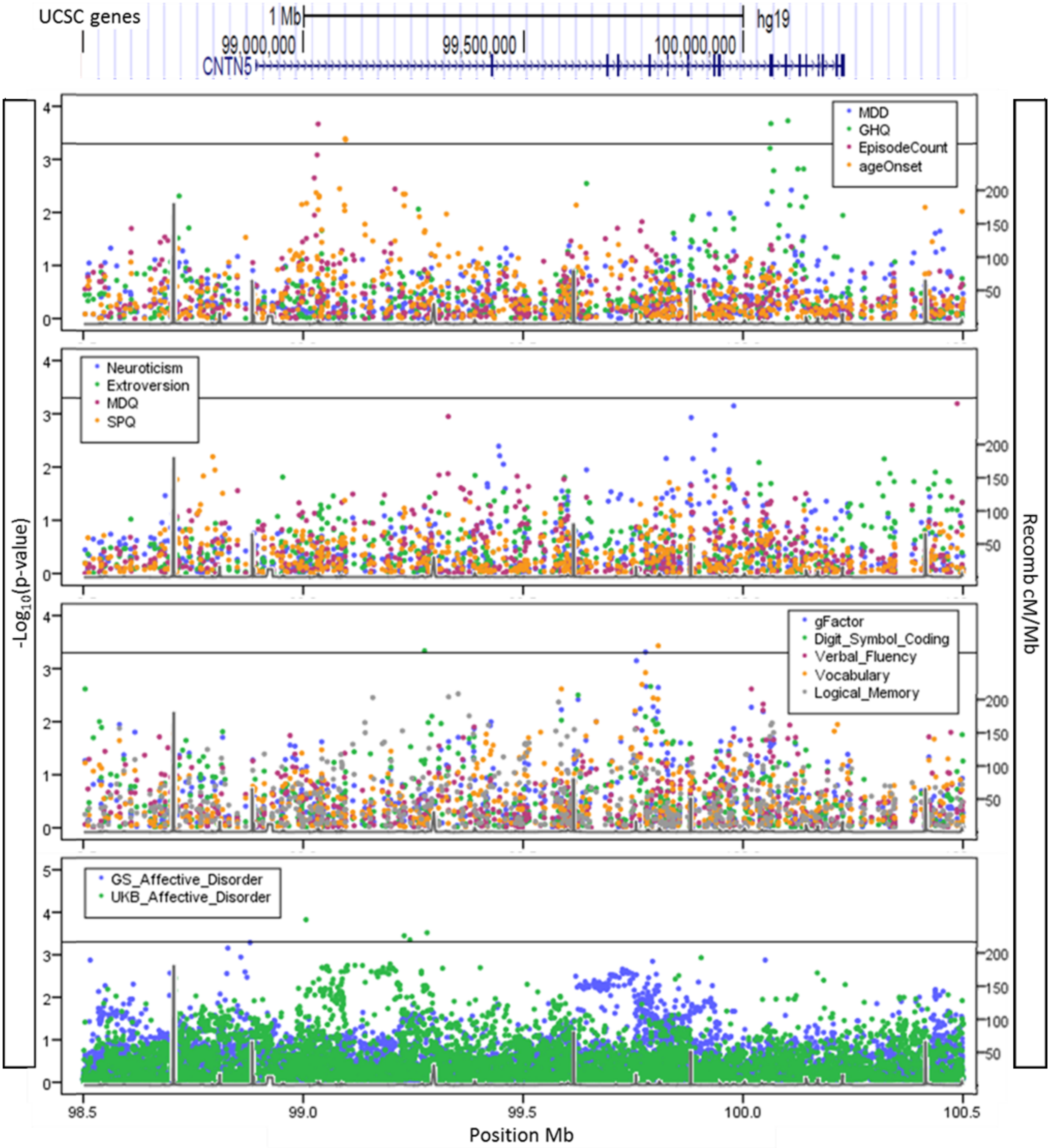

7d. Chr5q – chr5: 58000000-67000000

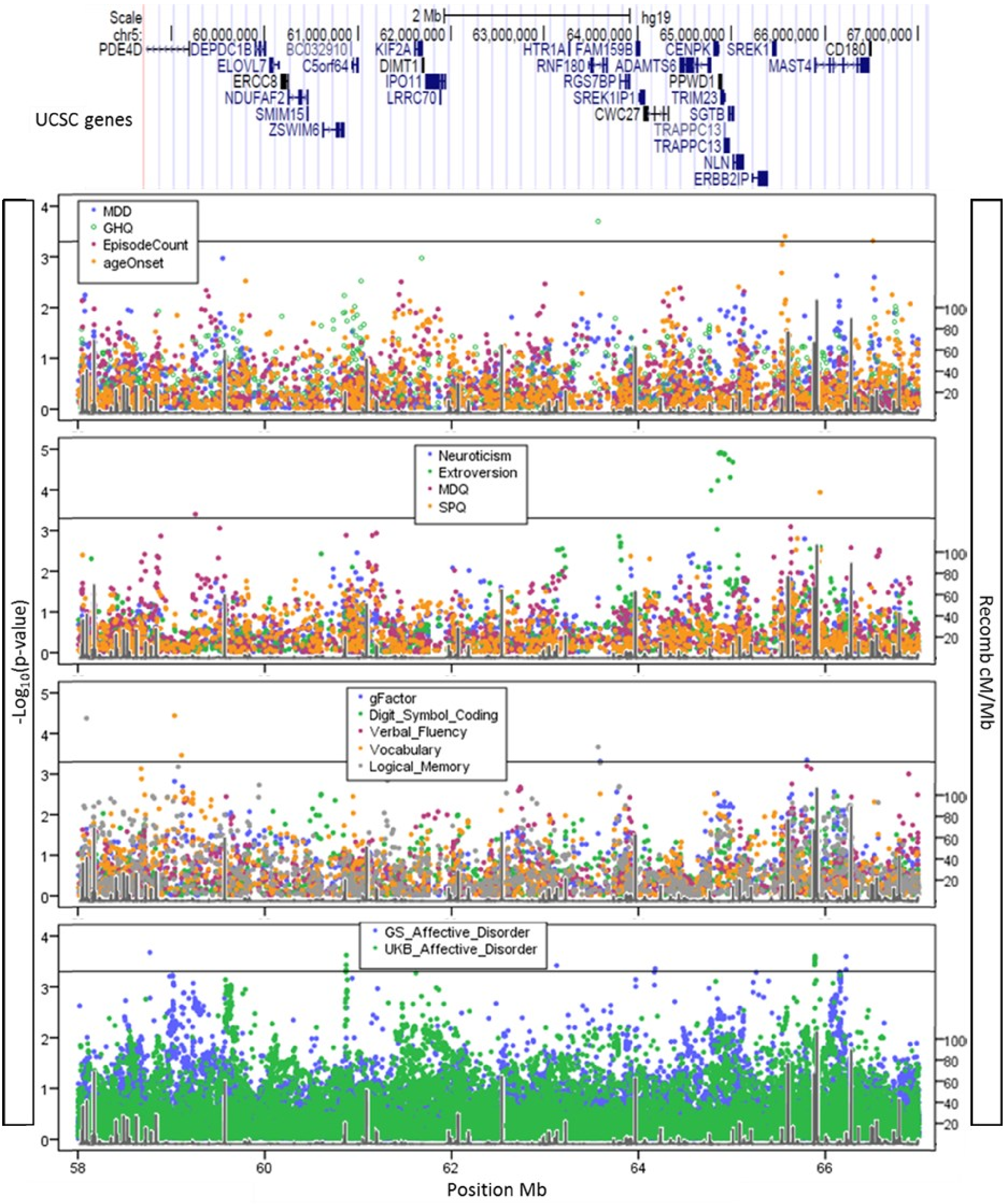

Supplementary Figure 8a-e. Association summaries: a) chr1p; b) chr2p; c) chr3q; d) chr4q; and e) chr16q. Top: UCSC genes. Bottom: GS:SFHS and UKB association results for affective disorder and multiple related traits, left axis: --log(p-values), right axis: recombination rate cM/Mb GBR 1000 Genomes (grey line) are plotted against Mb position.

8a. Chr1p – chr1: 69000000-71000000

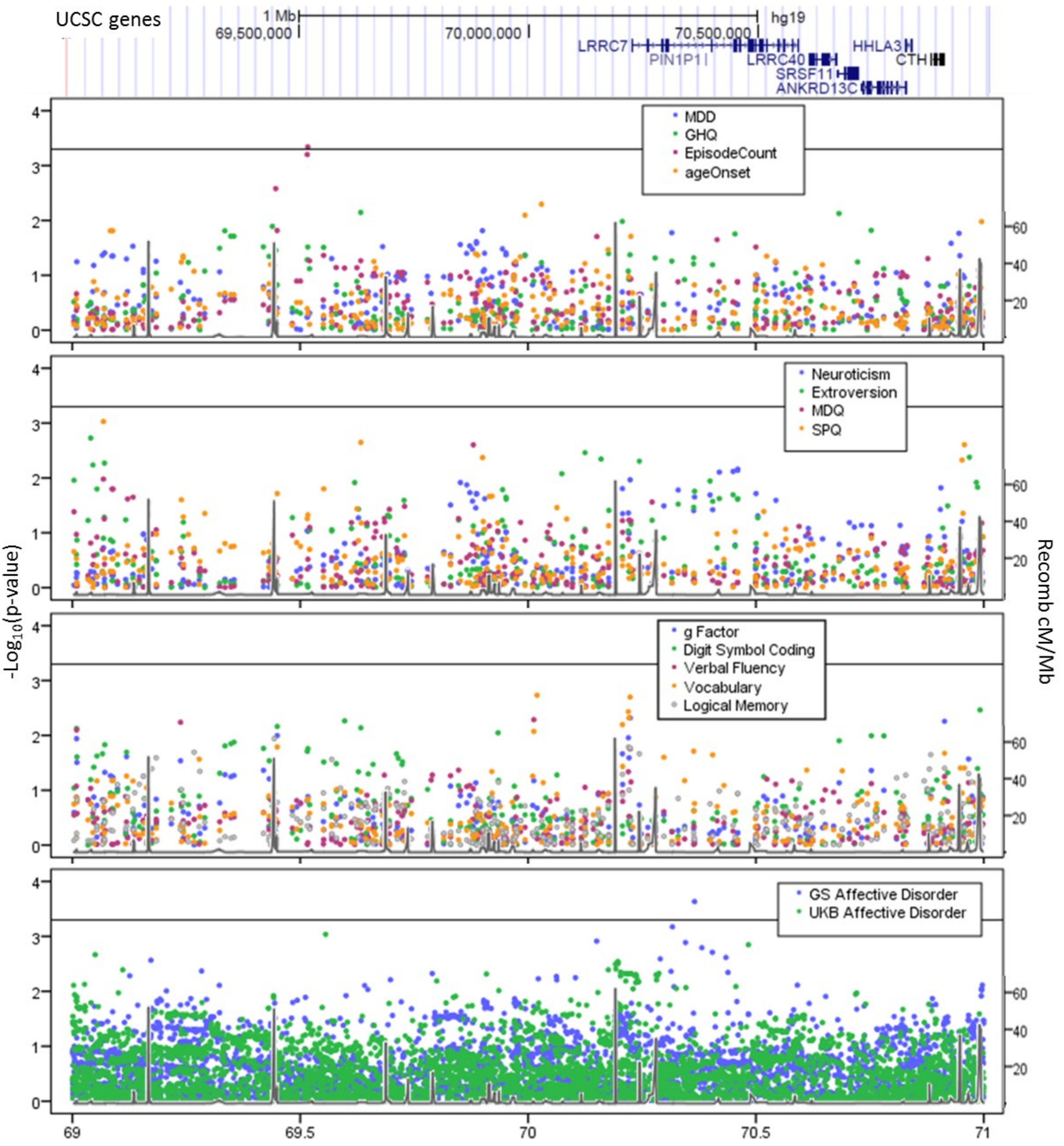

8b. Chr2p – chr2: 17500000-18500000

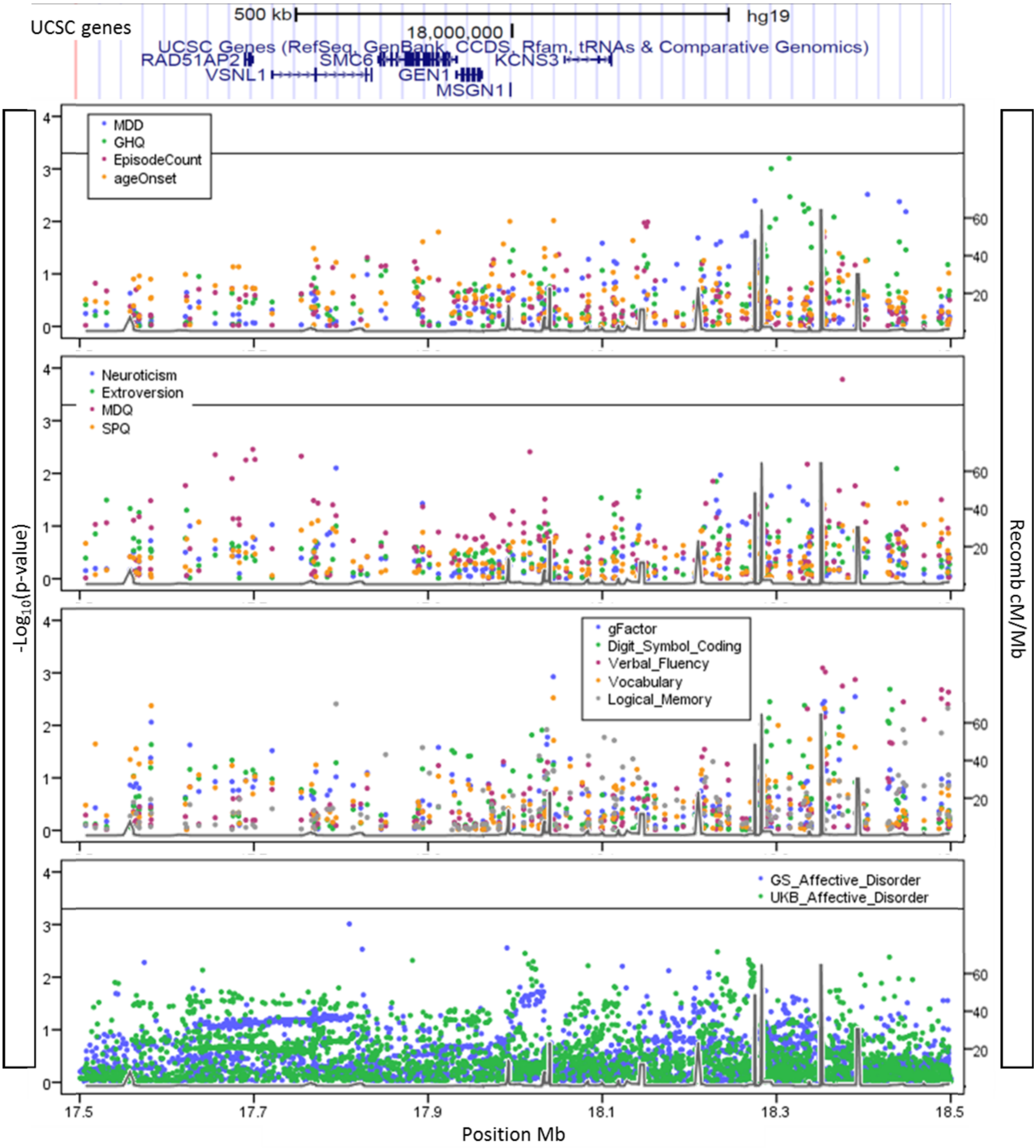

8c. Chr3q – chr3: 167000000-172000000

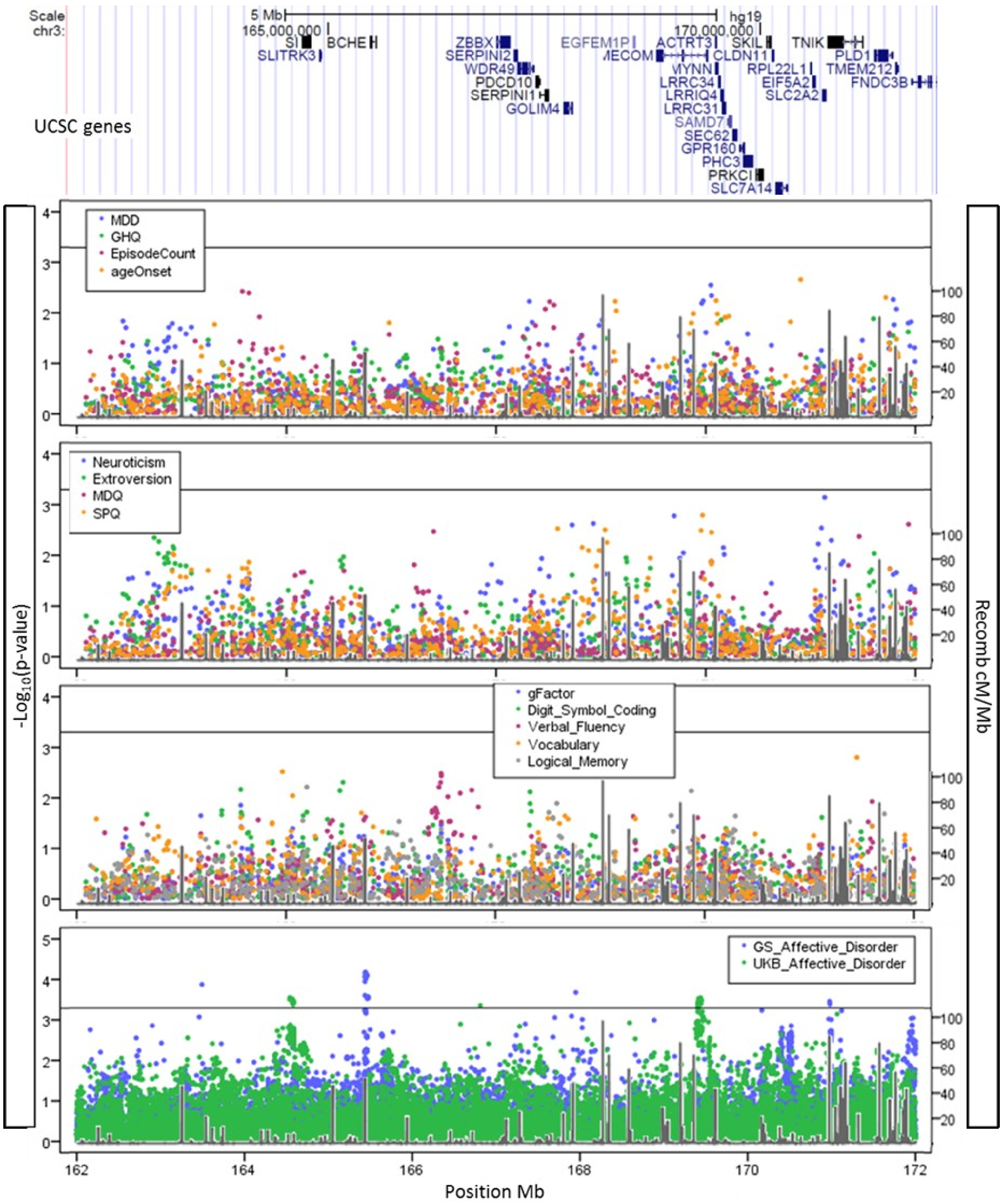

8d. Chr4q – chr4: 158000000-164000000

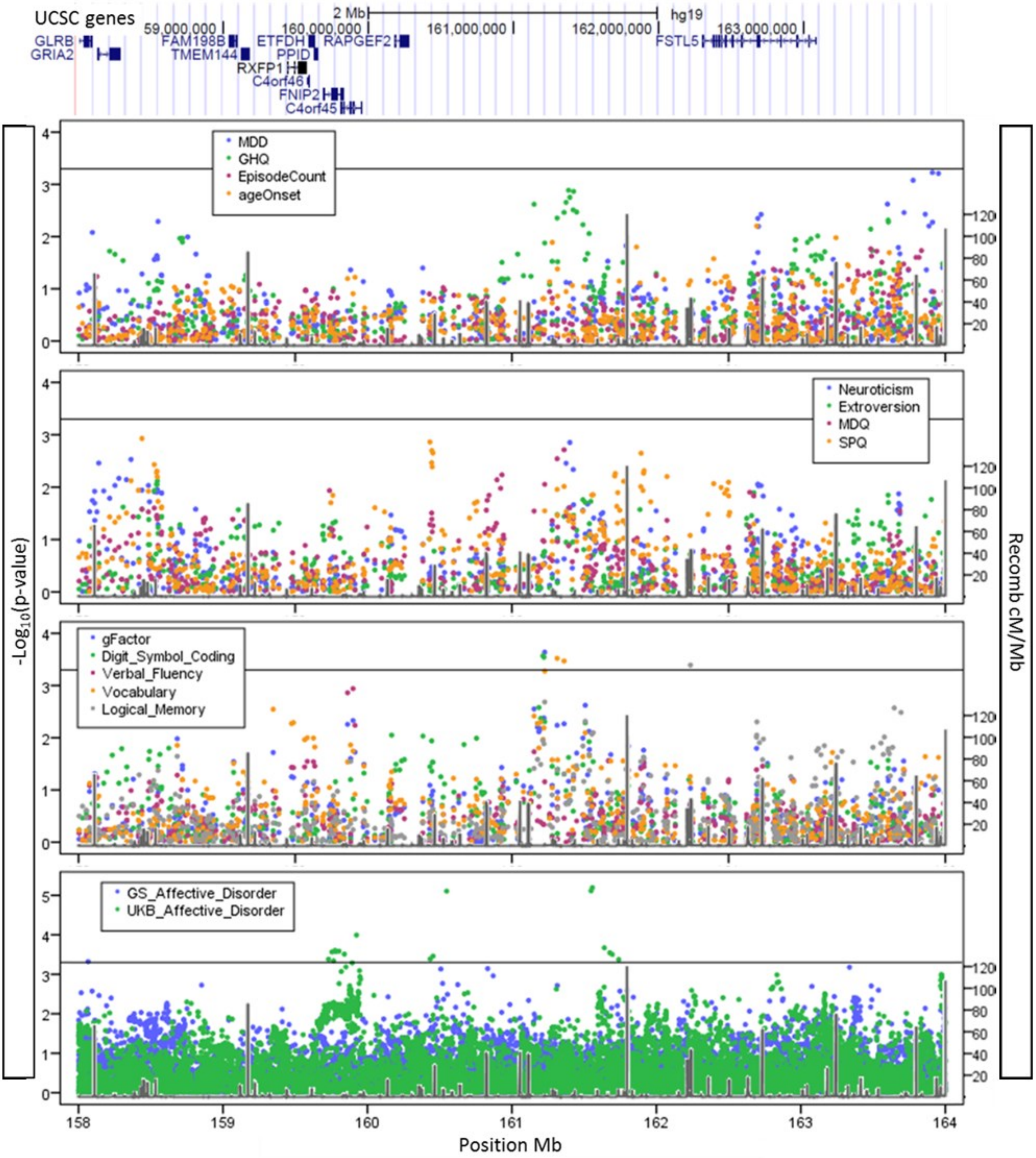

8e. Chr16p – chr16: 21000000-27000000

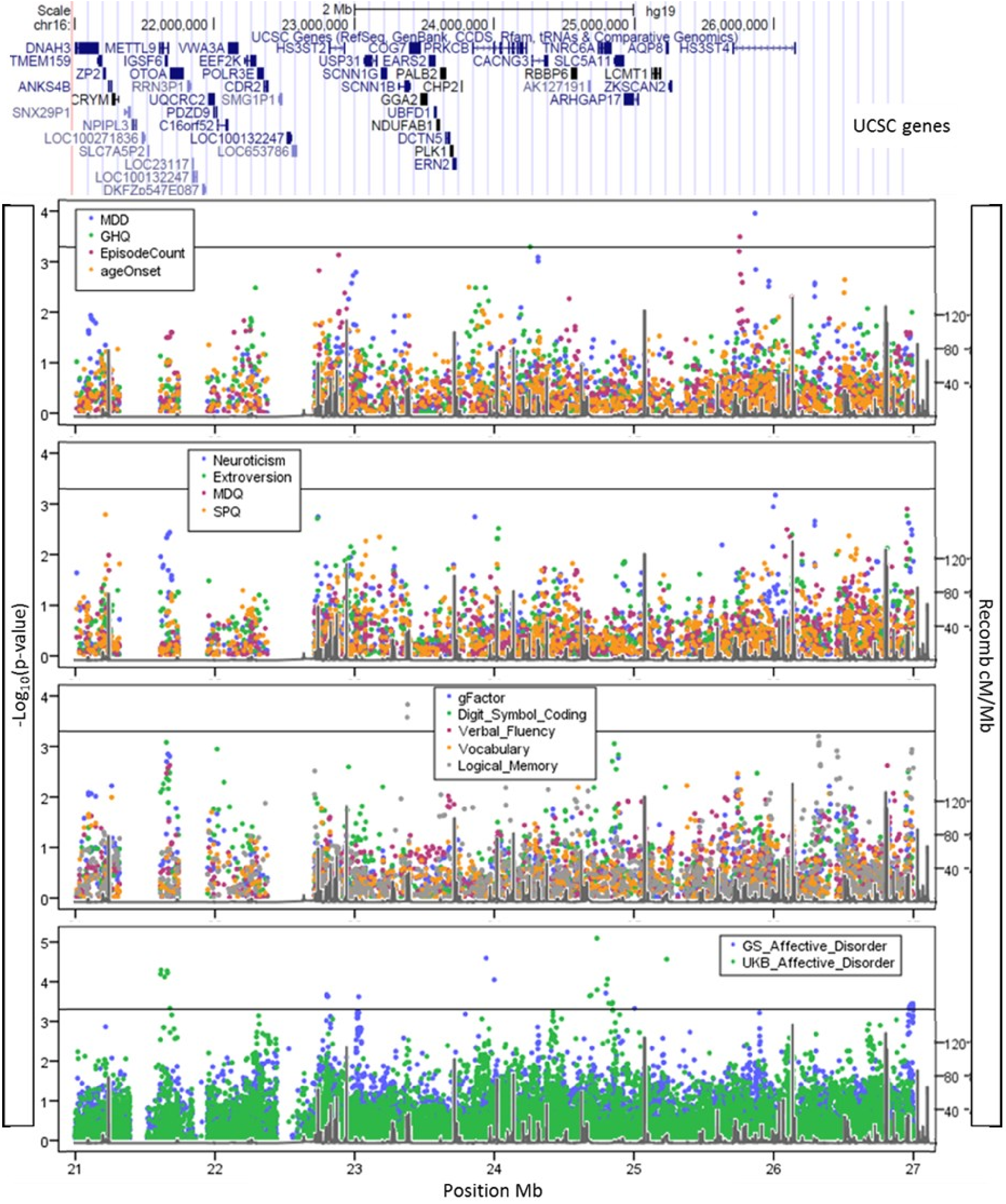

Supplement: Supplementary file 1 — Supplementary Information [file 41380_2018_87_MOESM1_ESM.pdf]
